# 5’tRNA-derived fragments modulate β-cell homeostasis and islet macrophage activation in type 2 diabetes

**DOI:** 10.1038/s41467-026-72641-z

**Published:** 2026-05-04

**Authors:** Cristina Cosentino, Rémy Klein, Véronique Menoud, Claudiane Guay, Elena Aiello, Stefano Auddino, Gianfranco Di Giuseppe, Gea Ciccarelli, Alessandra Galli, Francesco Alabiso, Eleonora Mangano, Flora Brozzi, Karim Bouzakri, Stefania D’Adamo, Silvia Cetrullo, Giuseppe Quero, Andrea Mari, Sergio Alfieri, Andrea Giaccari, Teresa Mezza, Francesco Dotta, Guido Sebastiani, Romano Regazzi

**Affiliations:** 1 Department of Fundamental Neuroscience, University of Lausanne, Lausanne, Switzerland; 2 Dipartimento di Scienze Mediche, Chirurgiche e Neuroscienze, University of Siena, Siena, Italy; 3 Department of Medical and Surgical Sciences, Internal medicine, Endocrinology and Diabetology Unit, Fondazione Policlinico Universitario Agostino Gemelli IRCCS, Rome, Italy; 4 Department of Translational Medicine and Surgery, Università Cattolica del Sacro Cuore, Rome, Italy; 5 Department of Biomedical and Neuromotor Sciences, University of Bologna, Bologna, Italy; 6 Institute for Biomedical Technologies, National Research Council, Milan, Italy; 7 UMR DIATHEC, EA 7294, Centre Européen d’Etude du Diabète, Université de Strasbourg, Fédération de Médecine Translationnelle de Strasbourg, Strasbourg, France; 8 Istituto Nazionale per le Ricerche Cardiovascolari, Bologna, Italy; 9 Health Sciences and Technologies-Interdepartmental Center for Industrial Research (CIRI-SDV), University of Bologna, Bologna, Italy; 10 Department of Medical and Surgical Sciences, Digestive Surgery Unit Fondazione Policlinico Universitario Agostino Gemelli IRCCS, Rome, Italy; 11 Institute of Neuroscience, National Research Council, Padua, Italy; 12 Department of Biomedical Sciences, University of Lausanne, Lausanne, Switzerland

**Keywords:** Mechanisms of disease, Type 2 diabetes, Monocytes and macrophages, Non-coding RNAs

## Abstract

Obesity and diabetes impose chronic stress on pancreatic β-cells, while reprogramming of islet-resident macrophages (iMACs) accelerates dysfunction. Here, we identify transfer RNA-derived fragments (tRFs) as previously unrecognized mediators of islet remodeling under metabolic stress. 5’tRF^Glu(CTC)^ and 5’tRF^Gly(GCC)^ are elevated in β-cells and iMACs from db/db mice and in islets from individuals with type 2 diabetes; 5’tRF^Glu(CTC)^ also rises in prediabetes and inversely correlates with insulin secretion. Lipotoxicity triggers 5’tRF biogenesis, and targeted inhibition of 5’tRF^Glu(CTC)^ preserves β-cell viability and function under palmitate exposure. In a β-cell/macrophage co-culture model, β-cell contact shapes a distinct iMAC-like phenotype that shifts after palmitate treatment. Inhibiting 5’tRF^Glu(CTC)^ in iMAC-like cells prevents their activation switch and protects β-cells from lipotoxicity. Mechanistically, 5’tRF^Glu(CTC)^ interacts with RNA-binding proteins to control immune activation, extracellular matrix remodeling, and oxidative stress pathways. These findings position tRFs as central effectors of cellular stress responses in both endocrine and immune cells.

## Abstract (web summary)

Here the researchers report that 5’tRF^Glu(CTC)^, a tRNA-derived fragment, is associated with diabetes and mediates pancreatic beta cell and macrophage responses to nutritional stress. targeted inhibition of 5’tRF^Glu(CTC)^ preserves beta cell viability and function under lipotoxic exposure.

## Introduction

Obesity, characterized by excessive caloric intake and increased adiposity, represents a significant risk factor for type 2 diabetes (T2D) development. Pancreatic β-cells play a pivotal role in maintaining glucose homeostasis by secreting insulin in response to rising blood glucose levels. However, inappropriate nutritional conditions trigger endoplasmic reticulum (ER) stress and oxidative stress, which collectively drive β-cell dysfunction and, eventually, apoptosis. Simultaneously, the energy imbalance established during obesity activates innate immune responses as an adaptive mechanism to cope with the increased nutrient load. Indeed, expansion of islet macrophages (Mφs) was observed in T2D patients and in rodent models of obesity and T2D^1–4^. Under physiological conditions, islet resident Mφs (iMACs) exhibit a unique transcriptional profile characterized by the expression of pro-inflammatory markers, such as IL-1β, that sustain proper β-cell function^5^. However, a high-fat diet (HFD) induces iMAC expansion accompanied by profound transcriptional remodeling: while certain genes associated with the pro-inflammatory profile are upregulated, others are notably downregulated^3^. In addition to these changes, β-cell death was recently reported to activate a reparative and anti-inflammatory state in Mφs, marked by the secretion of Insulin-like Growth Factor 1 (IGF1), which enhances β-cell function^6^. These findings underscore the dynamic and multifaceted role of Mφs in islet health and T2D pathogenesis.

Despite significant progress in understanding islet physiopathology, the molecular mechanisms that regulate β-cell stress responses and the metabolic reprogramming of islet-resident macrophages (iMACs) during obesity remain poorly defined. In particular, the signals mediating the crosstalk between β-cells and iMACs are still largely unknown.

Recent genome-wide association studies and high-throughput sequencing approaches have pointed to a potentially crucial role of transfer RNA (tRNA) dynamics in maintaining pancreatic β-cell homeostasis. Traditionally known for their role in protein synthesis, tRNAs are now recognized as being tightly regulated by both nutritional and environmental cues. Post-transcriptional modifications and enzymatic cleavage of tRNAs have been linked to cellular stress responses and the development of various diseases^7,8^.

tRNA cleavage gives rise to tRNA-derived fragments (tRFs), a novel class of small non-coding RNAs that regulate a wide range of cellular processes, including gene expression, mRNA translation, cell differentiation, and apoptosis^9^. tRFs are classified according to their site of origin within the tRNA molecule. Endonucleatic cleavage at the anticodon loop generates 5’ or 3’ tRNA-halves (28-33 nt), which are predominantly produced by the endonuclease Angiogenin in response to cellular stress^10–12^. 5’tRNA-halves bearing a terminal oligoguanine (TOG) motif (from tRNA^Ala^, tRNA^Cys^, tRNA^Val^) have been shown to inhibit translation by interacting with YB-1 and preventing eIF4F complex assembly^12^. In addition, 5’tRNA-halves generated through IRE1α-mediated cleavage of tRNA^Gly(GCC)^ are produced upon ER-stress in cancer cells and have been implicated in the regulation of mRNA isoform biogenesis^13^. Cleavage at other sites within tRNAs gives rise to 5’, 3’ or internal tRFs of variable lengths (15-50 nt)^14^. The biogenesis of 5’tRNA^Glu(CTC)^-halves has been associated with cellular responses to viral infection and is proposed to play a role in mediating inflammatory responses^15^.

Notably, loss-of-function mutations in the tRNA-modifying enzyme TRMT10A have been associated with a syndrome marked by microcephaly and early-onset diabetes. Impaired TRMT10A function leads to altered tRF biogenesis, contributing to β-cell oxidative stress and apoptosis^16,17^. Moreover, we have previously shown that tRFs are dynamically regulated during β-cell maturation^18^ and in pancreatic islets of rodent models predisposed to diabetes^19^.

Despite these insights, the functional role and mechanisms of action of tRFs in β-cells remain unclear. In addition, their regulation and function in iMACs and in the communication between iMACs and β-cells during obesity has not been investigated so far.

This study provides evidence that obesity and diabetes induce a specific modulation of tRFs in both β-cells and iMACs. Indeed, 5’tRFs derived from tRNA^Glu(CTC)^ and tRNA^Gly(GCC)^ were found to be induced in both cell types, an effect recapitulated in vitro by exposure to saturated fatty acids. Blockade of 5’tRF^Glu(CTC)^ in islet cells or specifically in macrophages had protective effects against lipotoxicity. The analysis of the 5’tRF^Glu(CTC)^ interactome revealed an association with RNA-binding proteins involved in mRNA processing, splicing and translation. In agreement with this finding, blockade of this tRNA fragment induced transcriptional and translational changes.

## Results

### Obesity and type 2 diabetes induce changes in 5’tRFs in β-cells and iMACs

**Fig. 1:**
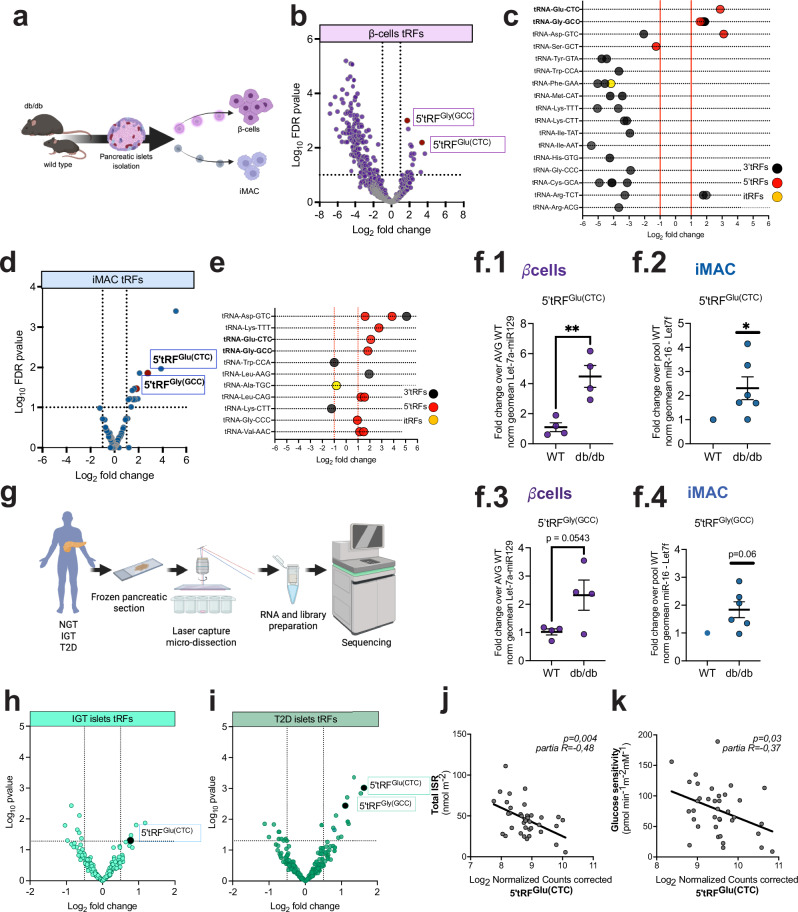
tRF profile of pancreatic islet cells in obesity and T2D. Pancreatic islets were obtained from db/db mice and wild-type controls; *β*-cells and iMACs were isolated by FACS (**a**, Created in BioRender. Cosentino, C. (2026) https://BioRender.com/ky4h37h) and small RNA sequencing was performed (**b-e**). Volcano plots show differentially expressed tRFs in db/db *β*-cells (**b**) and iMAC (**d**) compared to controls, *p* values were derived from a negative binomial generalized linear model and adjusted for multiple testing using the Benjamini–Hochberg FDR method (two-sided tests) (*n* = 4 independent preparations: pooled wt and 6 db/db mice). tRNA isodecoder of origin and the different classes of tRFs are described in (**c**, **e**): gray dots represent fragments from 3’tRNA, yellow from internal tRNA and red from 5’tRNA regions. 5’tRF^Glu(CTC)^ and 5’tRF^Gly(GCC)^ levels were detected by qPCR and normalized with the expression of Let-7e in *β*-cells (*n* = 4 wt and 4 db/db mice, **f1, f3**) or miR-16 in iMAC (*n* = 6 pooled wt and 6 db/db mice, **f2, f4**) Data in f1-4 are presented as mean values ± SEM, *p < 0.05 (f.2 *p* = 0.0312), **p < 0.01 (f.1 *p* = 0.0051) by unpaired two-sided Wilcoxon signed rank test or Student *t* test, respectively, source data are provided as a Source Data file. Human islets were collected by laser capture microdissection from pancreatic sections (**g**, Created in BioRender. Cosentino, C. (2026) https://BioRender.com/ky4h37h). Patients were categorized based on oral glucose tolerance: normal glucose tolerance (NGT, *n* = 12 < 140 mg/dL 2 h plasma glucose); impaired glucose tolerance (IGT *n* = 13 140–199 mg/dL 2 h plasma glucose) and type 2 diabetes (T2D *n* = 11 ≥ 200 mg/dL 2 h plasma glucose). Volcano plots recapitulate the changes in tRF levels in IGT versus NGT islets (**h**) and T2D versus NGT islets (**i**); *p* values were derived from a negative binomial generalized linear model and adjusted for multiple testing using the Benjamini–Hochberg FDR method (two-sided tests). The association of 5’tRF^Glu(CTC)^ levels with the clinical parameters total insulin secretion rate (ISR) (**j**) and glucose sensitivity (**k**) was performed using linear models. The association of Log_2_-scaled tRF normalized counts with clinical/metabolic parameters was corrected for the covariates (Age, Sex, BMI). Statistical significance of the association was assessed using two-sided *t*-tests on the regression coefficients, and regressions with *p* values < 0.05 were considered statistically significant.

To investigate whether tRNA cleavage and consequently the pool of tRFs is affected by obesity and diabetes, we isolated the pancreatic islets of 8-week-old overweight and hyperglycemic db/db mice and of age-matched lean and normoglycemic wild-type controls (Figure S1a, b). Db/db mice display hyperphagia due to a mutation in the leptin receptor gene, and develop obesity and diabetes. It was previously shown that at 8 weeks of age, in the initial phases of the disease, islet macrophages (iMAC) of db/db mice expand and undergo transcriptional remodeling^6^. Since both iMACs and β-cells are highly sensitive to nutritional stress and to the diabetogenic microenvironment, we purified the two cell populations by FACS (Fig. 1a and Figure S1c). The β-cell enriched fraction, sorted based on autofluorescence showed a marked enrichment of insulin mRNA (*Ins*) and did not contain detectable glucagon-expressing cells or cells expressing the immune marker *Tnf-α*. (Figure S1d–f). Glucagon-expressing cells (*Gcg*) were recovered exclusively in the ungated fraction together with low levels of insulin-expressing cells and a very low number of immune cells. The Cd11c^+^ iMAC population did not contain any insulin- or glucagon-expressing cells. Although the effect was not statistically significant, the number of isolated iMACs tended to increase in db/db mouse islets compared to those of wild-type mice (Figure S1g). Characterization of the tRF profile of FACS-sorted cells led to the identification of 152 tRFs displaying significant changes in β-cells and 21 in iMACs of db/db mice compared to wild-type controls (FC > 2, FDR *p* < 0.1) (Fig. 1b, d). After removal of redundant sequences with differences of 1–2 nucleotides at the cleavage site, we identified a group of tRFs derived from 17 cytosolic tRNA isoacceptors in β-cells (Fig. 1c). Analysis of tRF classes highlighted the downregulation of 3’tRFs derived from 14 different isoacceptors and the decrease of only one 5’tRF and one internal tRF. 5’tRFs derived from tRNA^Asp(GTC)^, tRNA^Gly(GCC)^ and tRNA^Glu(CTC)^ were induced in db/db β-cells (Fig. 1c). tRFs from 11 cytosolic tRNA isoacceptors were modulated in iMACs from db/db mice compared to wild type (Fig. 1e). The majority of iMAC modulated tRFs were 5’-derived and were upregulated in db/db mice (Fig. 1e). Three tRFs derived from the 5’end of tRNA^Glu(CTC)^, tRNA^Gly(GCC)^ and tRNA^Asp(CTC)^ were induced in both cell types. The upregulation of 5’tRF^Glu(CTC)^ and 5’tRF^Gly(GCC)^ was validated by qPCR (Fig. 1f1-4), while qPCR for 5’tRF^Asp(CTC)^ was not conclusive due to the presence of multiple melting curves. Interestingly, we previously demonstrated that 5’tRF^Glu(CTC)^ is repressed during islet cell maturation and may regulate newborn β-cell function^18^.

We next analysed 5’tRF modulation across different mouse models of obesity and T2D (Figure S2). 16-week-old db/db mice exhibited obesity and hyperglycemia, modeling advanced stages of T2D (Figure S2a, b). In whole islets from db/db mice, both 5’tRF^Glu(CTC)^ and 5’tRF^Gly(GCC)^ were increased compared to heterozygous controls (Figure S2c, d), confirming our observations in younger leptin-receptor mutated mice. In contrast, ob/ob mice, which carry a mutation in the leptin gene, showed increased body weight but normal basal blood glucose levels (Figure S2e, f). Accordingly, 5’tRFs were not altered in whole islets from ob/ob mice compared to heterozygous controls (Figure S2g). Finally, we examined tRF expression in a diet-induced obesity (DIO) model. Mice fed a high-fat diet (HFD) displayed a milder increase in body weight than the genetic models and maintained normal blood glucose levels (Figure S2h, i). Consistent with the findings in normoglycemic ob/ob mice, the 5’tRF levels remained unchanged in the DIO model in both whole islets and FACS-sorted β-cells, and in iMACs compared with age-matched chow diet (CD) controls (Figure S2j–o).

To analyze the modulation of tRFs in the islets of human living donors, we collected RNA from islets isolated by laser capture microdissection (LCM) from *n* = 12 normal glucose tolerant (NGT), *n* = 13 impaired glucose tolerant (IGT) and *n* = 11 T2D diagnosed patients (Fig. 1g; Table S1). Small RNA sequencing and tRF profiling identified 8 tRFs significantly changed in the IGT group (Fig. 1h) and 13 tRFs modulated in the T2D (Fig. 1i) compared to the NGT controls (FC > 1.5, *p* < 0.05). We found that the 5’tRF^Glu(CTC)^ was significantly upregulated in both IGT and T2D groups (Fig. 1h, i), while 5’tRF^Gly(GCC)^ was increased only in T2D islets (Fig. 1i). Notably, these tRFs present 100% of sequence homology between mouse and human. Our findings suggest that tRF modulation in islet cells occurs during T2D development and is conserved between species.

Association studies revealed that islet 5’tRF^Glu(CTC)^ levels were negatively associated to basal and total insulin secretion rates and glucose sensitivity (Fig. 1j, k, Figure S1.h-I, Table S2). Notably, this association was mainly driven by the IGT group (Figure S1.h2 and i2). In contrast, no correlations were found with basal or mean glucose levels during the OGTT (Table S2). These findings suggest that islet 5’tRF^Glu(CTC)^ levels may directly impair in vivo insulin secretory capacity and reflect early decline of β-cell function, independently of both glucose control and glucose tolerance status.

### 5’tRF^Glu(CTC)^ and tRF^Gly(GCC)^ are induced by exposure to saturated free fatty acids

**Fig. 2:**
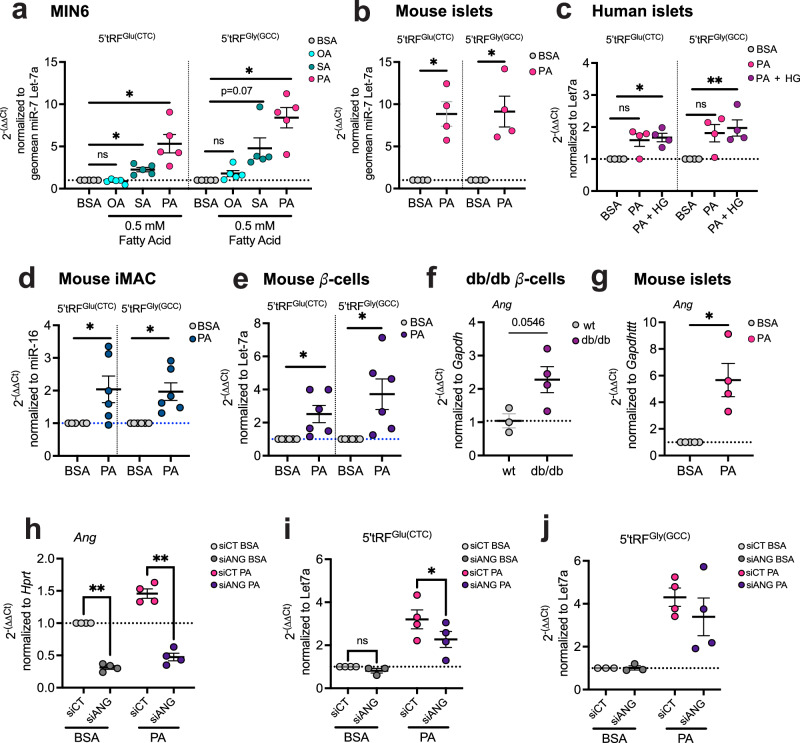
5’tRFs from tRNA^Glu(CTC)^ and tRNA^Gly(GCC)^ are induced by fatty acids in *β*-cells and iMACs. MIN6 cells were treated with 0.5 mM of the unsaturated fatty-acid oleate (OA, blue dots) or the saturated fatty acids stearate (SA, green dots) and palmitate (PA, pink dots) for 48 h and the levels of 5’tRFs were measured by qPCR (*n* = 5 independent experiments); 5’tRFs levels were normalized against basal 0.75% BSA (gray dots) treatment condition (**a**). The induction of 5’tRF^Glu(CTC)^ and 5’tRF^Gly(GCC)^ was assessed in dispersed mouse islets treated with PA (pink dots) (**b**, *n* = 4 independent experiments) and human islets treated with PA alone (pink dots) or in combination with 20 mM Glucose (purple dots) for 48 h (**c**, *n* = 4 independent experiments). Upon exposure to palmitate, mouse islets were dispersed, iMACs and *β*-cells were isolated by FACS and tRFs were measured in the two cell populations (**d**, **e**, *n* = 6 independent experiments). In (**d**) blue dots and in (**e**) purple dots are measures in PA condition, while gray dots represent measures in BSA. Angiogenin (*Ang*) expression was assessed by qPCR in FACS-sorted *β*-cells from db/db (purple dots) and wild type (wt, gray dots) mice (**f**, *n* = 3 wt, *n* = 4 db/db mice) and dispersed mouse islets treated with PA (pink dots) or BSA (gray dots) (**g**, *n* = 4 independet experiments). Knockdown of Angiogenin was achieved by siRNA transfection (siANG) in MIN6 cells (**h**–**j**, *n* = 4 independent experiments). The levels of *Ang*, 5’tRF^Glu(CTC)^ and 5’tRF^Gly(GCC)^ were assessed by qPCR upon transfection with siRNA control (siCT) in BSA (gray dots) and PA (pink dots) or siANG in BSA (dark gray dots) and PA (purple dots) (**h**–**j**, *n* = 4 independent experiments). Data in (**a**–**j**) are presented as mean values ± SEM, *p < 0.05, **p < 0.01 by one-way ANOVA with Sidak correction for multiple comparisons (in **a**, **c**, **h**–**j**), by two-sided paired Student *t* test (in **b**, **d**, **e**, **g**) and by two-sided unpaired Student *t* test (in **f**). Exact *p* values in (**a**) 5’tRF^Glu(CTC)^ SA vs BSA *p* = 0.0268 and PA vs BSA *p* = 0.0497, 5’tRF^Gly(GCC)^ PA vs BSA *p* = 0.0081; in (**b**) 5’tRF^Glu(CTC)^ PA vs BSA *p* = 0.0127, 5’tRF^Gly(GCC)^ PA vs BSA *p* = 0.0211; in (**c**) 5’tRF^Glu(CTC)^ PA + HG vs BSA *p* = 0.0286, 5’tRF^Gly(GCC)^ PA + HG vs BSA *p* = 0.0215; in (**d**) 5’tRF^Glu(CTC)^ PA vs BSA *p* = 0.050, 5’tRF^Gly(GCC)^ PA vs BSA *p* = 0.0156; in (**e**) 5’tRF^Glu(CTC)^ PA vs BSA *p* = 0.033, 5’tRF^Gly(GCC)^ PA vs BSA *p* = 0.0324; in (**g**) PA vs BSA *p* = 0.0331; in (**h**) BSA siANG vs siCT *P* = 0.0039, PA siANG vs siCT p = 0.0014; in (**i**) PA siANG vs siCT *p* = 0.046. Source data are provided as a Source Data file.

We next investigated whether in vitro exposure to fatty acids—mimicking the lipotoxic stress associated with obesity-linked diabetes— recapitulates the induction of the tRFs observed in vivo. The unsaturated fatty-acid oleate (OA) did not modulate the tRF levels in MIN6 β-cells (Fig. 2a). We observed a significant increase of 5’tRF^Glu(CTC)^ induced by the saturated free fatty acids stearate (SA) and palmitate (PA) (Fig. 2a). Interestingly, PA elicited a greater rise of 5’tRF^Glu(CTC)^ and induced also the 5’tRF^Gly(GCC)^ levels compared to the less lipotoxic stearate (Fig. 2a). This effect was time dependent and was detectable only after 16 h treatment (Figure S3a, b). The induction of these tRFs upon 48 h PA treatment was confirmed in islets derived from wild-type mice and from human cadaveric donors (Fig. 2b, c). qPCR analysis of FACS-sorted β-cells and iMACs from wild-type mice confirmed the induction of the tRFs in both cell types (Fig. 2d, e). While chronic PA exposure induces lipotoxic effects in β-cells^20,21^, acute PA stimulation has been shown to trigger insulin secretion^22,23^. To determine whether 5’tRFs are also involved in acute palmitate effects, we measured their levels in mouse islets subjected to 1 h glucose starvation followed by acute exposure to PA in the presence of either low (2 mM) or high (20 mM) glucose. Neither condition induced the 5’tRFs, supporting their association with chronic lipotoxic stress (Figure S3c, d). Notably, a 3-day wash-out period following 48 h PA treatment restored 5’tRF^Glu(CTC)^ to basal levels and partially reversed the upregulation of 5’tRF^Gly(GCC)^ (Figure S3e, f). In parallel, the expression of the ER-stress marker *Chop*, which was induced after 48 h of PA exposure, was attenuated after the wash-out period (Figure S3g). Altogether, these data indicate that 5’tRF^Glu(CTC)^ and 5’tRF^Gly(GCC)^ are specifically induced under chronic lipotoxic conditions and that their expression is reversible upon removal of the stress.

In mouse and human islet cells, 5’tRF^Glu(CTC)^ fragments are 29-30nt long (Table S3) and are generated by a cleavage adjacent or in the anticodon loop, consistent with a classification as tRNA halves. Angiogenin was previously identified as the major enzyme responsible of tRNA cleavage at the level of the anticodon loop and of the biogenesis of tRF-halves^10,24^. 5’tRF^Gly(GCC)^ fragments identified in our datasets are 26–30nt long (Table S3). The biogenesis of shorter 5’tRFs have been also associated with Angiogenin expression, although other RNAses may participate to the trimming of the cleavage site^24^. We have previously showed that silencing of Angiogenin gene (*Ang*) in islets from newborn rats decreases the levels of 5’tRF^Glu(CTC)18^. To assess whether this enzyme is responsible for the generation of 5’tRF^Glu(CTC)^ and 5’tRF^Gly(GCC)^ under obesity and diabetes conditions, we first measured the expression of *Ang* in our experimental models. We observed that *Ang* mRNA is increased in β-cells of db/db mice compared to wild-type controls (Fig. 2f) and is induced in MIN6 and dispersed mouse islet cells upon PA exposure (Figure S3h and 2g). Moreover, *Ang* expression in mouse islets was reverted to basal levels after 3-day wash-out following PA exposure (Figure S3i) and correlates to the modulation of the fragment. To further confirm the role of Angiogenin in 5’tRF^Glu(CTC)^ and 5’tRF^Gly(GCC)^ biogenesis, we decreased the level of the enzyme using a siRNA (Fig. 2h). We found that transient knockdown of Angiogenin attenuates the induction of 5’tRF^Glu(CTC)^ but not of 5’tRF^Gly(GCC)^ (Fig. 2i, j). These findings validate our previous observations in newborn β-cells and suggest that additional enzymes may be involved in palmitate-dependent tRNA cleavage.

### Blockade of 5’tRF^Glu(CTC)^ attenuates lipotoxic damage in β-cells

**Fig. 3:**
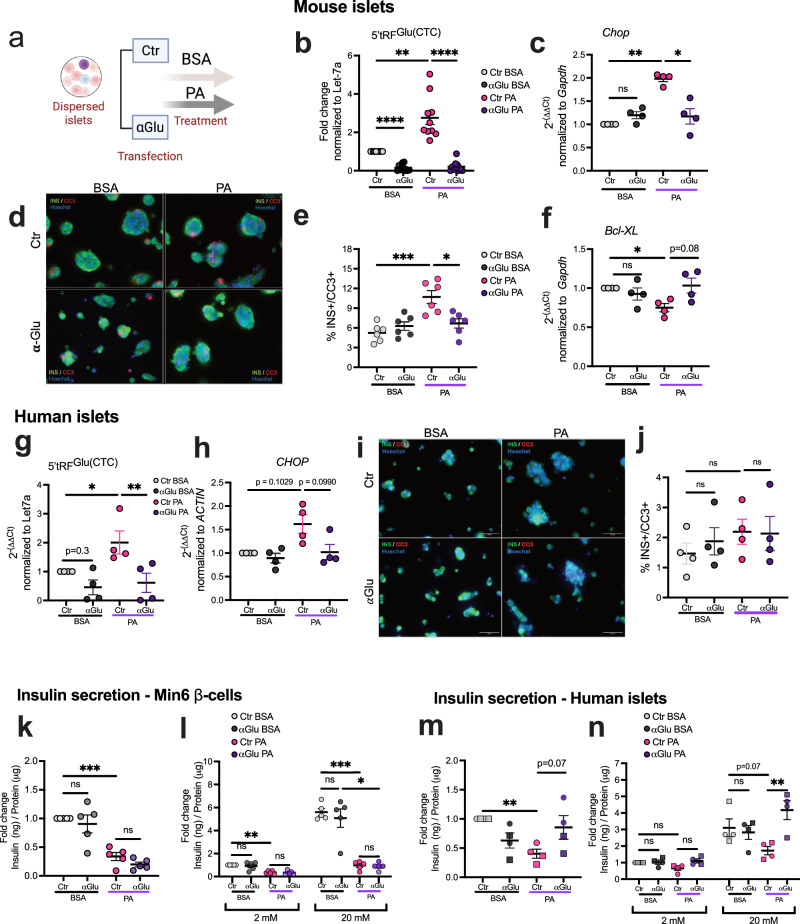
5’tRF^Glu(CTC)^ inhibition in islet cells protects against lipotoxicity. dispersed islet cells were transfected with an antisense oligonucleotide (ASO) targeting 5’tRF^Glu(CTC)^ (αGLU) or an antisense of control (Ctr) and were exposed to either vehicle (BSA) or palmitate (PA) for 48 h (**a**, Created in BioRender. Cosentino, C. (2026) https://BioRender.com/cih3hct). In mouse islets, the levels of 5’tRF^Glu(CTC)^ were assessed by qPCR and normalized by the geometric mean of Let7a and miR-7 expression (**b**, *n* = 10 independent experiments). *Chop* expression was normalized by the levels of *Gapdh* (**c**, *n* = 4 independent experiments). Insulin (green) and cleaved caspase (red) positive cells were detected by immunofluorescence (**d**) and the percentage of double positive over total insulin positive cells was calculated (**e**, *n* = 6 independent experiments). Bcl-XL expression was normalized by *Gapdh* levels (**f**, *n* = 4 independent experiments). In human islets, the levels of 5’tRF^Glu(CTC)^ were assessed by qPCR and normalized by Let7a expression and *CHOP* levels were corrected for *ACTIN* expression (**g**, **h**, *n* = 4 independent experiments). Insulin (green) and cleaved caspase (red) positive cells were detected by immunofluorescence (**i**), and the percentage of double positive over total insulin positive cells was calculated (**j**, *n* = 4 independent experiments). MIN6 (**k**, **l**) and human islets (**m**, **n**) cellular insulin content and released insulin were measured by ELISA in BSA or PA treatment. Glucose-induced insulin secretion was estimated in basal (2 mM glucose) and stimulating (20 mM glucose) conditions. Both intracellular and released insulin was normalized by total protein content. 5 independent experiments were quantified for MIN6, 2 experimental replicates from 2 independent human islet preparations were analysed (identified with different symbol shapes). Data in (**b**, **c**, **e**–**h**, **j**–**n**) are presented as mean values ± SEM, *p < 0.05, **p < 0.01, ***p < 0.001, ****p < 0.0001 by One-way ANOVA with Sidak multiple comparison correction (**b**–**j**, **k**, **m**) and by two-way ANOVA with Sidak multiple comparison correction (**l**, **n**). Exact *p* values in (**b**) αGLU vs Ctr BSA *p* = 0.0000003, Ctr PA vs Ctr BSA *p* = 0.0022, αGLU vs Ctr PA *p* = 0.00003; in (**c**) Ctr PA vs Ctr BSA *p* = 0.0016, αGLU vs Ctr PA *p* = 0.0410; in (**e**) Ctr PA vs Ctr BSA *p* = 0.0007, αGLU vs Ctr PA p = 0.0433; in (f) Ctr PA vs Ctr BSA *p* = 0.0365; in (**g**) Ctr PA vs Ctr BSA *p* = 0.0271, αGLU vs Ctr PA *p* = 0.0040; in (**k**) Ctr PA vs Ctr BSA *p* = 0.0001; in (l) in 2 mM glucose Ctr PA vs Ctr BSA p = 0.0015, in 20 mM glucose Ctr PA vs Ctr BSA *p* = 0.0005, αGLU PA vs αGLU BSA *p* = 0.0128; in (**m**) Ctr PA vs Ctr BSA *p* = 0.0089, in (**n**) in 20 mM glucose Ctr PA vs Ctr BSA *p* = 0.0027. Source data are provided as a Source Data file.

Next, we investigated the impact of a global reduction of 5’tRF^Glu(CTC)^ in islet cells on β-cell homeostasis. For this purpose, we used an antisense oligonucleotide (ASO)-based approach to target and inhibit 5’tRF^Glu(CTC)^ (*α-*Glu) under basal and PA conditions (Fig. 3a). Transfection of *α-*Glu ASO resulted in 70-90% reduction of 5’tRF^Glu(CTC)^ in mouse islet cells (Fig. 3b) and MIN6-β-cells (Figure S4a). Notably, *α-*Glu ASO did not affect full-length tRNA^Glu(CTC)^ levels or its aminoacylation (Figure S4b, c). We observed that the inhibition of 5’tRF^Glu(CTC)^ in mouse islet cells prevents PA-induced ER-stress, assessed by measuring the level of the stress-inducible transcription factor *Chop* (Fig. 3c), and protects β-cells from apoptosis measured by Caspase 3 cleavage (Fig. 3d, e). Previous studies have shown that PA activates the intrinsic apoptotic pathway in β-cells by modulating the levels of Bcl-2 homology 3 (BH3)-only proteins^25^. To investigate the protective mechanism elicited by 5’tRF^Glu(CTC)^ inhibition, we analyzed the expression of the pro-survival genes *Bcl-XL* (alias *Bcl2l1*) and *Bcl2* in mouse islets transfected with Ctr or *α-*Glu ASOs. In Ctr-transfected cells, PA treatment induced a downregulation of *Bcl-XL*, whereas inhibition of 5’tRF^Glu(CTC)^ preserved its expression (Fig. 3f). In contrast, *Bcl2* expression was not significantly altered under any of the experimental conditions (Figure S4d). Experiments performed in human islets confirmed that inhibition of 5’tRF^Glu(CTC)^ prevents PA-induced *CHOP* upregulation (Fig. 3g, h). However, human β-cell viability was not significantly affected by 48 h PA treatment (Fig. 3i, j) and *BCL-XL* expression levels remained unchanged (Figure S4e).

Next, we assessed the consequence of 5’tRF^Glu(CTC)^ blockade on insulin secretion. Chronic exposure to PA impairs insulin biosynthesis^26^ and inhibits glucose-stimulated insulin secretion (GSIS) by affecting the β-cell secretory machinery^27^. We measured insulin content and release of MIN6-β-cells and dispersed human islets transfected with either Ctr or *α-*Glu ASOs. As expected, PA treatment reduced both insulin content and GSIS in MIN6 cells and human islets (Fig. 3k–n). In MIN6 cells, 5’tRF^Glu(CTC)^ inhibition did not prevent the PA-induced loss of insulin content and secretion (Fig. 3k, l). In contrast, in human islets, insulin content was partially restored, and GSIS was significantly improved upon tRF inhibition (Fig. 3m, n). We further assessed residual β-cell secretory function by normalizing insulin secretion to total insulin content (Figure S4f, g). This parameter was significantly impaired by PA in MIN6 cells (Figure S4f), whereas it was preserved in human islets (Figure S4g). Notably, inhibition of 5’tRF^Glu(CTC)^ significantly improved residual β-cell secretory function in both models (Figure S4f, g).

### Blockade of 5’tRF^Glu(CTC)^ prevents the switch of macrophage phenotype induced by palmitate and ameliorates β-cell homeostasis

**Fig. 4:**
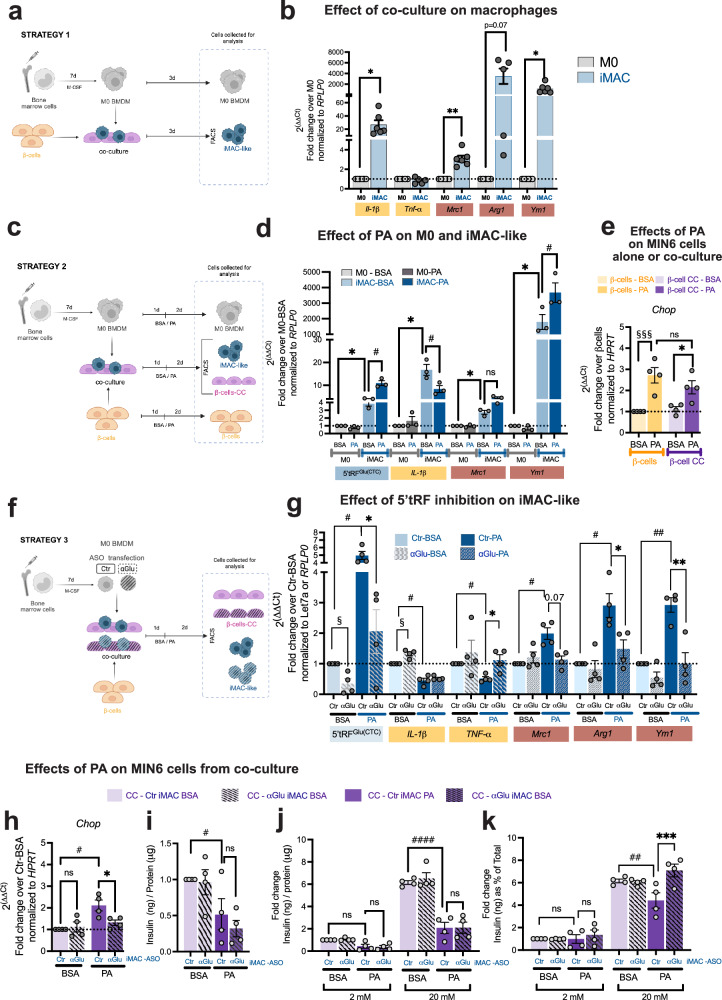
5’tRF^Glu(CTC)^ inhibition in iMAC-like cells attenuates the phenotypic switch induced by palmitate and protects co-cultured *β*-cells. schematic representation of strategy 1 co-culture: bone marrow-derived cells were differentiated for 7 days with M-CSF; naïve (M0) BMDMs were co-cultured with MIN6 *β*-cells for 3 days, and the two cell populations were separated by FACS (**a**). The expression of polarization markers in iMAC-like cells (blue bars) was compared with M0 M*φ*s cultured alone (gray bars) in parallel, and normalized to *Rplp0* (**b**, *n* = 6 independent experiments for *Il-1β*, *Tnf-α, Mrc1*, and *Ym1*, *n* = 5 independent experiments for *Arg1*); *p* values have been computed by two-sided paired Student *t* test for each gene, *p < 0.05, **p < 0.01 iMAC vs M0, exact *p* values: *Il-1β* *p* = 0.0118, *Mrc1* *p* = 0.0012, *Ym1* *p* = 0.0215. In strategy 2 (**c**) cells in co-culture for 24 h were treated with either BSA or PA for an additional 48 h. The levels of 5’tRF^Glu(CTC)^ and polarization markers were assessed in FACS-sorted iMAC-like cells treated with PA (dark blue bars) or BSA (light blue bars) and in M0 macrophages cultured alone in parallel and treated with PA (dark gray bars) or BSA (light gray bars) (**d**, *n* = 3 independent experiments); *p* values were computed by One-way ANOVA with Sidak correction for multiple comparisons.*p < 0.05 iMAC BSA vs M0 BSA, exact *p* values 5’tRF^Glu(CTC)^ *p* = 0.0221, *Il-1β* *p* = 0.0220, *Mrc1* *p* = 0.0401, *Ym1* *p* = 0.0465; #*p* < 0.05 iMAC PA vs BSA, exact *p* values 5’tRF^Glu(CTC)^ *p* = 0.0370, *Il-1β* p = 0.0212, *Ym1* *p* = 0.0122. MIN6 were collected by FACS from co-culture treated with PA (dark purple bars) or BSA (light purple bars) or from parallel single culture treated with PA (dark yellow bars) or BSA (light yello bars) and *Chop* was measured by qPCR (**e**, *n* = 4 independent experiments); §§§*p* < 0.001 *β*-cell PA vs BSA (*p* = 0.0006), *p < 0.05 *β*-cell-CC PA vs *β*-cell-CC BSA (*p* = 0.0132) by One-way ANOVA with Sidak correction for multiple comparisons. Schematic representation of co-culture strategy 3 in which M0 M*φ*s were previously transfected with ASO of Ctr or *α*GLU, and co-cultures were then treated with BSA or PA (**f**). 5’tRF^Glu(CTC)^ and polarization markers were measured in iMAC-like cells transfected with *α*GLU (pattern bars) or Ctr (full bars) in PA (dark blue) or BSA conditions (light blue) (**g**, *n* = 4 independent experiments); *p* values were computed by One-way ANOVA with Sidak correction for multiple comparisons, §*p* < 0.05 *α*GLU BSA vs Ctr BSA, exact *p* values: 5’tRF^Glu(CTC)^ *p* = 0.0296, *Il-1β* p = 0.0314; #*p* < 0.05, ##*p* < 0.01 Ctr PA vs Ctr BSA, 5’tRF^glu(CTC)^ *p* = 0.0147, *Il-1β* p = 0.0167, *Tnf-α* *p* = 0.0123, *Mrc1* p = 0.0276, *Arg1* *p* = 0.0318, *Ym1* *p* = 0.0078; *p < 0.05, **p < 0.01 *α*GLU PA vs Ctr PA, 5’tRF^glu(CTC)^ *p* = 0.0147, *Tnf-α* p = 0.0460, *Arg1* *p* = 0.0441, *Ym1* *p* = 0.0078. *Chop* expression (**h**), insulin content (**i**) and glucose-induced insulin secretion normalized with protein content (**j**) or expressed as percentage of total insulin (**k**) were assessed in MIN6 *β*-cells cultured with iMAC-like cells of Ctr (full bars) or transfected with *α*GLU (pattern bars) in BSA (light purple bars) or PA treatment (dark purple bars) conditions (*n* = 4 independent experiments). *P* values were computed by One-way ANOVA with Sidak correction for multiple comparisons, #*p* < 0.05, ##p < 0.01, ####p < 0.0001 comparing *β*-cells cultured with iMAC-Ctr in PA vs BSA, exact *p* values: **h**) *p* = 0.0432, **i**) *p* = 0.0234; **j**) in 20 mM glucose *p* = 0.000000003, **k**) in 20 mM glucose *p* = 0.0084; *p < 0.05, ***p < 0.001 comparing *β*-cells in PA cultured with iMAC- *α*GLU vs iMAC-Ctr: exact *p* values: **h**) *p* = 0.0258 **k**) in 20 mM glucose *p* = 0.0001. Data in (**b**, **d**, **e**, **g**–**k**) are presented as mean values ± SEM, source data are provided as a Source Data file. Schematic representations of co-culture strategies in (**a**, **c**, **f**) were created in BioRender. Cosentino, C. (2026) https://BioRender.com/btxni70.

In addition to endogenous processes disrupting β-cell homeostasis, the proliferation and activation of iMACs is expected to play a critical role in the development of diabetes under obesity conditions. We asked whether the modulation of 5’tRF^Glu(CTC)^ specifically in macrophages contributes to these pathogenic mechanisms. To this end, we aimed to target and inhibit 5’tRF^Glu(CTC)^ in iMACs and assess the resulting effects on both Mφ phenotype and β-cell homeostasis. However, manipulating primary iMACs poses significant challenges, as these cells, once isolated by FACS, rapidly lose their phenotype and are not viable when cultured alone. To overcome these limitations and since the activation state of iMACs is mainly determined by the crosstalk with surrounding endocrine cells, we established an in vitro model to explore these interactions under controlled conditions. To this end, bone marrow-derived cells from wild-type mice were differentiated in vitro into naïve (M0) Mφs (Figure S5a). M0 Mφs were further differentiated by culturing them in direct contact with MIN6 β-cells to induce an iMAC-like phenotype (Strategy 1, Fig. 4a). After 3 days of co-culture, the cells were separated by FACS (Figure S5b). The iMAC-like phenotype was evaluated by the expression of gene markers for Mφ polarization. Compared to M0 Mφs cultured in parallel for 3 days alone, iMAC-like cells showed an upregulation of the pro-inflammatory marker *IL-1β* and, at the same time, an induction of the anti-inflammatory markers *Mrc1, Arg1* and *Ym1* (Fig. 4b). This mixed phenotype with high *IL-1β* expression recapitulates previous observations on the activation state of iMACs in vivo^28^. We next evaluated the impact of PA treatment on iMAC-like cells in co-culture compared to M0 macrophages by adding the fatty acid to the co-culture media for 48 h prior to cell collection (Strategy 2, Fig. 4c). We observed that in iMAC-like cells 5’tRF^Glu(CTC)^ was higher compared to M0 and PA further induced the tRF levels (Fig. 4d). At the same time, PA increased the anti-inflammatory markers *Mrc1* and *Ym1*, while decreasing the pro-inflammatory cytokines *IL-1β* and *TNF-α* (Fig. 4d). These results matched the transcriptomic modulation observed in iMACs from mice fed with high-fat diet^3^ and from db/db mice^6^. The expression of the tRF and the polarization markers in M0 macrophages was not affected by PA (Fig. 4d). Finally, we assessed the impact of the modulation of 5’tRF^Glu(CTC)^ on iMAC-like cells. To this purpose, M0 Mφs were transfected with either Ctr or αGlu ASO prior to co-culture and then treated with BSA or PA (Strategy 3, Fig. 4f). In FACS-sorted iMAC-like cells, transfection with Ctr oligonucleotide PA raised 5’tRF^Glu(CTC)^ levels, while αGlu transfection led to a decrease of the tRF in both BSA and PA conditions (Fig. 4g). We confirmed that PA decreased *IL-1β* and *Tnf-α* expression in Ctr iMAC-like cells and upregulated the homeostatic genes *Mrc1, Arg1* and *Ym1* (Fig. 4g – full bars). At the same time, we observed that 5’tRF^Glu(CTC)^ inhibition led to an increase of *IL-1β* expression in basal conditions and prevented *Tnf-α* decrease and *Mrc1* and *Ym1* induction elicited by PA exposure (Fig. 4g – pattern bars). These data suggest that the 5’tRF^Glu(CTC)^ may be a key modulator of iMAC activation switch in response to environmental stimuli.

To assess whether the co-culture with Mφs modulates MIN6 β-cell responses to PA, we isolated the insulin-secreting cells by FACS and measured the expression of *Chop*, which is induced by ER-stress during lipotoxicity^29^. We found that co-culture with Mφs did not significantly affect the modulation of this gene (Strategy 2, Fig. 4e). However, co-culture with αGlu iMACs - lacking 5’tRF^Glu(CTC)^ – prevented *Chop* induction during PA treatment (Strategy 3, Fig. 4h). While PA still decreased insulin content and secretion in MIN6 cells co-cultured with Ctr Mφs, the fraction of insulin content released in response to glucose was restored by co-culture with iMACs depleted from 5’tRF^Glu(CTC)^ (Fig. 4i–k).

Taken together, our observations suggest that targeting 5’tRF^Glu(CTC)^ in Mφs may have indirect beneficial effects on β-cells, probably due to the attenuation of the phenotypic switch induced by saturated fatty-acid exposure.

### 5’tRF^Glu(CTC)^ interacts with RNA-binding proteins and participates in gene expression regulation

**Fig. 5:**
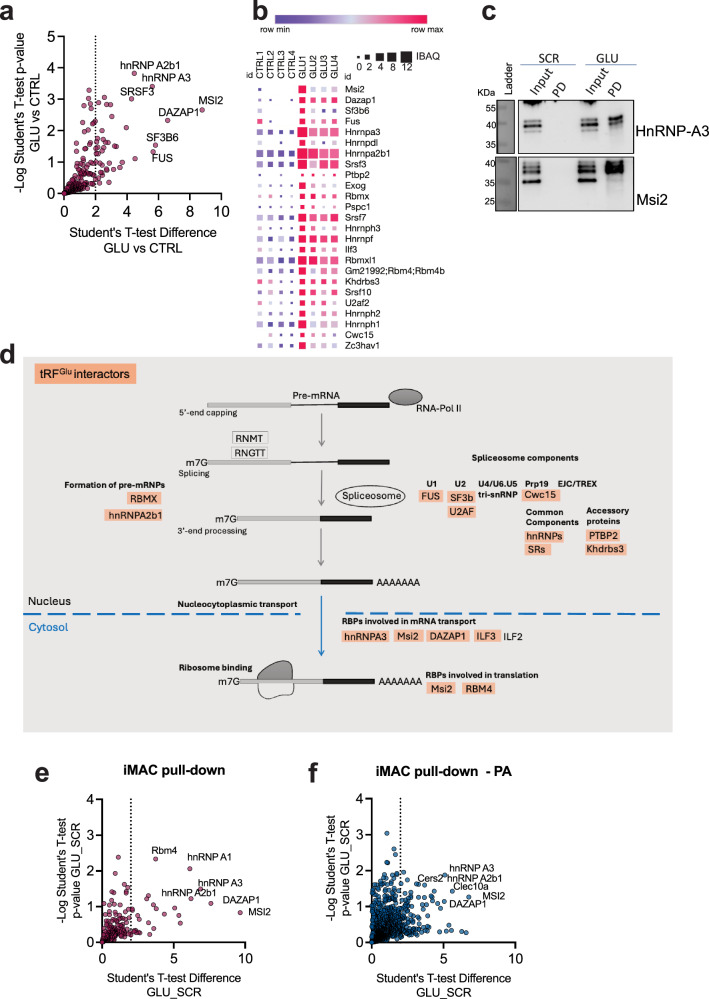
Identification of 5’tRF^Glu(CTC)^ interactors. MIN6 cells transfected with a biotinylated 5’tRF^Glu(CTC)^ mimic (GLU) or a scrambled control (CTRL) were used for pull-down and mass spectrometry (*n* = 4 independent experiments). Volcano plot shows the proteins enriched in the GLU pull-down versus CTRL (**a**). Heatmap shows the 25 proteins selected as possible interactors (enrichment FC > 2 GLU mimic vs CTRL, unique peptides>2, *p* < 0.05), the square size indicates the IBAQ values (0 to 14) (**b**). Western blot of hnRNP-A3 and MSI2 validating the mass spectrometry data (**c**, results were reproduced 2 times). The scheme represents the involvement of 5’tRF^Glu(CTC)^ interactors in the different steps of mRNA processing pathway (**d**). iMAC-like cells were differentiated in co-culture with β-cells and treated with either BSA or PA (**e**–**f**). iMAC lysates were incubated with GLU or CTRL mimics and used for pull-down and mass spectrometry (*n* = 2 independent experiments).

We then explored the mechanism by which 5’tRF^Glu(CTC)^ exerts its regulatory effect in islet cells. tRFs can mediate a variety of cellular functions depending on the molecular interactions in which they are engaged. Therefore, we decided to identify the protein interactors of 5’tRF^Glu(CTC)^. A biotinylated tRF mimic (GLU) or a scrambled control (CTRL) was transfected in MIN6 cells (Figure S6a); following UV-crosslink, cell lysates were pulled-down and analysed by mass spectrometry (Fig. 5a). Notably, transfection of the GLU mimic did not alter the expression of the β-cell maturation markers *Ins* and *Pdx1*, and did not induce ER-stress as assessed by *Chop* expression (Figure S6b–d) This technique allowed the identification of 25 proteins likely interacting with 5’tRF^Glu(CTC)^ (Fig. 5b). The interactions with Msi2, the most enriched protein, and with hnRNP-A3, the most abundant protein in the 5’tRF^Glu(CTC)^ pull-down, were validated by western blot (Fig. 5c). Functional enrichment revealed an overrepresentation of GO terms RNA-splicing and RNA-processing in biological processes and spliceosome complex, ribonucleoprotein granules and nuclear speckles in cellular components (Figure S6f, g). Indeed, 5’tRF^Glu(CTC)^ interactors consist of RNA-binding proteins (RBPs) involved in several steps of gene expression regulation, such as splicing, transport and translation (Fig. 5d). To further characterize the connectivity among these RBPs, we performed STRING-based protein-protein interaction analysis, which revealed that the 5’tRF^Glu(CTC)^ interactors form a highly interconnected network (Figure S6h). Interestingly, RBPs and mRNA processing play a crucial role in β-cell homeostasis^30,31^. Moreover, the RBP Msi2, detected as 5’tRF^Glu(CTC)^ interactor, was shown to be overexpressed in immature β-cells and to be induced in islets in conditions associated with T2D, such as ER-stress and lipotoxicity, controlling mRNA translation^32^.

We then investigated whether the interactions identified in β-cells were conserved in iMACs. To this purpose, macrophages differentiated into iMAC-like cells by co-culture with β-cells were subjected to pull-down assays using biotinylated GLU or CTRL mimics. Mass spectrometry analysis identified 28 proteins interacting with 5’tRF^Glu(CTC)^ in iMACs (Fig. 5e). Among these, 11 were RBPs also detected in β-cells, including the most highly enriched RBPs: Msi2, hnRNP-A3, hnRNP-A2b1, Dazap1, and hnRNP-Ab. We further exposed iMAC-like cells to PA for 48 h prior to the pull-down experiments. Under lipotoxic conditions, the most enriched interactors were RBPs commonly identified in both β-cells and iMACs under basal conditions. PA-treated iMAC-like cells displayed an increased number of proteins potentially interacting with 5’tRF^Glu(CTC)^ (Fig. 5f). Newly identified putative interactors included components associated with nucleosomes and multivesicular bodies, as revealed by enrichment analysis (Figure S6i).

**Fig. 6:**
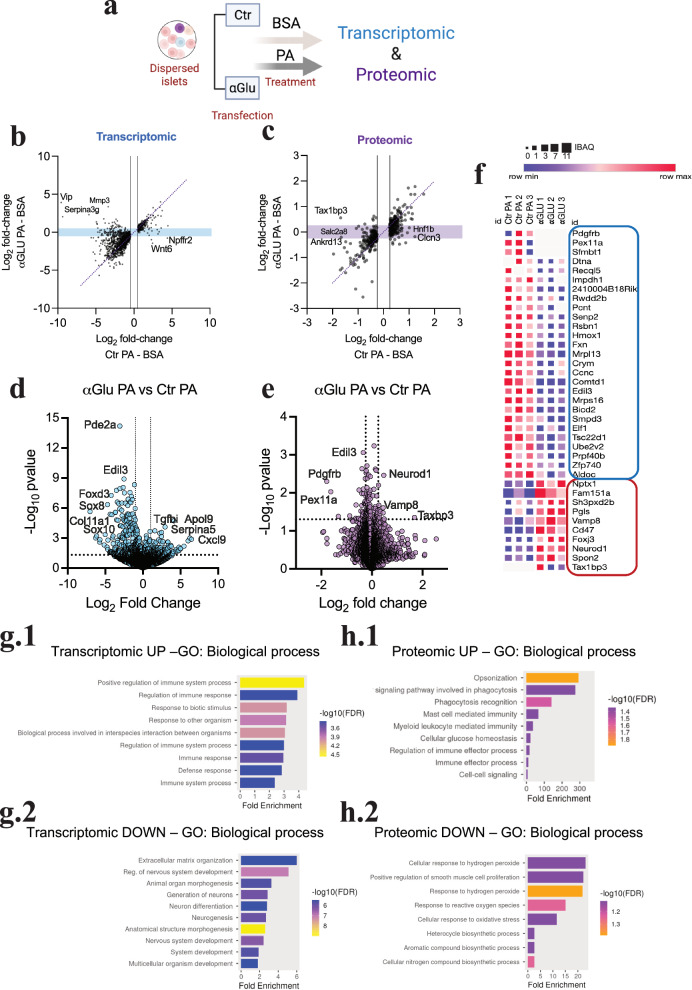
Analysis of gene regulation mechanisms affected by 5’tRF^Glu(CTC)^ inhibition. Primary mouse islets cells were transfected with antisense oligonucleotide (ASO) of control (Ctr) or inhibiting 5’tRF^Glu(CTC)^ (*α*-Glu) and then treated with either BSA or palmitate (PA), gene and protein expression were assessed by transcriptomic (*n* = 6 independent experiments) and proteomic analysis (*n* = 3 independent experiments) (**a**, Created in BioRender. Cosentino, C. (2026) https://BioRender.com/cih3hct). Differential expression of genes and proteins was evaluated by comparing PA to BSA. Fold-Change correlation analysis was performed to analyse the variation between PA-induced changes in Ctr cells (x axes) and *α*-Glu-transfected cells (**b**, **c**). Deviation from the identity line indicates different fold change and in some cases, direction of gene (**b**) and protein (**c**) modulation when the tRF is not present. Volcano plots showing differentially expressed genes (**d**) and proteins (**e**) in dispersed mouse islets due to 5’tRF^Glu(CTC)^ inhibition in palmitate treatment condition (αGlu PA vs Ctr PA). The proteins significantly modulated (*p* < 0.05) by the tRF inhibition in palmitic condition are shown in the heatmap (**f**). Differential expression was computed using a negative binomial generalized linear model with two-sided *t*-test and Benjamini–Hochberg FDR correction. Overrepresented GO biological process terms in up- and down- regulated genes (**g1-2**) and proteins (**h1-2**).

Because of the identified interactions with RBPs conserved in both β-cells and iMAC-like, we further investigated the consequences of 5’tRF^Glu(CTC)^ modulation in transcriptional and post-transcriptional gene regulation. To this purpose, we conducted transcriptomic and proteomic analysis in mouse islet cells upon transfection with either Ctr or αGlu ASO (Fig. 6a).

mRNA sequencing identified 1534 genes modulated in mouse islets in response to PA treatment (Ctr PA vs Ctr BSA, FC > 2, *p* < 0.05) (Figure S7a), with an upregulation of fatty-acid metabolism components, and a downregulation of genes involved in haematopoiesis and cell adhesion (Figure S7.b1-4). Although the overall effects on gene expression modulation were maintained, several PA-responsive genes showed attenuated or opposite fold changes in αGlu-transfected cells, resulting in deviations from the identity line in the fold-change correlation analysis (Fig. 6b). By comparing of αGlu versus Ctr-transfected cells, we detected 434 differentially expressed genes (FC > 2, *p* < 0.05) in BSA (Figure S7c) and 400 in PA-treated cells (Fig. 6d). Interestingly, in both BSA and PA conditions, inhibition of 5’tRF^Glu(CTC)^ led to an upregulation of genes involved in monocyte attraction and in immune responses (Figs. 6g1 and S7.d1). Functional analysis of the downregulated genes in BSA condition revealed a significant enrichment of pathways related to antigen presentation and adaptive immune responses (Figure S7.d2). In PA condition, we observed a downregulation of genes involved in enteric nervous system development and extracellular matrix organization (Fig. 6g2). Although these genes are not directly linked to β-cell homeostasis, several of the modulated transcripts (e.g., *Gdnf, Ngfr, S100b, Hand2, Foxd3, Mrgprf, Cadm3, Gap43, Gpm6b, Cd109, Mmp17, Chst3, Gfap*) are associated with neurotrophic signaling and cell interaction pathways, indicating a potential role in the modulation of islet microenvironment.

Because of the apparent link between 5’tRF^Glu(CTC)^ and mRNA processing and translation, we performed a proteomic characterization of mouse islet cells upon PA exposure and 5’tRF^Glu(CTC)^ inhibition. Proteomic analysis identified 8359 proteins, 218 of which were modulated by PA treatment (Ctr PA vs Ctr BSA, FC > 1.25, *p* < 0.05) (Figure S8a). We observed an upregulation of proteins implicated in the transport to peroxisome and fatty-acid degradation and a downregulation of proteins involved in extracellular matrix composition and metabolic responses to glucose (Figure S8.b1-2), in accordance with the transcriptomic data. Fold-change correlation analysis revealed a partial attenuation, and in some cases reversal, of PA-induced protein expression changes observed in Ctr cells upon αGlu transfection, although the global protein modulation was conserved (Fig. 6c). We identified 82 differentially expressed proteins in *α-*Glu versus Ctr ASO in basal conditions (*α-*Glu BSA vs Ctr BSA, FC > 1.25, *p* < 0.05) (Figure S8c) and 26 in PA exposure condition (*α-*Glu PA vs Ctr PA FC > 1.25, *p* < 0.05) (Fig. 6e). In agreement with the transcriptomic analysis, tRF inhibition led to the upregulation of proteins related to phagocytosis and innate immunity, in both BSA and PA conditions (Fig. 6h1 and S8.d1). The tRF blockade in basal conditions led to the downregulation of pathways linked to cytochrome c oxidase activity and protein complex assembly (Figure S8.d2). Upon PA treatment, tRF blockade resulted in the downregulation of proteins involved in oxidative stress responses (Fig. 6h2). Notably, we found that some proteins modulated by PA exposure were maintained at their physiological levels in 5’tRF^Glu(CTC)^-depleted islet cells (Figure S8.e1-4). For instance, the drastic drop caused by PA treatment of Tax1bp3, a repressor of Wnt/β-catenin pathway, is completely prevented in cells lacking 5’tRF^Glu(CTC)^. Similarly, the downregulation of NeuroD1 and VAMP8, important for β-cell differentiation^33^ and insulin secretion^34^, respectively, occurring in PA-treated cells, is no longer observed upon blockade of 5’tRF^Glu(CTC)^.

Altogether, our functional investigations suggest that 5’tRF^Glu(CTC)^ contributes not only to endogenous β-cell processes, by regulating the levels of a subset of proteins involved in oxidative stress and insulin secretion, but also to islet dynamics, by controlling the expression of genes and proteins involved in innate immunity activation.

It has been previously shown that in vitro exposure to PA and high glucose concentrations can partially recapitulate the transcriptomic changes observed in human islets from individuals with T2D^35^. To investigate whether gene expression changes mediated by 5’tRF^Glu(CTC)^ are linked to human pathophysiology, we leveraged bulk RNA-sequencing and proteomic datasets from the humanislet.com platform^36^. By comparing the transcriptomic changes in T2D versus non-diabetic (ND) human islets with those of PA- versus BSA-treated mouse islets, we identified 63 commonly upregulated and 39 commonly down-regulated genes meeting fold-change and statistical significance thresholds (Log2FC > 1, *p*adj < 0.1, Figure S9a, b). The commonly upregulated genes were predominantly associated with metabolic and biosynthetic pathways as well as apoptotic processes, whereas down-regulated genes were enriched for pathways involved in cell signaling and communication (Figure S9c, d). Correlation analysis of fold changes revealed that only a subset of these transcriptomic alterations was reversed or attenuated upon inhibition of 5’tRF^Glu(CTC)^ (Figure S9e). Comparative analysis of proteomic datasets revealed 37 upregulated and 17 down-regulated proteins commonly modulated in T2D human islets and PA-treated mouse islets (Figure S9f, g). Upregulated proteins were mainly involved in metabolic processes and fatty-acid metabolism, insulin signaling and ribosome biogenesis (Figure S9h), whereas down-regulated proteins were enriched in pathways related to neurodegeneration, cell projection and protein secretion (Figure S9i). Notably, inhibition of 5’tRF^Glu(CTC)^ attenuated or reversed many of the proteomic alterations shared with T2D condition (Figure S9j, k).

Collectively, these findings suggest that 5’tRF^Glu(CTC)^ is more closely associated with proteomic rather than transcriptomic changes found in human T2D pathophysiology.

### 5’tRF^Glu(CTC)^ modulation contributes to changes in protein translation efficiency in response to lipotoxic stress

**Fig. 7:**
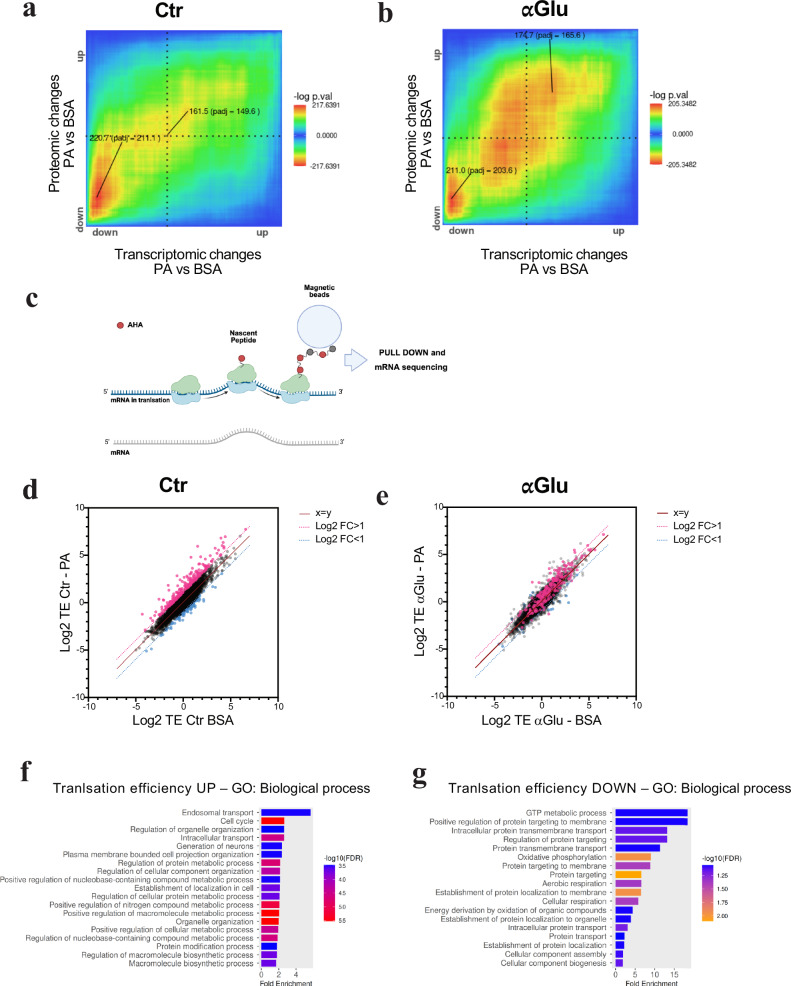
Effect of 5’tRF^Glu(CTC)^ inhibition on translational regulation. Rank-rank hypergeometric overlap of the modulation in proteome and transcriptome was performed with RedRibbon (**a**, **b**). **a** Show RRHO of Ctr mouse islet cells treated with PA, while (**b**) shows RRHO in αGlu-transfected cells. c) Schematic representation of the experimental strategy for translatome characterization performed in *n* = 3 independent experiments (Created in BioRender. Cosentino, C. (2026) https://BioRender.com/t8wzrn9). Translation efficiency correlation plots (**d**, **e**) are obtained by plotting Log2 TE in BSA in x and in PA in y. TE correlation plots are shown for Ctr cells (**d**) and for αGlu transfected cells (**e**). Bar plots showing the GO:biological process terms enriched for transcripts with increased TE (**f**) and with decreased TE (**g**) in Ctr cells.

We then used a rank–rank hypergeometric overlap (RRHO)-based method to investigate the coupling between proteomic and transcriptomic regulation^37^. The proteomic and transcriptomic changes induced by PA treatment showed an overall overlap (Fig. 7a), which was more pronounced in the down-down RRHO quadrant, consistent with previous observations in β-cell lines^38^. In contrast, the weaker overlap observed in the up-up quadrant suggests that PA elicits additional post-transcriptional mechanisms. Notably, upon inhibition of 5’tRF^Glu(CTC)^ (*α-*Glu), PA induced a more concordant modulation of transcripts and proteins, as indicated by increased RRHO significance (Fig. 7b). These findings support a role for this tRF in post-transcriptional regulation.

Based on these mouse islet omics data, we next examined the impact of 5’tRF^Glu(CTC)^ modulation on protein synthesis. To assess both global and transcript-specific changes in translation, we employed a translatomic approach that isolates mRNAs associated with actively translating ribosomes (Fig. 7c). Ribosome-bound mRNAs were quantified by RNA sequencing and compared with total mRNA levels from input lysates. Translation efficiency (TE) was calculated as the ratio between ribosome-associated and total mRNA abundance. MIN6-β-cells were transfected with either Ctr or αGlu ASOs and subjected to translatomic profiling. Correlation analysis of TE values in Ctr-transfected cells treated with PA or BSA revealed PA-dependent alterations in translation efficiency (Dataset 1, Fig. 7d). This revealed PA-dependent changes in TE. Specifically, PA predominantly increased the TE of a subset of mRNAs (pink dots in TE-correlation plot, Log_2_ TE Fold Change > 1), while only a small number of transcripts exhibited reduced TE (blue dots, Log_2_ TE Fold Change < 1). Notably, in αGlu-transfected cells, PA treatment failed to modulate the TE of these transcripts (pink and blue dots, Fig. 7e). Enrichment analysis revealed that PA increases the translation of transcripts involved in endocytosis, cell cycle and intracellular and cell projection organization, as well as metabolic processes, while decreasing the TE of components of oxidative phosphorylation and cellular respiration (Fig. 7f, g)

Finally, we examined the direct effects of 5’tRF^Glu(CTC)^ inhibition by performing RRHO analysis comparing αGlu vs Ctr under both BSA and PA conditions. In this case, transcriptomic and proteomic changes showed a lower degree of overlap (Figure S10a, b), consistent with a fine-tuning rather than a global reprogramming of gene expression. The up-up quadrant confirmed an enrichment of pathways related to innate immunity activation (Figure S10.c1), whereas the down-down quadrant was associated with vascular and nervous system modulation (Figure S10.c2). We also identified a set of genes displaying discordant regulation at mRNA and protein levels (Table S4). While their transcripts were upregulated or unchanged, the corresponding proteins were downregulated upon 5’tRF^Glu(CTC)^ inhibition. These proteins are primarily involved in mitochondrial translation and stress response pathways (GO: biological processes) (Figure S10.c3).

Collectively, these findings indicate that 5’tRF^Glu(CTC)^ plays a critical role in the fine-tuning of β-cell translational regulation under lipotoxic conditions.

### 5’tRF^Glu(CTC)^ modulates macrophage gene expression and polarization

**Fig. 8:**
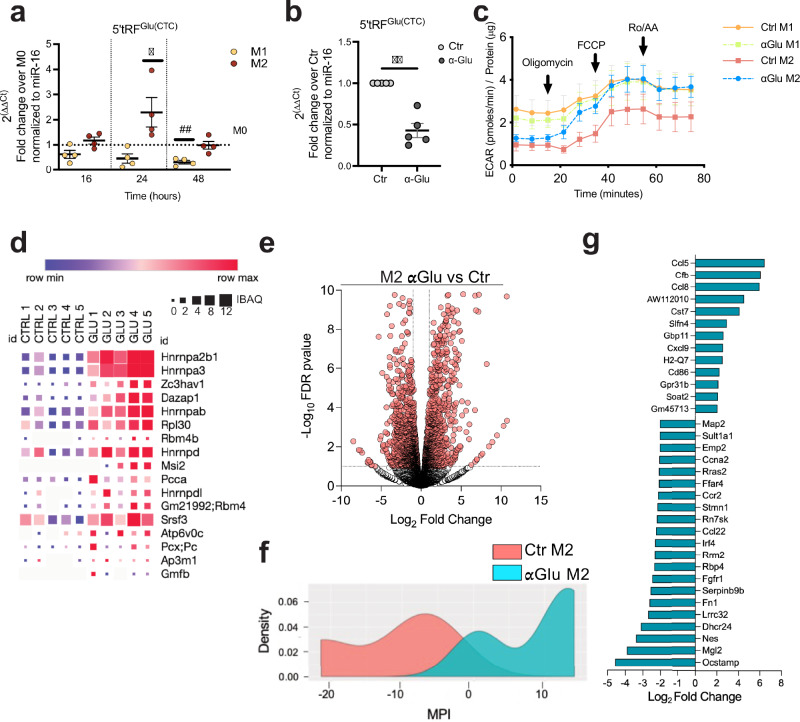
5’tRF^Glu(CTC)^ is necessary for M2 anti-inflammatory macrophage polarization. 5’tRF^Glu(CTC)^ levels were assessed by qPCR at the indicated time points of BMDM polarization into anti-inflammatory M2 (red dots) or pro-inflammatory (yellow dots) macrophages (**a**, *n* = 4 independent experiments) *p < 0.05 M2 vs M0 (*p* = 0.0383), ##p < 0.05 M1 vs M0 (*p* = 0.0075) by One-way ANOVA with Sidak correction for multiple comparisons. Transfection of naïve M0 macrophages with antisense oligonucleotide (ASO) targeting 5’tRF^Glu(CTC)^ (αGlu, dark gray dots) or antisense of control (Ctr, light gray dots) was performed prior BMDM polarization (**b**, *n* = 5, independent experiments, **p < 0.01 αGlu vs Ctr, *p* = 0.0025, by two-sided paired Student *t* test). The extracellular acidification rate of M1 or M2 macrophages, transfected with either αGlu or Ctr antisense was assessed with Seahorse analyser during a mito-stress assay (**c**, *n* = 4, independent experiments). Data in a-c are presented as mean values ± SEM. Source data are provided as a Source Data file. Heatmap show the proteins identified as 5’tRF^Glu(CTC)^ interactors in M2 macrophages by mass spectrometry (enrichment FC > 2 GLU mimic vs CTRL, unique peptides > 2, *p* < 0.05), the square size indicates the IBAQ values (0–12) (**d**, *n* = 5, independent experiments). iBAQ values were log2-transformed, filtered, and analyzed via two-sided Student’s *t* test with Benjamini–Hochberg correction (FDR < 0.05). Transcriptomic profiling of M2 macrophages upon 5’tRF^Glu(CTC)^ inhibition: volcano plot shows the differential expression of genes compared to M2 transfected with Ctr ASO (**e**, *n* = 3, independent experiments). Differential expression was computed using a negative binomial generalized linear model with two-sided tests and Benjamini–Hochberg FDR correction. MacSpectrum tool was used to evaluate the macrophage polarization state based on their transcriptomic profile (**f**) and to identify the key genes involved in the polarization shift towards a pro-inflammatory phenotype observed in M2 αGlu compared to Ctr (**g**).

As the blockade of 5’tRF^Glu(CTC)^ in mouse islet cells leads to the upregulation of genes and proteins involved in innate immune activation, we decided to further characterize the role of 5’tRF^Glu(CTC)^ in Mφs polarization. We used bone marrow-derived Mφs polarized in vitro into M1 pro- and M2 anti-inflammatory phenotypes, as assessed by polarization marker expression (Figure S11.a1-5). We found that 5’tRF^Glu(CTC)^ levels peak 24 h after the induction of anti-inflammatory phenotype by IL-4 and IL-13 and progressively decline thereafter during pro-inflammatory polarization induced by LPS and IFN-γ (Fig. 8a). Interestingly, Angiogenin expression is inhibited in early pro-inflammatory polarization (Figure S11b).

We next investigated whether 5’tRF^Glu(CTC)^ contributes to the establishment of an anti-inflammatory Mφ phenotype. To this end, we inhibited the expression of this fragment in M0 Mφs using an antisense oligonucleotide (α-Glu ASO) prior to polarization (Fig. 8b). To evaluate Mφ metabolic activity, we measured oxygen consumption rates (OCR) and extracellular acidification rates (ECAR) using the Seahorse Mito Stress Test (Agilent). OCR serves as a readout of mitochondrial respiration, which is typically elevated in anti-inflammatory Mφs, whereas ECAR provides an indirect measure of glycolysis, the dominant energy pathway in pro-inflammatory Mφs^39^. We found that blockade of 5’tRF^Glu(CTC)^ led to higher ECAR in anti-inflammatory Mφs (Fig. 8c). At the same time, the OCR measurements showed an increased response to FCCP (Figure S11c), indicating higher maximal respiratory capacity. This may be due to metabolic reprogramming^39^. Using a biotinylated mimic of 5’tRF^Glu(CTC)^, we pulled down the proteins interacting with the fragment in anti-inflammatory macrophages (Fig. 8d and Figure S11d, e). We found 17 proteins enriched in the pull-down performed with the tRF mimic (GLU), 8 of which were common to those pulled down in β-cells. As in β-cells, 5’tRF^Glu(CTC)^ interactors were mainly RBPs involved in mRNA splicing, processing and translation (Figure S11.f1-2). Therefore, we investigated whether 5’tRF^Glu(CTC)^ modulates gene expression in Mφs. mRNA sequencing of M2 anti-inflammatory Mφs revealed that 5’tRF^Glu(CTC)^ inhibition causes major transcriptional changes (Fig. 8e) with the downregulation of genes involved in cell division and lipid metabolism and an upregulation of genes related to inflammatory pathways (Figure S11.g1-2). Using MacSpectrum, a tool that predicts Mφ phenotype based on transcriptomic data^40^ we found that 5’tRF^Glu(CTC)^ inhibition triggers a shift towards higher and more pro-inflammatory Mφ polarization index (MPI) (Fig. 8f) because of changes in the expression of several key regulatory genes (Fig. 8g). Our observations point to a central role of tRFs in determining Mφ activation via regulation of gene expression.

## Discussion

In this study, we investigated the role of tRF modulation in pancreatic islets during the development of T2D. Our findings demonstrate that tRFs are dynamically modulated in vivo in islets from an obese and diabetic mouse model as well as from individuals with IGT and T2D; we showed that 5’tRF biogenesis is induced in vitro by free fatty-acid exposure and identified 5’tRF^Glu(CTC)^ as a key regulator of islet gene expression, stress responses and macrophage activation.

### Modulation of tRF cleavage in islet homeostasis and disease

Recent studies have highlighted the role of tRFs as critical regulatory molecules in islet biology. tRNA cleavage was initially implicated in early-onset diabetes in patients carrying mutations in the tRNA-modifying enzyme TRMT10A^16^. Our previous work showed that tRFs are physiologically expressed in rodent islets and are modulated during postnatal β-cell maturation^18^ and in response to nutritional changes^19^. In conditions such as obesity and IGT, exposure to elevated glucose and circulating free fatty acids triggers sustained cellular stress, contributing to β-cell dysfunction and apoptosis. This stress is accompanied by the expansion and activation of islet macrophages, which monitor and modulate β-cell fitness.

Despite these insights, the functional relevance of tRFs in islet homeostasis, their modulation in human T2D and their role in islet cell crosstalk remained largely unexplored. Using small RNA sequencing, we found an upregulation of 5’tRFs derived from tRNA^Gly(GCC)^ and tRNA^Glu(CTC)^ in islets from T2D patients and in both iMACs and β-cells from db/db mice. In less severe mouse models of obesity, such as ob/ob and diet-induced obesity, we did not observe tRF induction. In these models, glucose homeostasis is maintained, probably thanks to β-cell compensatory mechanisms. Notably, 5’tRF^Glu(CTC)^ was also upregulated in islets from individuals with IGT, with levels inversely correlating with insulin secretion and glucose sensitivity. These findings suggest that early induction of this tRF may contribute to diabetes onset. Interestingly, 5’tRF^Glu(CTC)^ is abundant in neonatal rat islets but downregulated during maturation^18^, suggesting that low levels of this fragment may be required to sustain mature β-cell function. Supporting this, downregulation of 5’tRF^Glu(CTC)^ in islet cells preserved residual glucose-induced insulin secretion following exposure to saturated fatty acids. We further demonstrated that this induction observed in vivo can be mimicked ex vivo by exposing islet cells to saturated fatty acids, especially palmitate. We found that 5’tRF induction is specifically associated with chronic lipotoxic stress, as acute fatty-acid exposure does not alter their levels. The ability of islet cells to recover from metabolic stress has been previously described^35^. Here, we showed that upon removal of the environmental stress, 5’tRF levels return to basal conditions in parallel with the resolution of ER-stress responses. These findings support the hypothesis that 5’tRFs act as stress-responsive regulators rather than being mere by-products of cellular damage.

While certain tRFs (i.e. 5’tRNA-halves) are produced in response to stress and may mediate stress signaling, other tRFs appear to fulfil homeostatic roles. We have recently demonstrated that mitochondrial-derived tRFs are essential for β-cell function^19^. In β-cell sorted from db/db mice, we observe a marked downregulation of 3’tRFs. Further studies are needed to determine whether these molecules constitute a class of mediators involved in the maintenance of β-cell homeostasis.

The generation of tRFs under physiological conditions and their induction under stress is driven by differential expression of tRNA genes^41^, or of tRNA-cleaving enzymes^10,24,42^. We found that palmitate does not alter the level or the aminoacylation of tRNA^Glu(CTC)^ but is likely to promote its cleavage by increasing the expression of the endoribonuclease Angiogenin, which is also upregulated in FACS-sorted β-cells from db/db mice. This is further supported by the coordinated restoration of 5’tRF and Angiogenin levels upon environmental stress removal. However, the induction of 5’tRF^Gly(GCC)^ was not prevented by Angiogenin knockdown, suggesting either incomplete silencing, activation via post-translational modifications, such as phosphorylation and translocation^43,44^, or the involvement of alternative RNAses.

Although the mechanisms underlying Angiogenin upregulation in obesity and lipotoxic conditions remain incompletely understood, activation of IRE1α–XBP1 arm of the unfolded protein response has been associated with Ang induction in renal epithelial cells^45^. In addition, inflammatory signaling pathways have been implicated in Ang upregulation in macrophages^46^. Elucidating the relative contributions and temporal interplay of these pathways in pancreatic islets will be an important focus of future investigations.

### tRF function in islet stress responses and iMAC activation

T2D typically arises from a pre-diabetic state characterized by impaired glucose tolerance (IGT) and mild hyperglycemia, with obesity being a key risk factor. Chronic nutrient excess initially triggers compensatory β-cell mass expansion and the activation of stress responses, but eventually leads to β-cell failure. We found that inhibition of 5’tRF^Glu(CTC)^ in dispersed islet cells averts palmitate-induced ER-stress and the activation of the intrinsic apoptotic pathway by preserving the expression of the pro-survival factor Bcl-XL. Prolonged palmitate exposure impaired insulin production and secretion in both MIN6-β-cells and human islets. However, human islets partially retained their glucose responsiveness and their secretory capacity, as evidenced by the maintenance of the fraction of insulin content released upon glucose stimulation. In contrast, the secretory capacity of MIN6 cells was markedly reduced under lipotoxic conditions. Targeting 5’tRF^Glu(CTC)^ partially restored insulin content and secretion in human islets, but not in MIN6 cells. Nevertheless, the inhibition of this tRF improved the residual capacity of β-cells to release insulin in response to glucose in both models, suggesting a role for 5’tRF^Glu(CTC)^ in stress-induced β-cell dysfunction.

Obesity and T2D are also characterized by chronic low-grade inflammation. This has been extensively studied in adipose tissue, where macrophages recruited from the circulation adopt a pro-inflammatory phenotype and release cytokines and extracellular vesicles that can affect islet function^47,48^. In physiological conditions, islet macrophages exhibit an activation state characterized by the production of pro-inflammatory cytokines^28^ but also by the expression of homeostatic genes^3^. In obesity, these cells undergo transcriptional reprogramming toward a more homeostatic phenotype marked by decreased IL-1β and increased expression of anti-inflammatory genes^3,6^.

Using a co-culture system, we modeled the activation dynamics of iMACs observed in mouse models of T2D. Bone marrow-derived macrophages co-cultured with MIN6 β-cells acquired an iMAC-like phenotype and, when exposed to palmitate, transitioned to an obesity-associated activation state. This indicates that β-cells influence macrophage polarization and that macrophage precursors can acquire iMAC characteristics under appropriate stimuli. While embryonic origin may be required for the acquisition of a fully specialized phenotype, precursor-derived macrophages may contribute to the maintenance of the iMAC pool. Our co-culture system enables studies on iMAC-like cell functions and on their crosstalk with β-cells. Indeed, we demonstrated that blockade of tRF^Glu(CTC)^ in macrophages prevents palmitate-dependent activation and protects β-cells in co-culture. Based on previous evidence^1^, our findings support a model in which 5′tRF^Glu(CTC)^ acts as a key mediator of β-cell–macrophage crosstalk under lipotoxic stress, promoting a macrophage polarization state that sustains β-cell stress and whose blockade preserves macrophage identity and improves β-cell homeostasis. The key role of 5′tRF^Glu(CTC)^ in macrophage polarization was corroborated by observations in polarized bone marrow-derived macrophages, where blockade of this tRF in anti-inflammatory macrophages led to a shift to a hypermetabolic, pro-inflammatory phenotype.

### Molecular mechanisms of tRF action

Gene regulation under stress involves the rapid and coordinated activity of multiple regulatory molecules. Small non-coding RNAs are rapidly modulated by environmental cues, and their interaction with RBPs can reshape both transcriptomic and proteomic landscapes. We identified a network of RBPs interacting with tRF^Glu(CTC)^, many of which are involved in mRNA processing and known to play roles in islet homeostasis and stress adaptation^31,32^. Consistent with these interactions, blockade of tRF^Glu(CTC)^ in islet cells altered gene and protein expression under both basal and lipotoxic conditions. Upon palmitate exposure, inhibition of tRF^Glu(CTC)^ led to upregulation of immune activation pathways, suggesting that this tRF normally acts to mitigate inflammatory responses under diabetogenic conditions by promoting a more homeostatic iMAC state. Additionally, depletion of tRF^Glu(CTC)^ downregulated genes involved in extracellular matrix remodeling and neurogenesis, processes likely to be important in reshaping the islet microenvironment during lipotoxicity^49,50^.

Proteomic analysis revealed that tRF^Glu(CTC)^ inhibition reduced the expression of proteins involved in oxidative stress responses, suggesting that these effects may underlie the protective effects observed in β-cells, since induction of oxidative stress is considered a major contributor to islet cell demise during lipotoxicity^29^.

Although some pathways were consistently modulated at both mRNA and protein levels, others showed discordant patterns, likely reflecting post-transcriptional control. Rank-rank hypergeometric overlap analysis confirmed that palmitate induced overall concordant transcriptomic and proteomic changes^38^, with a weaker overlap in upregulated mRNAs and proteins. Notably, the concordance between palmitate-induced transcriptomic and proteomic alterations was enhanced when the tRF was inhibited. This observation may reflect changes in translational regulation. In control cells, palmitate altered the translation efficiency of a subset of transcripts; however, this modulation was no longer observed upon tRF inhibition, suggesting that the tRF may contribute to palmitate-induced translational control.

## Conclusions

Our study uncovers a novel regulatory framework in which tRFs—particularly 5’tRF^Glu(CTC)^—modulate islet responses to diabetogenic stress. In pre-diabetic and early T2D diabetes states, tRFs may act as integrators of environmental signals, coordinating endocrine and immune responses and contributing to islet adaptation or dysfunction.

### Limitations of the study and perspectives

Our results suggest that tRFs respond to metabolic cues and orchestrate islet cell function and intercellular communication. While we focused on 5’tRF^Glu(CTC)^, other fragments such as 5’tRF^Gly(GCC)^ also showed modulation and warrant further study. Future investigations should delineate the specific role of these fragments in islet biology and macrophage activation. In addition, the precise mechanisms by which tRFs influence RBP activity and mRNA processing remain to be elucidated in detail.

Moreover, while our co-culture system captures some essential aspects of iMAC-β-cell interactions, more physiologically relevant 3D models are needed to explore the complex islet microenvironment dynamics. Recent studies using embryonic stem cell-derived organoids have highlighted the importance of iMACs in β-cell maturation^51^. Extending tRF profiling and manipulation to such models could yield critical insights into their roles in islet development and function.

Finally, a major limitation in the field is the lack of in vivo genetic models selectively targeting tRFs. Because tRFs are generated through regulated cleavage of essential tRNAs, conventional knockout approaches would disrupt the parental tRNA pool and global translational homeostasis, rather than specifically interrogating the functional roles of the derived fragments. In vivo delivery of ASOs to target and block tRFs in specific islet cells represents a major and challenging perspective. Targeted delivery to β-cells using glucagon-like peptide-1 receptor (GLP1R) ligand carriers has shown encouraging results^52,53^ and ongoing work is exploring the use of scavenger receptor ligands to reach tissue-resident macrophages^54,55^. Notably, ASO uptake by activated macrophages and subsequent delivery to parenchymal cells has been described^56,57^, highlighting the importance of studying macrophage-cell interactions in the context of tRF-targeted therapies.

## Methods

### Ethical regulation compliance

Our research complies with all relevant ethical regulations. All animal procedures and protocols were approved by the Swiss Research Councils and Veterinary Offices under the animal authorization number VD2495x4. For human samples derived from living organ donors, the observational study protocol (ClinicalTrials.gov NCT02175459) was approved by the Ethical Committee Fondazione Policlinico Universitario Agostino Gemelli IRCCS—Università Cattolica del Sacro Cuore (P/656/CE2010 and 22573/14), and all participants provided written informed consent, followed by a comprehensive medical evaluation, as previously described^58,59^. The use of human tissue from cadaveric donors and all experimental protocols were approved by the French government (Directorate General for Research and Innovation of the Ministry, Bioethics Unit), registered under number DC-2019-3439. Informed consent was obtained from donors’ next of kin.

### Mice

Male db/db mice (genetic background BKS.Cg-*Dock7*^ *m*^ + /+*Lepr* ^*db*^J) and wild-type controls (genetic background BKS.Cg-*Dock7* ^*m*^ + /+) of 8 weeks of age were obtained from Charles River Laboratories (Les Oncins, France). Islet samples from C57BL/KsJ db/db and C57BL/6 J ob/ob mice (13–16 weeks old) and age-matched lean control mice (db/ + C57BL/KsJ and ob/+ C57BL/6 J, respectively) were kindly provided by Professor Ross Laybutt (Australia). Diet-induced obese (SC-DIO-C57J-M) mice were purchased by Janvier Laboratories (Le Genest-Saint-Isle, France), 6-week-old C57BL/6JRj male mice were fed a hypercaloric diet at 5.21 kcal/g containing 20 % protein, 20 % carbohydrate, and 60 % fat named high-fat diet (HFD) and a control diet at 3.82 kcal/g containing 20 % protein, 70 % carbohydrate, and 10 % fat for 16 weeks (SC-CJAGEJ-M). Male and female C57BL/6NRj mice (aged 12–14 weeks) were obtained from Janvier Laboratories (Le Genest-Saint-Isle, France) or derived from our colony. Mice were housed on a 12-h light/dark cycle with a mean ambient temperature of 20-21 °C and 55% humidity with *ad-libitum* chow diet (SAFE^®^−150-SP).

### Living organ donors

Living donors were metabolically profiled before undergoing surgery, as previously described^58,59^, and subjected to a 75 g OGTT and HbA1c testing to diagnose diabetes, according to the American Diabetes Association criteria^60^. Inclusion criteria were: age between 20 and 70 years, candidates for pancreaticoduodenectomy for periampullary neoplasms, HbA1c < 7.0%, fasting triglycerides <200 mg/dL, and LDL cholesterol <160 mg/dL. None of the participants enrolled had a family history of diabetes. Both female and male individuals were included (based on sex assigned at birth).

A standard 75 g OGTT was performed after a 12 h overnight fast with measurements of glucose, insulin and C-peptide at 0, 30, 60, 90, and 120 min after the glucose load. Based on the OGTT, we classified the population as normal glucose tolerant (NGT *n* = 12), impaired glucose tolerant (IGT *n* = 13) and having T2D (T2D *n* = 11), according to the American Diabetes Association criteria^60^. During OGTT, insulin secretion was derived from C-peptide levels by deconvolution. Beta cell glucose sensitivity, i.e. the slope of the relationship between insulin secretion and glucose concentration, was estimated from the OGTT by modeling, as previously described^61,62^. The differences in clinical parameters among the three study groups (NGT, IGT, and T2D) were evaluated using a two-sided one-way analysis of variance (ANOVA), followed by Bonferroni-adjusted pairwise comparisons. A summary of the results can be found in Table S1 of Supplementary Information.

### Cell lines

MIN6B1 cells, a subclone of the murine insulin-secreting cell line derived from insulinoma^63^, were kindly provided by Dr. Philippe Halban and cultured at a density of 1.5 × 105 cells/cm^2^ in DMEM-GlutaMAX medium (Cat. 10566016, ThermoFisher Scientific) containing 25 mM glucose and 4 mM L-glutamine, and supplemented with 15% fetal calf serum, 70 mM b-mercaptoethanol, 50 mg/mL streptomycin and 50 IU/mL penicillin. All cell lines were cultured at 37 °C in a humidified atmosphere (5% CO_2_, 95% air) and tested negative for mycoplasma contamination.

### Mouse islet isolation and cell sorting

Mouse pancreases were digested with collagenase (Sigma), and islets were isolated by Histopaque (Sigma) density gradient and handpicking^64^. For the indicated experiments, mouse islets were dissociated into single cells by incubation in Ca^2+^/Mg^2+^ free phosphate-buffered saline, 3 mM EGTA, and 0.002% trypsin (ThermoFisher) for 2–3 min at 37 °C. Isolated mouse islets and dissociated cells were cultured in RPMI 1640 GlutaMAX medium (ThermoFisher) supplemented with 10% FCS, 100 U/mL penicillin and 100 µg/mL streptomycin, 1 mM sodium pyruvate (Sigma), and 10 mM Hepes (Sigma).

For fluorescence-Activated Cell Sorting (FACS), islets from two db/db mice and three wild-type mice were pooled; 600-800 islets per preparation were dissociated. Fractions enriched in β-cells were obtained based on β-cell autofluorescence, as previously described^65^. Islet-resident macrophages (iMACs) were sorted based on the expression of CD45, CD11b, F4/80 and CD11c markers^6^. Dissociated islet cells were washed once with FACS buffer (0.1% BSA, 2 mM EDTA, 11 mM glucose in PBS) and incubated for 5 min with TruStain FcX™ (anti-mouse CD16/32) Antibody (Cat. 101319, BioLegend), at 4 °C. Then cells were incubated for 30 min in the dark at 4 °C with the following antibodies: 1:200 of FITC anti-CD45 (Cat. 103108, BioLegend), 1:100 brilliant violet CD11b (Cat. 101236, BioLegend), 1:100 APC F4/80 (Cat. 123116, BioLegend) and 1:100 PE CD11c (Cat. 117308, BioLegend). Cells were then washed twice with FACS buffer and sorted by FCF-Aria-II (SORP). β-cell purity was assessed as previously described^66^. On average, β-cell fractions contained 99.1  ±  0.9% insulin-positive cells and 0.6  ±  0.6% glucagon-positive cells.

### Bone marrow-derived macrophage differentiation

Bone marrow was isolated from tibia and femurs of 10-12 weeks old C57Bl/6 N mice. Red blood cells were lysed in RBC lysis buffer containing 0.155 M NH_4_Cl, 10 mM KHCO_3_, 0.127 M EDTA. Bone marrow cells were differentiated for 7 days on not-tissue culture-treated 100 mm petri dishes in basal BMDM medium composed by DMEM (Cat. 61965-059, ThermoFisher Scientific), 10 mM HEPES, 10% FCS, 50 μg/mL streptomycin and 50 IU/mL penicillin, supplemented with 10 ng/ml macrophage colony-stimulating factor (M-CSF, 315-02 Peprotech). After 7 days, bone marrow-derived macrophages (BMDMs) were further polarized into anti-inflammatory M2 Mφs by exposure to 10 ng/ml IL-4 and IL-13 for 48 h, and pro-inflammatory M1 Mφs by exposure to 100 ng/ml lipopolysaccharide (LPS, Cat. L6529, SigmaAldrich) and 50 ng/ml IFN-γ for 48 h. Differentiation and polarization were monitored by the expression of specific gene markers measured by qPCR.

### Pancreas samples and laser capture microdissection of islets from living donors

Pancreas samples were collected from male and female (sex assigned at birth) living donors undergoing pylorus-preserving pancreatoduodenectomy as part of their clinical care for neoplasms, recruited at the Digestive Surgery Unit and studied at the Centre for Endocrine and Metabolic Diseases Unit (Agostino Gemelli University Hospital, Rome, Italy).

Indications for surgery were periampullary tumors, pancreatic intraductal papillary tumors, mucinous cystic neoplasm of the pancreas, and nonfunctional pancreatic neuroendocrine tumors. Surgical pancreatic tissue specimens were snap frozen in liquid nitrogen and stored at −80 °C, embedded in Tissue-Tek OCT compound.

Pancreatic human tissue samples from living donors were cryosectioned. Sections were fixed in 70% ethanol for 30 s, dehydrated in 100% ethanol for 1 min, in 100% ethanol for 1 min, in xylene for 5 min and finally air-dried for 5 min. Laser capture microdissection (LCM) was performed using an Arcturus XT Laser-Capture Microdissection system (Arcturus Engineering, Mountain View, CA, USA) by melting thermoplastic films mounted on transparent LCM caps (Cat. LCM0214 - ThermoFisher Scientific, Waltham, MA, USA) on specific islet areas. Human pancreatic islets were subsequently visualized through islet intrinsic autofluorescence for LCM procedure. Thermoplastic films containing microdissected cells were incubated with 10 µl of extraction buffer (cat. kit0204 - ThermoFisher Scientific, Waltham, MA, USA) for 30 min at 42 ◦C and kept at −80 °C until RNA extraction. Each microdissection was performed within 30 min from the staining procedure in a contamination-free, dehumidified environment, maintaining the external temperature at 16 °C to preserve RNA integrity. Overall, an endocrine area of microdissected islets of about 2×106 um2 for each subject was analyzed for the global molecular analysis.

### Human islet isolation from cadaveric donors

Human islets from brain-dead donors were obtained from the Centre d’Etude Européen pour le Diabéte in Strasbourg. Upon arrival, human islets were dissociated, and cells were cultured in CMRL medium (Cat. 11530037, ThermoFisher Scientific) supplemented with 10% fetal calf serum, 10 mM HEPES pH 7.4, 100 mg/mL streptomycin, 100 IU/mL penicillin and 2 mmol/l glutamine. To perform experimental replicates, a single human islet preparation was dissociated on different days, and downstream manipulations were performed independently. Independent preparations are defined as human islets derived from distinct donors. Detailed information about human islet donors is provided in Table S5.

### β-cell and macrophage co-culture

After differentiation in p100 dishes, BMDMs were detached with Accutase (Cat. A1110501, ThermoFisher Scientific) and plated in direct co-culture with MIN6 cells at a ratio of 1:10. Cells were kept for 72 h in co-culture and then used for downstream analysis. Alternatively, after 24 h, co-cultured cells were treated either with 0.5 mM palmitate or with 0.75% BSA as a control for an additional 48 h. Insulin secretion assay was performed in co-cultures, and for gene expression analysis, the two cell populations were purified by FACS by staining macrophages with antibodies against F4/80 and CD45.

### Cell transfection and treatment

For tRF inhibition, MIN6 cells, primary mouse and human islets, and BMDMs were transfected with 50 pmol custom miRCURY LNA miRNA inhibitor (Cat. 339141, Qiagen) or miRCURY LNA miRNA inhibitor control (Cat. 339126, Qiagen) and 2 μl of lipofectamine 2000 (Cat. 11668019, ThermoFisher Scientific) in a 24-well plate. The specific sequence targeted by custom inhibitors is indicated in Supplementary data_2. For Angiogenin silencing, MIN6 cells were transfected with 20 pmol of stealth siRNA targeting Angiogenin (Cat. 1320003, siRNA ID: MSS235930, MSS235928, MSS235929 ThermoFischer Scientific) or siRNA negative control (12935300, ThermoFisher Scientific) and 2 μl of lipofectamine 2000 in a 24-well plate. After overnight incubation, medium was replaced with complete medium or with treatment medium. Oleic, stearic and palmitic acids were added at 0.5 mM concentration complexed with fatty-acid-free BSA (Sigma - Aldrich). Control treatment was performed in 0.75% fatty-acid free BSA. Fatty-acid treatments were performed in 5% FBS medium for MIN6 and BMDMs, and 1% FBS medium for primary cells. Transfection experiments were performed in Primary cells were obtained from both male and female mice and individuals (sex assigned at birth).

### RNA purification

Total RNA was extracted from each LCM sample using PicoPure RNA isolation kit Arcturus (cat. kit0204 - ThermoFisher Scientific, Waltham, MA, USA) following manufacturer’s procedure. The characterization of RNA samples was performed with Agilent 2100 Bioanalyzer technology with RNA Pico chips (cat. 5067–1513 Agilent Technologies, Santa Clara, CA, USA): we considered as good quality samples those that showed a RIN higher than or equal to 4.5 (scale: 1-10). Total RNA was extracted from mouse and human primary islet cells and macrophages, FACS-sorted cells and MIN6 cells using the miRNeasy micro kit (Qiagen). For sequencing applications, RNA with RIN higher than 7 was used.

### Real-time qPCR

Real-time PCR quantification of miRNAs and tRFs. was performed using the miRCURY LNA Universal RT microRNA PCR system (Qiagen). The sequences used for custom primer design are indicated in Supplementary Data 2. For gene expression quantification, RNA was treated with DNase and reverse transcribed with a Moloney Murine Leukemia Virus reverse transcriptase and random primers. Quantitative PCR (qPCR) was performed using SsoAdvanced Universal SYBR Green Supermix. Primer sequences are listed in Supplementary Data 2. The 2^-ΔΔCt^ method was used to analyzed qPCR data. In unpaired experiments, the average of the control ΔCt was used as a reference. Expression of miRNAs, tRFs and protein-coding genes was corrected for the level of miRNAs/ncRNAs and housekeeping genes that are unaffected by the experimental condition under study, as indicated in figure axis labeling.

### Assessment of full-length tRNA levels and aminoacylation

The tRNA levels and tRNA aminoacylation rates were assessed by a qPCR-based methodology based on a previously published protocols^67^. RNA was purified from MIN6 cells cultured in 6-well plates by phenol-chloroform extraction and precipitated with isopropanol. Samples were then resuspended in 10μl of cold tRNA resuspension buffer (10 mM sodium acetate buffer, pH 4.5, 1 mM EDTA) and concentration was measured at nanodrop. From the same sample, 2 μg of RNA were incubated with 100 mM Sodium Acetate buffer in the presence of 10 mM NaIO_4_ (for oxidized reaction) or NaCl (for control). In the oxidized reaction, amino-acylated tRNAs are protected, while uncharged tRNAs lose their 3’A residue. After 20 min at room temperature, reactions were quenched with 125 mM glucose. After adding a spike-in oligonucleotide, RNA was precipitated with 1 μl glycogen, 50 mM NaCl and 100% EtOH. In order to induce tRNA de-aminoacylation, RNA pellets were resuspended in 100 μl of 50 mM Tris, pH 9 and incubated at 37 °C for 45 min and then quenched with 50 mM sodium acetate buffer (pH 4.5) and 100 mM NaCl. After overnight precipitation with 100% EtOH, the samples were cleaned up with Zymo kit, dissolved in 5μl RNAse-free water. 800 ng of RNA was used for adaptor ligation with T4 RNA Ligase2 KQ (NEB) and then for retro transcription with SuperScript^TM^ RT IV (Thermo Fisher). cDNA was amplified with specific primers to detect tRNA^Glu(CTC)^, and aminoacylation was assessed by the difference in Ct values between oxidized and control reaction samples. Oligonucleotides used for assessment of full-length tRNAs are reported in Supplementary Data 2.

### tRF profiling

tRF profiling was performed by small RNA sequencing. cDNA libraries were prepared with QIAseq miRNA kit (Qiagen) from RNA of FAC-sorted β-cells and iMACs of male db/db and wild-type mice, and from LCM-islets of female and male (sex assigned at birth) living donors. Single-end sequencing was performed on NovaSeq instrument (Illumina).

Following adapter sequences removal, identical reads longer than 16 nts were collapsed based on Unique Molecular Identifiers and aligned to the mouse genome (GRCm38.p6) or the human genome (hg38). Aligned reads were mapped to the mature tRNA sequences from the GtRNAdb database (http://gtrnadb.ucsc.edu/) and to the 22 mouse mitochondrial tRNA sequences from https://www.ncbi.nlm.nih.gov using bowtie (version 1; http://bowtie.cbcb.umd.edu). Bowtie parameters were set to output only perfect matches to tRNA sequences. Differentially expressed tRFs were identified using edgeR (version 4.2.2) in R packages (http://bioconductor.org). For each tRF, *p* values and false discovery rates (FDRs) were obtained based on the model of negative binomial distribution. Fold changes of tRF expression were also estimated within the edgeR package. Individual processed data disaggregated for sex are reported as dataset in Supplementary Data 1.

### Correlation analysis

Associations between the levels of tRFs detected by small RNA sequencing and clinical parameters of living donors were evaluated using linear models. tRFs normalized counts were converted in log_2_ scale after the addition of a pseudo count. To exclude influent values from the regression analysis, points with a Cook’s distance >5 folds from the average Cook’s distance of the points in the regression model were removed. Linear models were then fitted for each tRF with each clinical parameter, implementing age, gender, and BMI as covariates. Regressions with a *p*-value associated with the coefficient of the clinical parameter <0.05 were considered statistically significant. For representative purposes, the log_2_-transformed counts were corrected for the effect of the covariates for each regression. The correction was performed by estimating the effect of the covariates in each regression as the coefficient assigned to the covariate multiplied by the value of the covariate. Then, the effect of the covariates for each data point in the regression was summed up and then subtracted from the normalized log_2_-scaled counts.

### Pull-down and proteomics analysis

MIN6 cells were plated in 100 mm dishes and transfected with 20 nM biotinylated 5’tRF^Glu(CTC)^ mimic (GLU) or scramble control (CTRL) using 50 µl Lipofectamine 2000 (Thermo Fisher). After 48 h, cells were washed in cold PBS, crosslinked at 254 nm (200 mJ/cm²), and lysed in harsh buffer (150 mM NaCl, 50 mM Tris-HCl pH 7.5, 1% NP40, 0.5% sodium deoxycholate, 0.1% SDS). Lysates were clarified by centrifugation (10 min, 13,000 g), and incubated with M-280 streptavidin beads (Thermo Fisher). Inputs were stored. Proteins were processed using the SP3 method: eluted with 2% SDS/10 mM DTT/50 mM Tris pH 7.5, alkylated with 32 mM iodoacetamide, precipitated on Sera-Mag beads (ethanol 60%), and digested with trypsin. Peptides were desalted on SCX plates and analyzed by nanoLC-MS/MS (Vanquish Neo–Orbitrap Exploris 480, 130 min C18 gradient, DDA-HCD). The experiment was performed on 4 independent passages on MIN6 cells; a total of 8 samples were processed.

For BMDM-M2 macrophages, bone marrow-derived M0 cells were polarized with IL-4/IL-13 for 48 h. For iMACs, M0 macrophages were differentiated in co-culture with MIN6-βcells for 24 h and then exposed to either BSA or PA for 48 h. M2 and iMAC-like macrophages were lysed in harsh buffer, and incubated with GLU or CTRL mimics for 1 h at RT before pulldown on streptavidin beads. Protein digestion followed the miST protocol: deoxycholate buffer (1% SDC, 100 mM Tris pH 8.6, 10 mM DTT), heat denaturation, Trypsin/LysC digestion, chloroacetamide alkylation, overnight digestion, SCX desalting. Peptides were separated on a 45 cm C18 column (140 min gradient) and analyzed on a Fusion Orbitrap (DDA-HCD). The experiment was performed on 5 BMDM-M2 and 2 iMAC-like macrophages, with independent differentiations; a total of 14 samples were processed.

Raw data were processed with MaxQuant 2.4.7.0 against the *Mus musculus* UniProt database (March 2023), with 1% FDR. iBAQ values were log2-transformed, filtered, and analyzed via Student’s *t* test with Benjamini–Hochberg correction (FDR < 0.05). Complete protocols are provided in Supplementary Methods.

### Proteomics

For proteomic characterization, mouse islets from 3 independent mouse islet isolations were dispersed and transfected with either Ctr or αGlu ASOs before PA treatment. A total of 12 samples were processed. Cell lysates in RIPA buffer were processed using the SP3 protocol^68^. After dilution in SDS/DTT buffer and heat denaturation, proteins were alkylated and precipitated onto Sera-Mag Speedbeads with ethanol. Digestion was performed with trypsin, followed by desalting on SCX plates. Peptides were analyzed on a TIMS-TOF Pro mass spectrometer coupled to an EvoSep One LC system using a DIA-PASEF method with a 68 min gradient. Ion mobility and mass range selection followed established parameters^69^. DIA data were analyzed with Spectronaut 19.9 using the Pulsar engine and a *Mus musculus* UniProt reference proteome (February 2025). Peptides were quantified by XIC area, and protein groups inferred using the MaxLFQ algorithm. After log2 transformation and filtering, missing values were imputed, and differential expression was evaluated using Student’s *t* test with Benjamini–Hochberg correction (FDR < 0.05). Detailed methods are available in Supplementary Information.

### Pull-down of mRNA in active translation

The AHARIBO^TM^ kit (IMMAGINA BioTECHNOLOGY) was used to isolate mRNA associated with active ribosomes. Briefly, MIN6 cells were seeded in a 6-well plate, transfected with specific ASOs and treated with palmitate for 48 h. On the day of collection, the cells were incubated for 40 min with methionine-free complete DMEM and then supplemented with l-azidohomoalanine (AHA) methionine analog for 10 min followed by 5 min sBlock treatment to immobilize nascent peptides on the active ribosomes. Upon cell lysis, AHA-labeled nascent peptides were used as tags to bind magnetic beads with a click chemistry reaction and to purify ribosomes complexed to mRNA in translation. RNA extracted from total cell lysates was used as input. RNA sequencing was then performed with mRNA pulled-down (PD, in translation) and with inputs.

### mRNA sequencing and translation efficiency analysis

For transcriptomic analysis, RNA was obtained from mouse islets or in vitro differentiated anti-inflammatory M2 Mφs. For translation efficiency analysis, RNA derived from MIN6 total cell lysates (input) or AHARIBO pull-down (PD) was used. Library preparation was performed with the Illumina Stranded mRNA Library Prep kit and sequenced with an AVITI or a NovaSeq6000 instrument using the SBS chemistry v4 (Illumina). After demultiplexing, read annotation and normalization were performed using the QIAGEN RNA-seq Analysis Portal v 5.1.

For mouse islet and anti-inflammatory Mφs transcriptomic analysis, differential expression was carried out with the QIAGEN RNA-seq Analysis Portal v 5.1 that computes differential expression using a negative binomial generalized linear model with two-sided tests and Benjamini–Hochberg FDR correction.

Translatome analysis was performed using the *limma* R package v.3.66.0^70^. Translation efficiency (TE) was calculated by performing the ratio between PD and input mRNA transcript normalized counts.

### Bioinformatic tools and software

FACS data were analysed using FlowJo v.11 software (BD Biosciences). Gene and protein lists were analysed for pathway enrichment using ShinyGO v.0.82 tool^71^. KEGG and GO enrichments with FDR *p* value < 0.1 were considered significant. Rank-rank hypergeometric overlap was performed using the R-package RedRibbon v.1.3.1^37^. Transcriptomic data of M2 macrophages were run on MacSpectrum web tool v.1.0.1^40^. Statistical analysis was performed with Graphpad Prism 11.0.0 (GraphPad Software, Boston, Massachusetts, USA, www.graphpad.com).

### Western blot and immunofluorescence

Proteins from cellular lysates were migrated on 10% SDS-PAGE gels. Following electrophoresis, proteins were transferred onto PVDF membranes and blocked at room temperature in Tris-buffered saline/0.1% Tween20 containing 5% of BSA. The membranes were then incubated overnight at 4 °C in 5% BSA with primary antibodies specific for Msi2 (Cat. 10770-1-AP, Proteintech) and HnRNP-A3 (Cat. 25142-1-AP, Proteintech). The bands were detected by chemiluminescence (Pierce) after incubation with a horseradish peroxidase-conjugated secondary antibody (Goat Anti-Rabbit IgG (H + L)-HRP Conjugate 1706515 and Goat Anti-Mouse IgG (H + L)-HRP Conjugate 1706516, Bio-Rad).

For immunofluorescence, cells were plated on chamber slide wells (Ibidi) and fixed with 4% paraformaldehyde. Cells were permeabilized with 90% methanol, blocked in 1X TBS buffer with 5% bovine serum albumin (BSA) and 0.1% Triton. Slides were then incubated with primary antibodies against mouse insulin (Cat. 66198-1-Ig I, Proteintech) and cleaved caspase 3 (Cat. 9661, Cell signaling) and signal was detected with fluorescent secondary antibodies (Goat anti-Rabbit Alexa Fluor™ 568 Cat # A-11011, Goat anti-Mouse Alexa Fluor™ 488 Cat # A-11001, ThermoFisher Scientific).

Complete list of antibodies is provided in Supplementary Information Table S6.

### Insulin secretion

MIN6B1 alone or in co-culture with iMAC-like macrophages and dispersed human islets were pre-incubated for 50 min at 37 °C in Krebs-Ringer bicarbonate buffer (KRBH) containing 25 mM HEPES, pH 7.4, 0.1 % BSA (Sigma-Aldrich) and 2 mM glucose. The cells were then incubated for 50 min in KRBH containing 0.5% BSA and 2 mM glucose, and the media were collected (basal insulin secretion). The cells were incubated for 50 min in KRBH containing 0.5% BSA and 20 mM glucose, and the media were collected (stimulated insulin secretion). Total cellular insulin contents were recovered in acid-ethanol (0.2 mM HCl in 75% ethanol). Insulin levels were measured using an insulin enzyme immunoassay kit (Mercodia). For normalization with protein content, cells were lysed in RIPA buffer, and proteins were quantified by Bradford assay (Thermo Fischer). All experiments were performed in quadruplicate.

### Reporting summary

Further information on research design is available in the Nature Portfolio Reporting Summary linked to this article.

## Supplementary information

Supplementary information

Description of Additional Supplementary Files

Supplementary data 1

Supplementary data 2

Reporting Summary

Transparent Peer Review file

## Source data

Source data

## Data Availability

The small RNA sequencing data data generated in this study have been deposited in the GEO database under accession code GSE302055 and GSE302056. The processed data of small RNA sequencing from LCM human islets generated in this study are provided in the Supplementary Data 1 file. The mass spectrometry data derived from pull-down have been deposited in the ProteomeXchange database under accession code PXD065726, PXD065736 and PXD073702. The mRNA sequencing data generated in this study are provided in the GEO database under accession code: GSE302250 and GSE302527. Proteomic data generated in this study are provided in the ProteomeXchange database under accession code PXD065746. The processed data generated in this study from translatomic experiments are in Zenodo repository under 10.5281/zenodo.18978118^72^. Human islet bulk RNA sequencing and proteomic data were downloaded from humanislets.com database; the raw RNA sequencing data are deposited in the European Genome-phenome Archive (EGA) under accession code EGAS00001007241, while raw proteomics data are deposited to ProteomeXchange via MASSive under accession code PXD045422. All other data supporting the findings described in this manuscript are available in the article and in the Supplementary Information. Source data are provided with this paper.
