# Supplementary material for: 5’tRNA-derived fragments modulate β-cell homeostasis and islet macrophage activation in type 2 diabetes
Source: Nat Commun. 2026 May 4;17:5989. doi: 10.1038/s41467-026-72641-z (PMC13346763; doi:10.1038/s41467-026-72641-z)
Supplement: Supplementary file 1 — Supplementary information [file 41467_2026_72641_MOESM1_ESM.pdf]

Supplementary information for manuscript #NCOMMS-25-68607A

“5’tRNA-derived fragments modulate β-cell homeostasis and islet macrophage activation in type 2 diabetes”

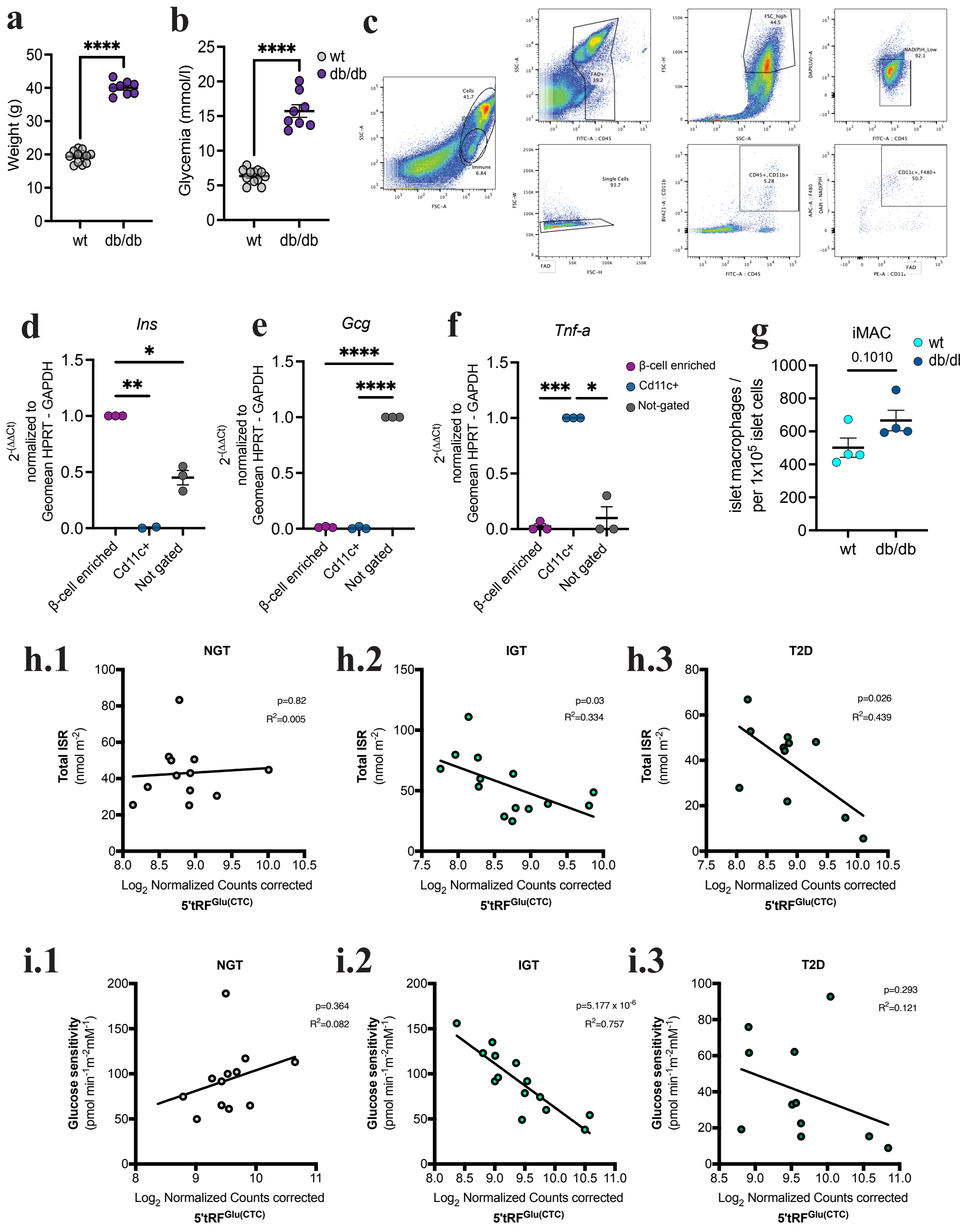

Figure S1: Blood glucose concentration (**a**) and body weight (**b**) were measured in 8 weeks old db/db mice (purple dots) and lean wild type (wt, grey dots) controls (n=12 wt and 8 db/db mice). ****p<0.0001 db/db vs wt by two-sided unpaired Student t-test, exact p values: p= (a) p= (b). Islets from 4 wt and 2 db/db were pooled for FACS. Gating strategy used for parallel FACS sorting of islet macrophages (Cd11c$^+$, iMACs) and β-cells (**c**). qPCR measures of cell markers in β-cell-enriched (purple dots), Cd11c+ (blue dots) and not-gated (grey dots) fractions (**d-f**, n=3 independent preparations). *p<0.05, **p<0.01, ***p<0.001, ****p<0.0001. Exact p values: d)

p=0.0064 cd11c^{+} vs β-cells, p=0.266 not-gated vs β-cells by mixed effect model with Sidak correction for multiple comparisons; e) p=0.000006 β-cells vs not-gated, p=0.00007 cd11c^{+} vs not-gated by One-way ANOVA with Sidak correction for multiple comparisons.; f) p=0.0009 β-cells vs cd11c+, p=0.0188 not-gated vs cd11c^{+} by One-way ANOVA with Sidak correction for multiple comparison. The numbers of iMACs from wt (light blue) and db/db (dark blue) islets were plotted in (**g**, n=4 independent preparations), statistic test was two-sided unpaired Student t-test. Data are presented as mean values +/- SEM. Source data are provided as a Source Data file. The levels of 5'tRF$^{Glu(CTC)}$ detected in small RNA sequencing from patient subgroups were correlated with total insulin secretion rate (**h.1-3**) and glucose sensitivity (**i.1-3**). The association of Log_2 scaled tRF normalized counts with clinical/metabolic parameters was corrected for the covariates (Age, Sex, BMI). Statistical significance of the association was assessed using two-sided t-tests on the regression coefficients, and regressions with p-values < 0.05 were considered statistically significant.

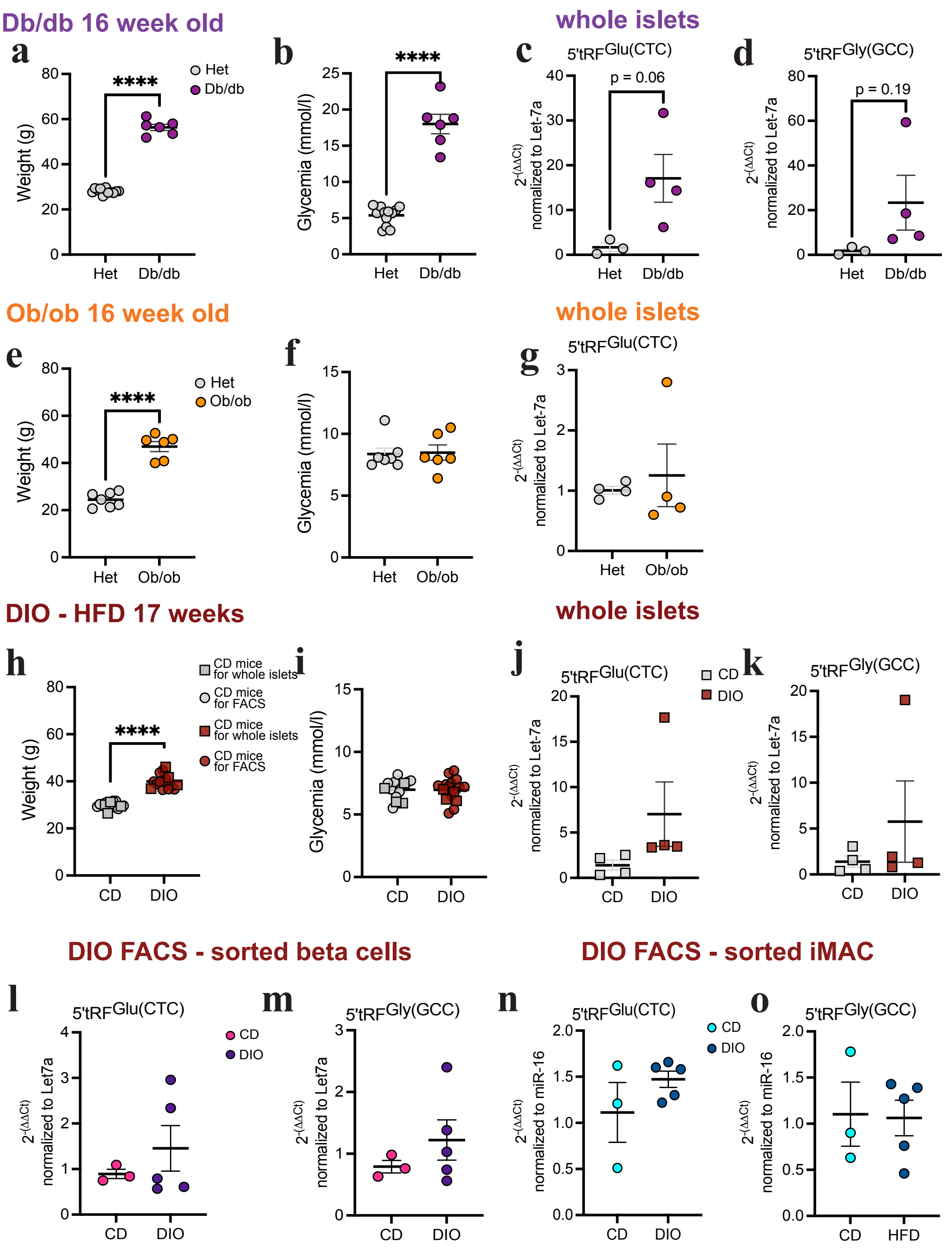

Figure S2: **a-d**) 16-week-old C57BL/KsJ db/db mice (purple dots) and age-matched heterozygous controls (grey dots). Blood glucose levels (**a**) and weight (**b**) were measured (n=6 db/db and n=12 het mice). qPCR to assess 5'tRF levels was performed with RNA from islets, samples from 4 heterozygous controls and 1-2 db/db were pooled (n=3 Het and n=4 db/db independent preparations, **c-d**). p values were computed by two-sided student t-test ****p<0.0001 db/db vs Het, a) p=0.000000000000009, b) p=0.000000002 **e-g**) 16-week-old ob/ob mice (orange dots) and

age-matched heterozygous controls (grey dots). Blood glucose levels (**e**) and weight (**f**) were measured (n=6 ob/ob and n=7 het mice independent preparations). qPCR to assess 5'tRF levels was performed with RNA from islets, samples from 1-2 heterozygous controls or ob/ob were pooled (n=4 independent preparations, **g**). p values were computed by two-sided student t-test ****p<0.0001 ob/ob vs Het, e) p=0.000001 **h-o**) C57BL/6J mice fed high-fat diet (HFD) for 16 weeks and age-matched normal chow diet (CD) fed controls. Weight (**h**) and blood glucose (**i**) were measured (n=16 HFD, red dots and CD, grey dots). Mice used for whole islet isolation are displayed in square dots (n=4 independent preparations), islets from 4 CD mice and 2-3 HFD mice were pooled for FACS sorting (round dots). p values were computed by two-sided student t-test ****p<0.0001 HFD vs CD, h) p=0.0000000000002. qPCR to assess 5'tRF levels was performed with RNA from whole islets (**j-k**, n=4 independent preparations) and FACS-sorted beta cells (**l-m**) and iMACs (**n-o)** n=3 CD and 5 DIO independent preparations). Data are presented as mean values +/- SEM. Source data are provided as a Source Data file.

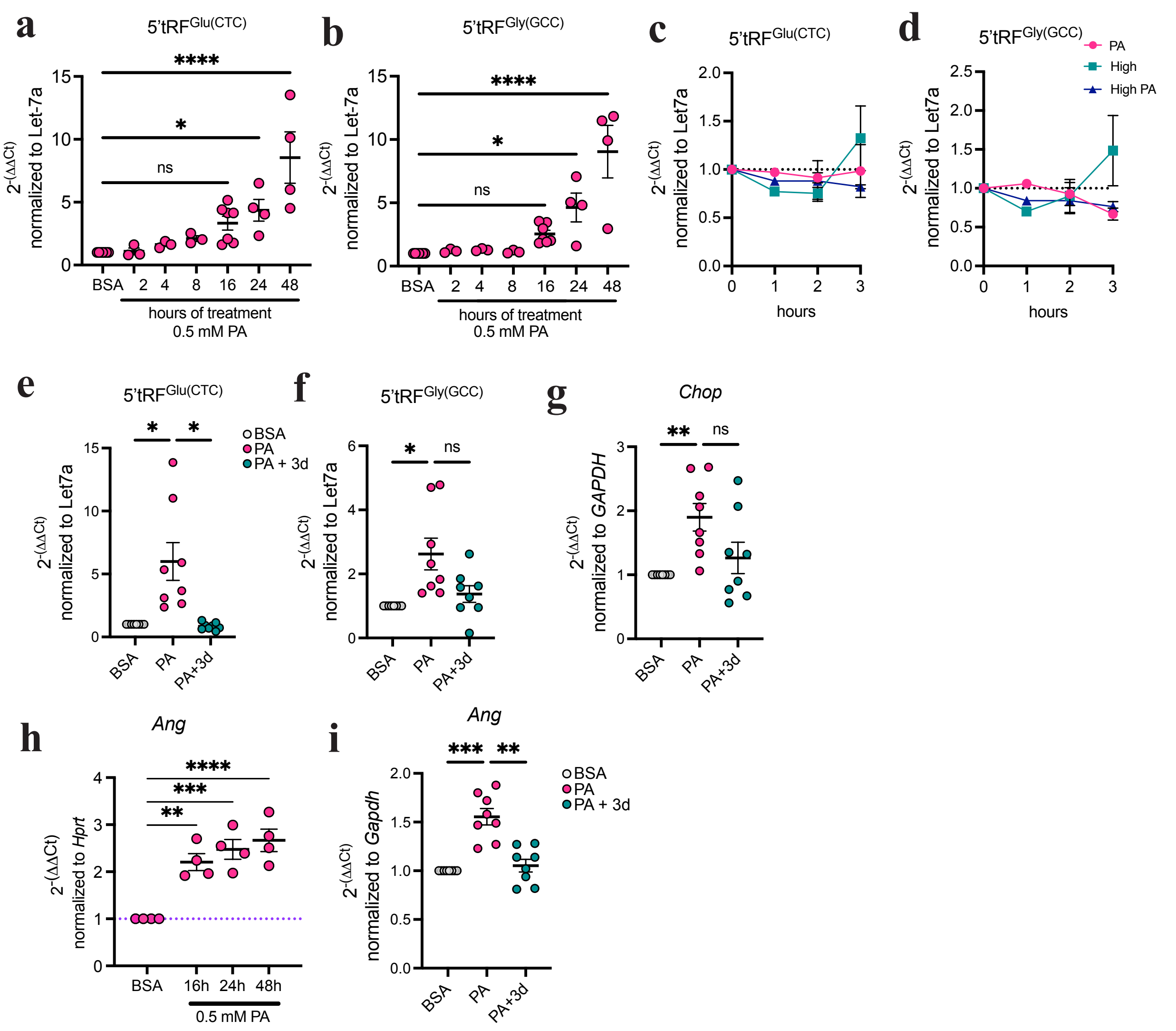

Figure S3: The expression of 5'tRFs was assessed by qPCR in MIN6 β-cells in different time points of PA exposure (**a-b**, independent experiments n=3 2/4/8-hour time points, n=6 BSA and 16-hour time point, n=4 24/48-hour time points) and in mouse islets during an acute exposure time course (1-3 hours) of high glucose (20mM, blue dots) or PA in combination to low (2 mM, pink dots) or high (20 mM, purple dots) glucose concentrations (**c-d,** independent experiments n=1 1-hour time point n=3 2/3-hour time points). *p<0.05, ****p<0.0001 by One-way ANOVA with Sidak correction for multiple comparisons: a) p= 24h PA vs BSA, p= 48h PA vs BSA, b) p= 24h PA vs BSA, p= 48h PA vs BSA. The expression of 5'tRFs and *Chop* was assessed by qPCR in mouse islets in control BSA condition (grey dots), upon 48h PA exposure (pink dots) and after 3 days wash-out period (blue dots) (**e-g**, n=8 independent experiments). *p<0.05; **p<0,01 by One-

way ANOVA with Sidak correction for multiple comparisons: e) p=0.0249 PA vs BSA, p=0.0228 PA+3d vs PA f) p=0.0269 PA vs BSA, g) p=0.0080 PA vs BSA. Angiogenin (*Ang*) expression was evaluated in a time-course PA treatment in MIN6 (**h**, n=4 independent experiments) and in mouse islets subjected to 48h PA treatment plus wash-out (**i**, n=8 independent experiments). **p<0,01; ***p<0.001; ****p<0.0001 by One-way ANOVA with Sidak correction for multiple comparisons: h) p=0.0015 16h PA vs BSA, p=0.00027 24h PA vs BSA, p=0.00009 48h PA vs BSA, i) p=0.0006 PA vs BSA, p= 0.0071 PA+3d vs PA. Data are presented as mean values +/- SEM. Source data are provided as a Source Data file.

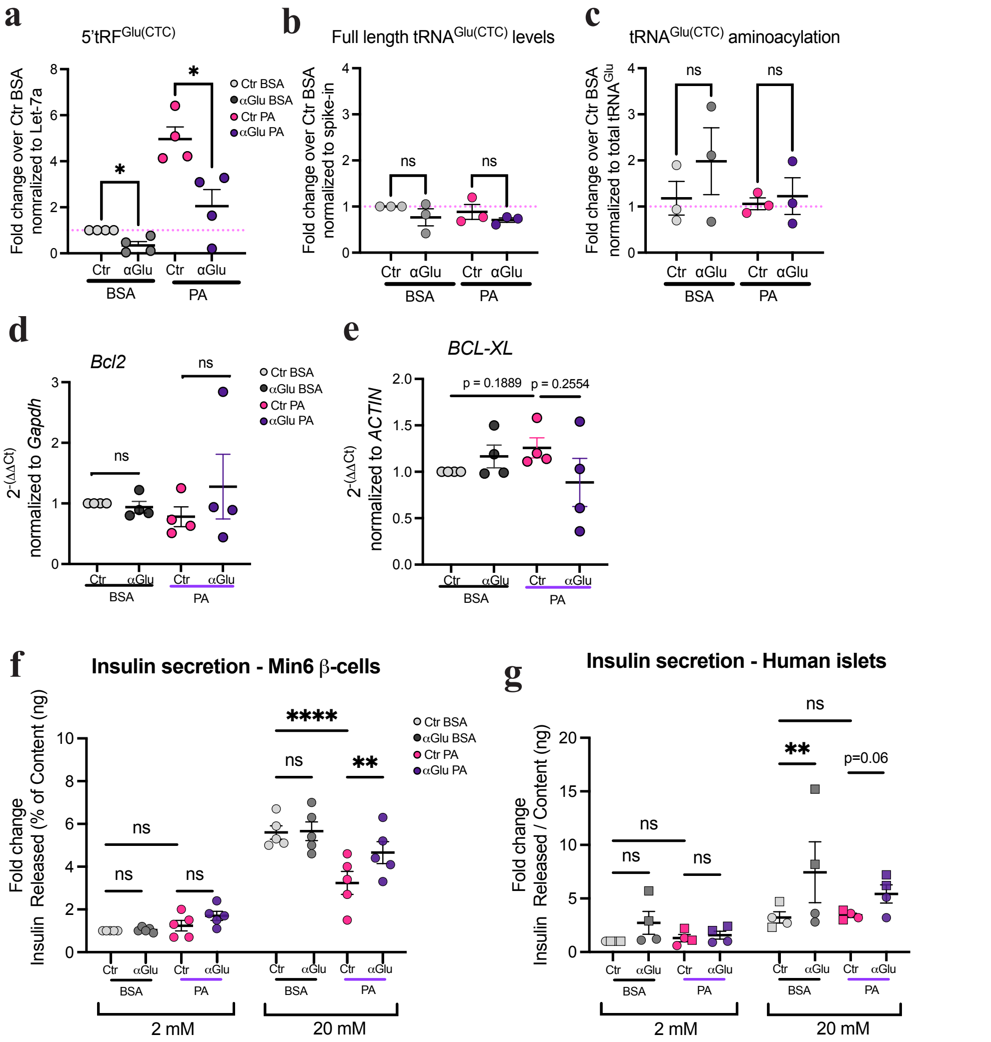

Figure S4: Functional studies were carried our transfecting MIN6 cells (a-c and f), mouse islet cells (d) and human islet cells (e and g): grey dots represent cells transfected with Ctr ASO treated with BSA, dark grey dots are cells transfected with **α**Glu ASO treated with BSA, pink and purple

dots are cells treated with PA upon transfection with Ctr or **α**Glu ASO, respectively. Following transfection in MIN6 cells, the levels of the 5'tRF$^{Glu(CTC)}$ (**a**, n=4 independent experiments) and of the full length tRNA$^{Glu(CTC)}$ (**b,** n=3 independent experiments) were measured. The aminoacylation rates of tRNA$^{Glu(CTC)}$ were also assessed in the different conditions (**c,** n=3 independent experiments). Bcl2 expression was assessed by qPCR in mouse islets transfected with **α**Glu or Ctr ASO and treated with PA for 48h (**d**, n=4 independent experiments). *BCL-XL* expression was assessed by qPCR in human islets transfected with **α**Glu or Ctr ASO and treated with PA for 48h (**e**, n=4 independent experiments). One-way ANOVA with Sidak correction for multiple comparisons was applied to a-e; *p<0.05, exact p values: a) p=0.0296 **α**Glu BSA vs Ctr BSA, p=0.0139 **α**Glu PA vs Ctr PA. Insulin release assessed during an insulin secretion assay was expressed as percentage of the total content in MIN6 (**f**, n=5 independent experiments) and human islets (**g** n=2 technical replicates from n=2 independent preparations, symbol shape indicates the specific preparation) upon ASO transfection and PA treatment. Two-way ANOVA with Sidak correction for multiple comparisons was applied to f-g; **p<0,01; ****p<0.0001, exact p values: f) p=0.00005 Ctr PA vs Ctr BSA in 20mM glucose, p=0.0049 **α**Glu PA vs Ctr PA in 20mM glucose; g) p=0.0015 **α**Glu BSA vs Ctr BSA in 20mM glucose. Data are presented as mean values +/- SEM. Source data are provided as a Source Data file.

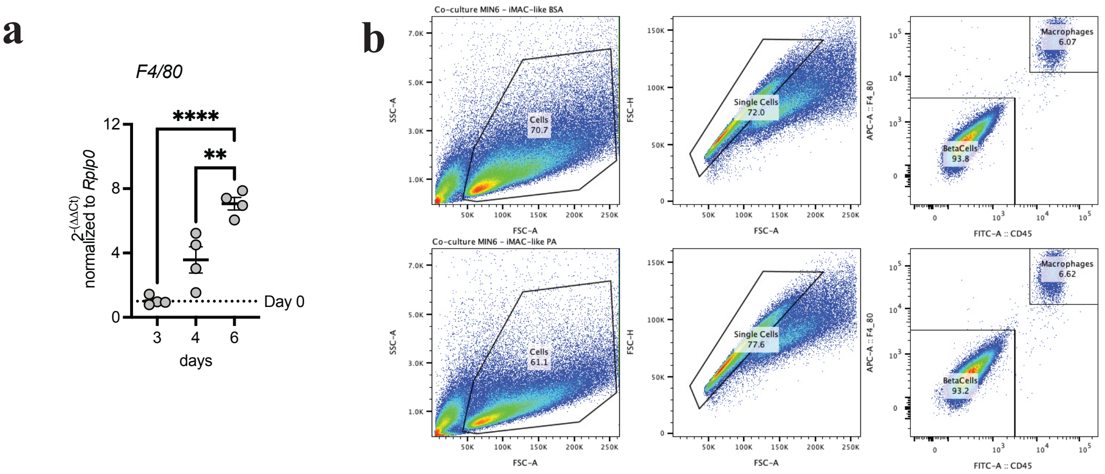

Figure S5: Bone marrow derived macrophages differentiation was assessed by the expression of *F4/80* (**a**, n=4 independent experiments). Data are shown as mean values +/- SEM of fold changes over day 0. **p=0.0030 6d vs 4d, ****p=0.00005 6d vs 3d by One-way ANOVA with Sidak

correction for multiple comparisons. Source data are provided as a Source Data file. Representative image of FACS gating strategy was used to separate macrophages and beta cells at the end of co-culture experiments (**b**).

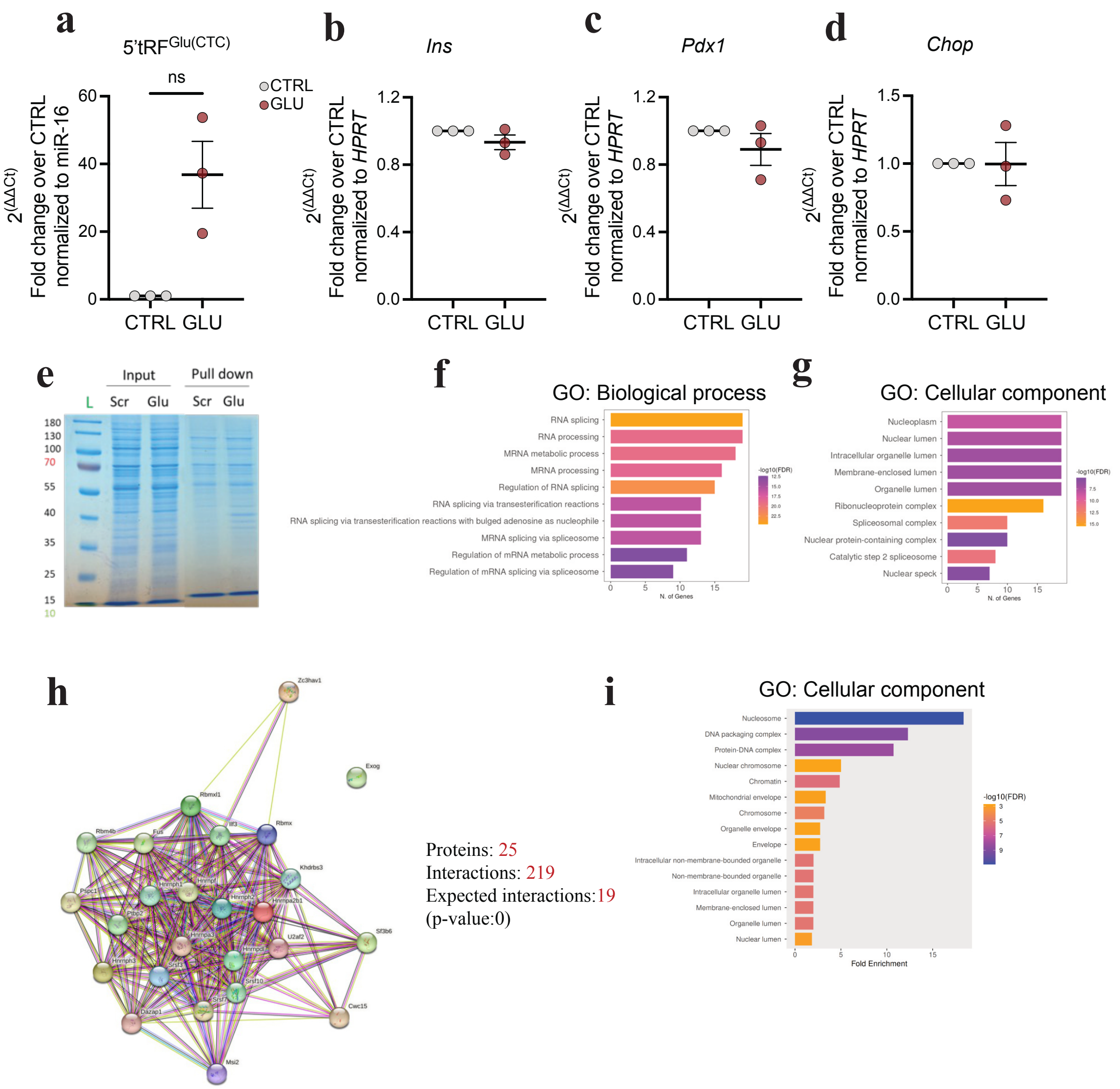

Figure S6: The 5'tRF$^{Glu(CTC)}$ (GLU-red dots) or scrambled (CTRL-grey dots) biotinylated mimics were transfected in MIN6 β-cells; the transfection efficiency was assessed by measuring the levels of 5'tRF$^{Glu(CTC)}$ by qPCR (**a**, n=3 independent experiments). The levels of βcell identity markers *Ins* and *Pdx1*, as well as ER-stress marker *Chop* were assessed by qPCR in GLU (red dots) or CTRL (grey dots) transfected cells (**b-d**, n=3 independent experiments). Data are presented as mean values +/- SEM. Student t-test was applied to evaluate statistical significance. Source data

are provided as a Source Data file. Polyacrylamide gel staining shows the proteins in the input or eluted samples of pull-down experiment (**e**); the experiment was performed twice to set up the best pull-down conditions for mass spectrometry analysis. Overrepresentation test of GO biological processes (**f**) and GO cellular component (**g**) of proteins pulled-down with GLU mimic. **h**) protein-protein interaction analysis using STRING database of proteins pulled-down with GLU mimic. Overrepresentation test of GO cellular component (**i**) of proteins pulled-down with GLU mimic from iMAC-like cells upon PA treatment.

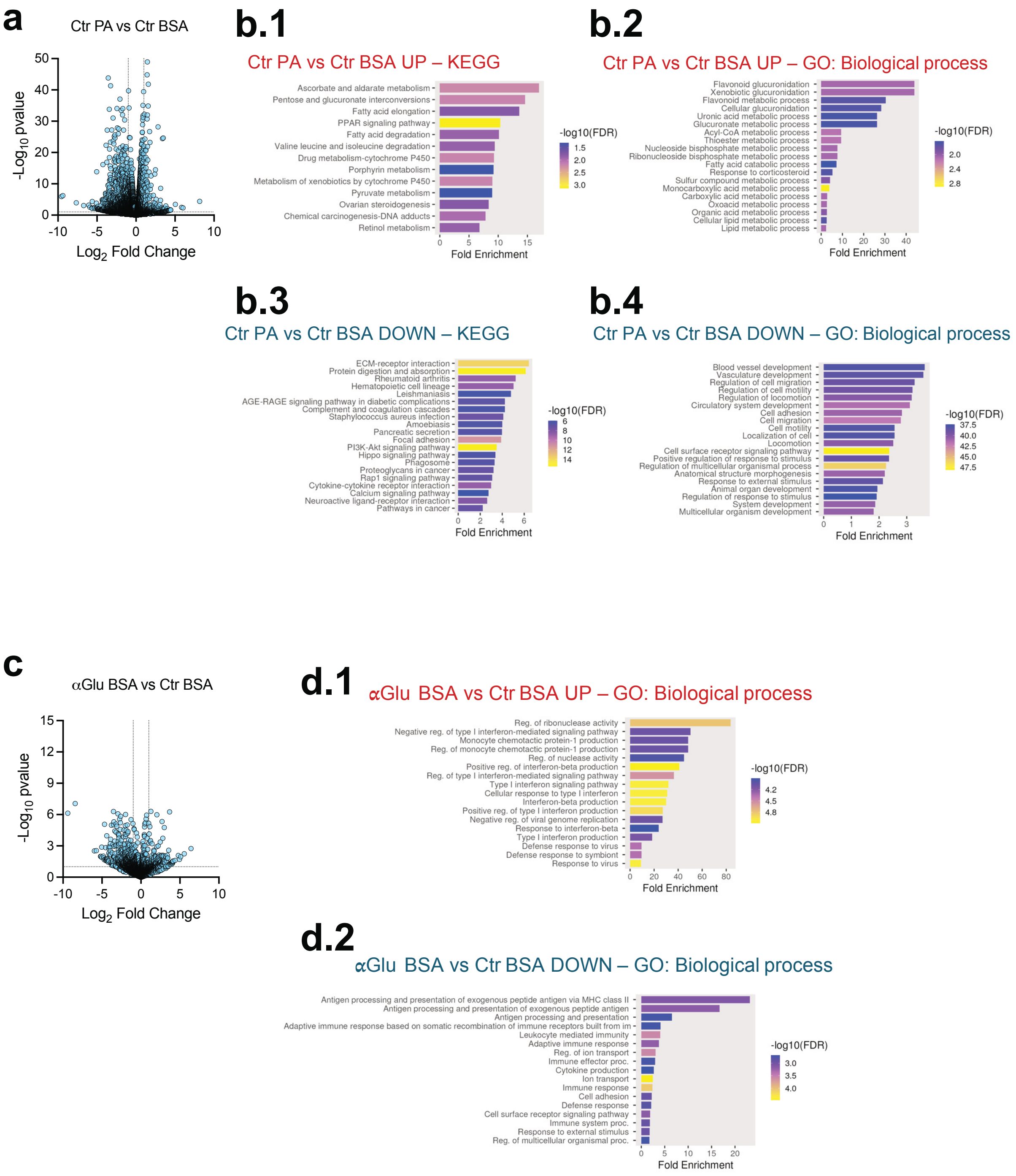

Figure S7: Differential mRNA expression analysis in mouse islets. Volcano plot showing the comparison Palmitate (PA) versus BSA in Ctr transfected cells (**a**, n=6 independent experiments). Differential expression was computed using a negative binomial generalized linear model with two-sided tests and Benjamini–Hochberg FDR correction. Functional enrichment analysis of gene

expression modulation due to PA treatment: bar plots show KEGG and GO biological process terms enriched in upregulated (**b.1-2**) and downregulated genes (**b.3-4**). Volcano plot showing the comparison αGlu versus Ctr transfection in cells kept in basal BSA conditions (**c**, n=6 independent experiments). Functional enrichment analysis of gene expression modulation due to **α**Glu transfection in basal condition: bar plots show GO biological process terms enriched in upregulated (**d.1**) and downregulated (**d.2**) genes.

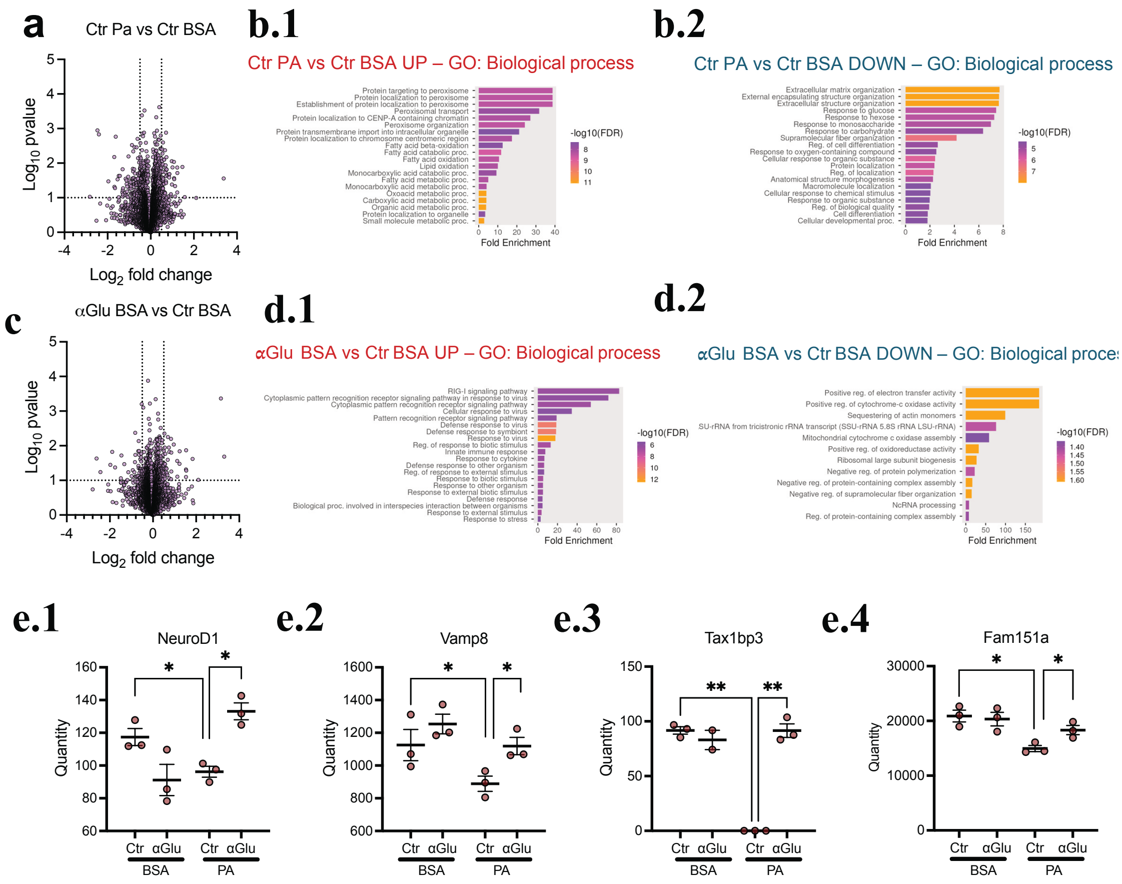

Figure S8: Differential protein expression analysis in mouse islets. Volcano plot showing the comparison Palmitate (PA) versus BSA in Ctr transfected cells (**a**, n=3 independent experiments). Functional enrichment analysis of protein modulation due to PA treatment: bar plots show GO biological process terms enriched in upregulated (**b.1**) and downregulated proteins (**b.2**). Volcano plot showing the comparison αGlu versus Ctr transfection in cells kept in basal BSA conditions (**c**, n=3 independent experiments). Functional enrichment analysis of protein modulation due to **α**Glu transfection in basal condition: bar plots show GO biological process terms enriched in upregulated (**d.1**) and downregulated (**d.2**) proteins. Differential expression analysis (a and c) was

computed using a negative binomial generalized linear model with two-sided t-test. Quantity values of selected proteins from mass spectrometry data showing the modulation in the different conditions (**e.1-4**). Data are presented as mean values +/- SEM. *$p<0.05$, **$p<0.01$, by One-way ANOVA with Sidak correction for multiple comparisons. Exact p values: e.1) p=0.0451 Ctr PA vs Ctr BSA, p=0.0675 **α**Glu PA vs Ctr BSA; e.2) p=0.0283 Ctr PA vs Ctr BSA, p=0.0487 **α**Glu PA vs Ctr BSA; e.3) p=0.0027 Ctr PA vs Ctr BSA, p=0.0092 **α**Glu PA vs Ctr BSA; e.4) p=0.0049 Ctr PA vs Ctr BSA, p=0.0390 **α**Glu PA vs Ctr BSA.

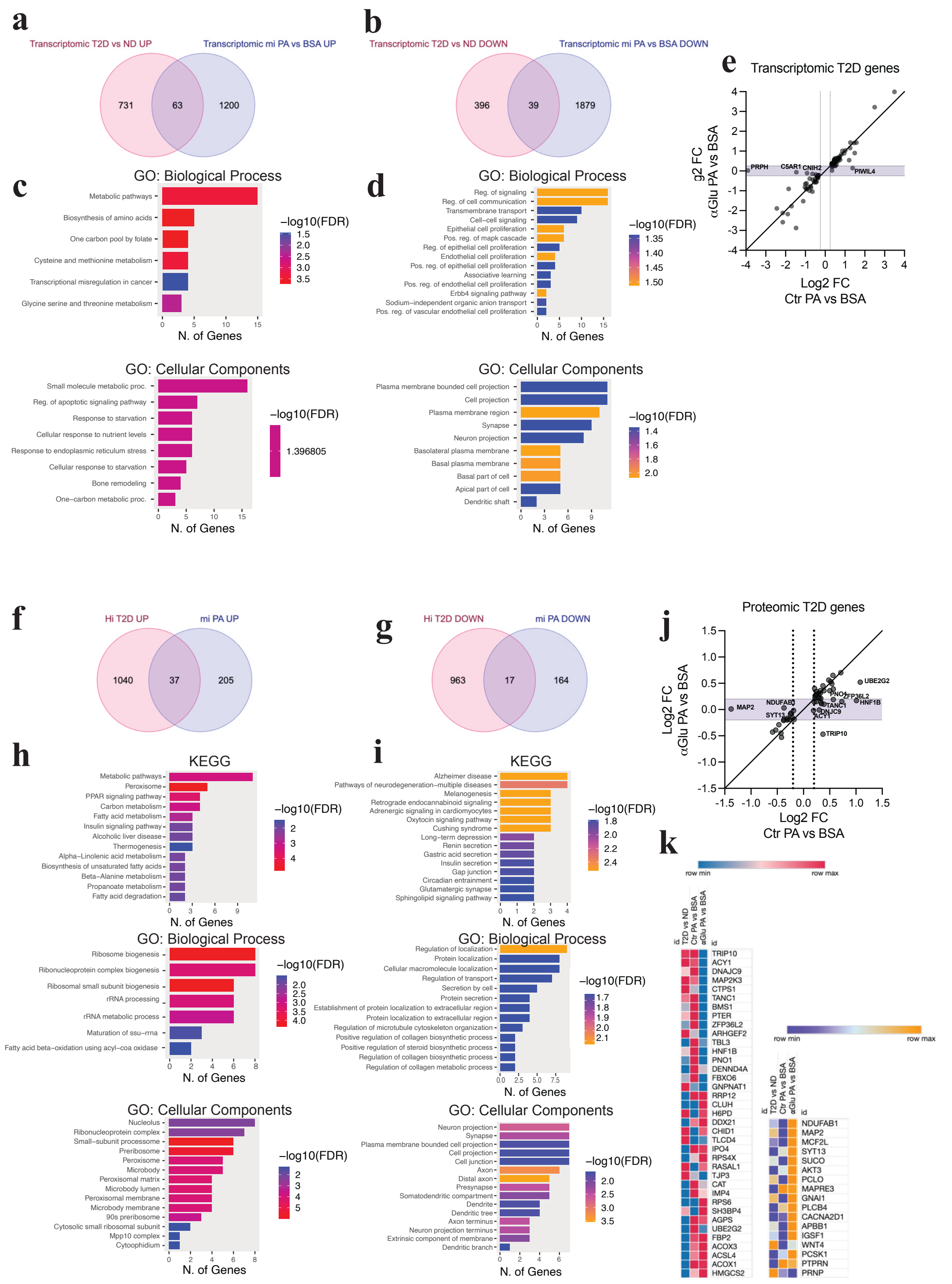

Figure S9: Comparison of gene and protein changes induced by PA in mouse islets and changes observed in type 2 diabetes (T2D) patient islets compared to non-diabetic (ND). Overlap of genes commonly up-regulated (**a**) or downregulated (**b**) in T2D human islets and PA-treated mouse

islets. Functional enrichment of commonly upregulated (**c**) and downregulated (**d**) genes. Fold change correlation of T2D-related genes comparing PA-induced changes in Ctr and in αGlu cells (**e**). Overlap of proteins commonly up-regulated (**f**) or downregulated (**g**) in T2D human islets and PA-treated mouse islets. Functional enrichment of commonly upregulated (**h**) and downregulated (**i**) genes. Fold change correlation of T2D-related genes comparing PA-induced changes in Ctr and in αGlu cells (**j**). Heatmap of Log2 Fold Change values in the different comparisons (T2D vs ND, PA vs BSA in Ctr, PA vs BSA in αGlu cells) (**k**).

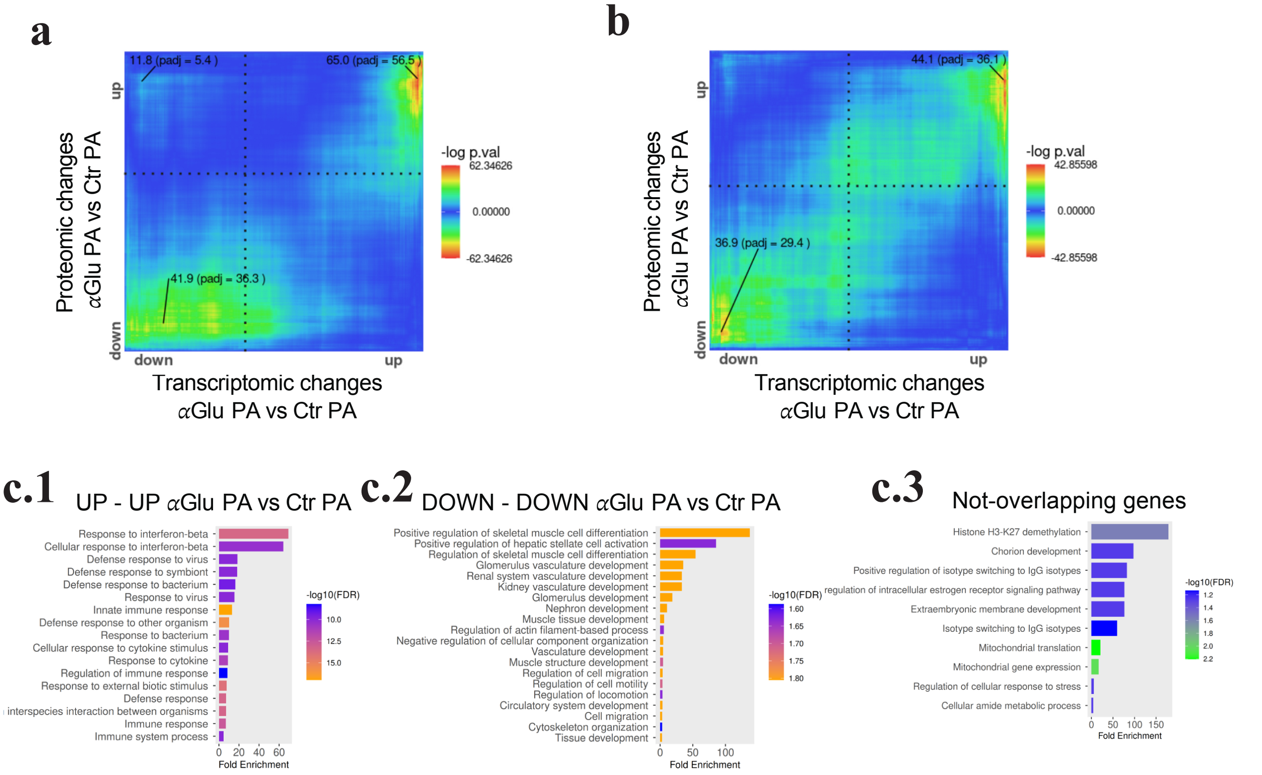

Figure S10: Rank-Rank hypergeometric overlap of transcriptomic and proteomic changes induced by the inhibition of 5’tRF$^{Glu(CTC)}$ in cells in basal BSA conditions (**a**) and PA stimulation (**b**). Functional enrichment of genes whose transcripts and proteins are commonly upregulated (**c.1**) or downregulated (**c.2**) by 5’tRF$^{Glu(CTC)}$ inhibition in cells treated with PA. Functional enrichment of the subset of genes that show differential changes in transcripts and proteins (**c.3**).

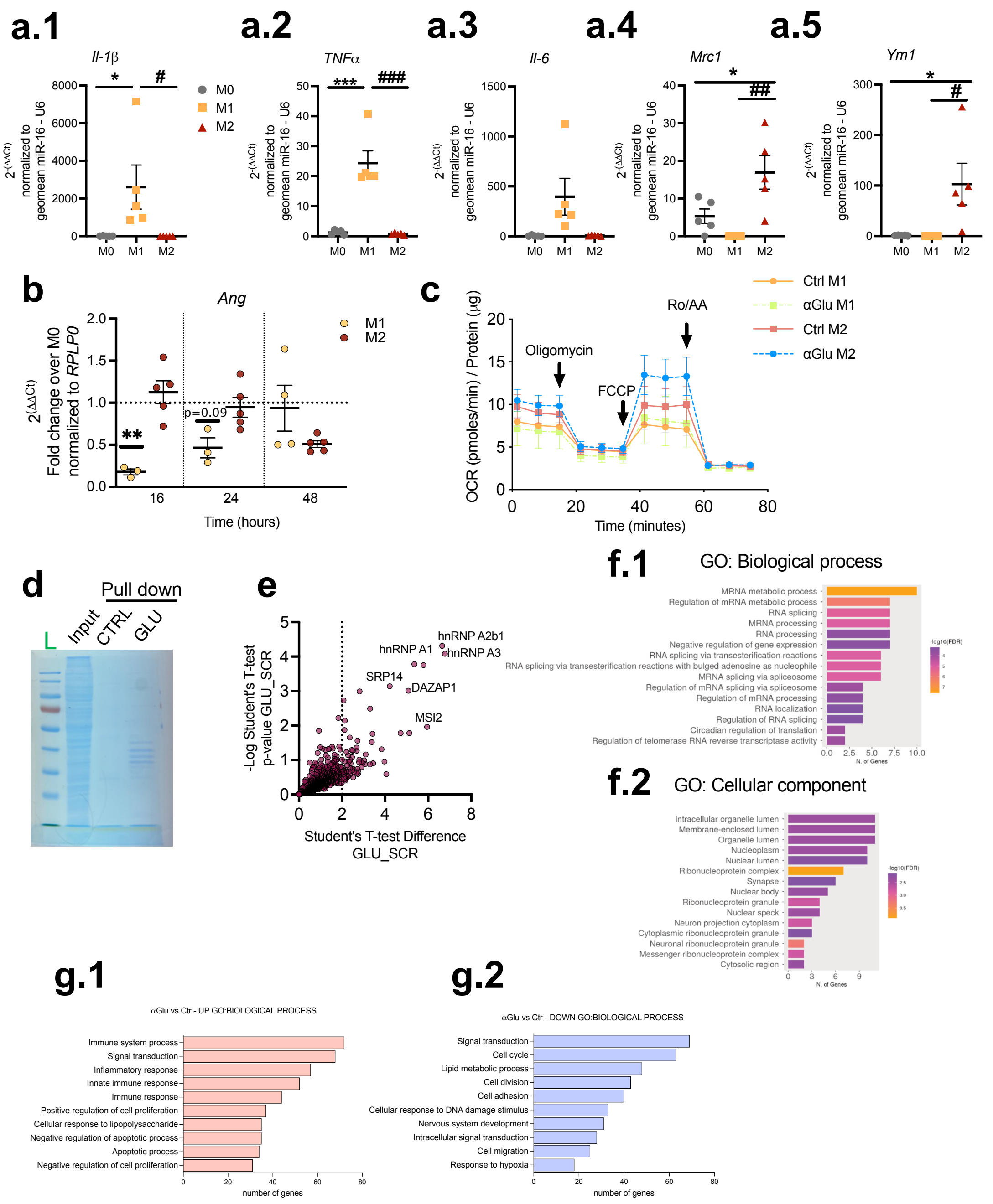

Figure S11: M1 pro-inflammatory and M2 anti-inflammatory polarization was assessed by the expression of gene markers by qPCR (**a1-a5,** n=5 independent experiments). *p<0.05, ***p<0.001, M1 or M2 vs M0, #p<0.05, ##p<0.01, ###p<0.001, M2 vs M1 by One-way ANOVA with Tukey correction for multiple comparisons. In a.1) p=0.0454 M1 vs M0, p=0.0450 M2 vs M1; in a.2) p=0.000046 M1 vs M0, p=0.000038 M2 vs M1; in a.4) p=0.0301 M2 vs M0, p=0.0028 M2 vs M1; in a.5) p=0.0266 M2 vs M0, p=0.0250 M2 vs M1. *Ang* expression was assessed in a

time course polarization in M1 and M2 macrophages by qPCR (**b**, independent experiments: n=3 for M1 16 and 24h, n=4 for M1 48h, n=5 for M2 time course), **p<0.01, p=0.0055 M1 16h vs M0 by One-way ANOVA with Sidak correction for multiple comparisons. OCR was measured in M1 and M2 BMDMs upon transfection with Ctr or αGlu ASO (**c**, n=4 independent experiments). Data are presented as mean values +/- SEM. Source data are provided as a Source Data file. M2-BMDMs lysates were incubated with biotinylated 5'tRF$^{Glu(CTC)}$ mimic (GLU) or with scrambled oligo (CTRL) and then used for pull-down on streptavidin beads. Staining of polyacrylamide gel revealed protein bands present the input and in the eluate of pull-down and from M2 macrophages lysates (**d**). Proteins enriched in the GLU mimic compared to CTRL (**e**) were used for functional enrichment analysis (**f1-f2**). Functional enrichment was performed on upregulated (**g1**) and downregulated (**g2**) genes of M2 macrophages transfected with **α**Glu compared to Ctr.

Supplementary tables

Subject characteristics	NGT (n = 12)	IGT (n = 13)	T2D (n = 11)	p-value
Age (y)	62.3 ± 2.99	65.7 ± 3.74	72.7 ± 2.08	0.08
Sex assigned at birth (F/M)	46179	46209	46089	0.38
BMI (kg/m2)	25.5 ± 1.03	26.0 ± 1.03	26.0 ± 1.11	0.38
Fasting glucose (mmol/L)	5.06 ± 0.16	5.23 ± 0.13	6.69 ± 0.48	3.61E-07
Medium glucose (mmol/L)	7.41 ± 0.24	8.98 ± 0.41	12.7 ± 0.85	3.61E-07
2-h OGTT Glucose (mmol/L)	5.86 ± 0.42	8.86 ± 0.25	15.2 ± 1.19	5.93E-06
Fasting insulin (µUI/ml)	6.39 ± 0.95	9.51 ± 1.34	15.0 ± 4.92	0.13
Basal ISR	56.9 ± 6.73	82.1 ± 8.46	99.4 ± 10.9	0.006
Total ISR	40.1 ± 5.00	54.5 ± 7.52	40.2 ± 4.82	0.16
Glucose Sensitivity	86.1 ± 11.8	94.1 ± 0.4	40.3 ± 8.44	0.002
Rate Sensitivity	1050 ± 290.2	656.1 ± 215.1	228.3 ± 107.4	0.04
Matsuda Index	7.91 ± 1.45	4.06 ± 0.56	4.73 ± 1.26	0.05

Table S1 Subject characteristics and clinical parameters of living organ donors. Data are presented as mean ± standard deviation (SD). Differences among the three study groups (normal glucose tolerance [NGT], impaired glucose tolerance [IGT], and type 2 diabetes [T2D]) were evaluated using a two-sided one-way analysis of variance (ANOVA), with Bonferroni correction for multiple comparisons; sex distribution was compared using the χ^2 test. Samples from living organ donors were used for experiments summarized in Figure 1.h-k and results reported in Supplementary data 1.

Sequence	hit	Clinical parameter	partial_R	p value
TCCCTGGTGGTCTAGTGGTTAGGATTCGG	5'tRF-Glu-CTC	total ISR	-0.48	0.0039
TCCCTGGTGGTCTAGTGGTTAGGATTCGG	5'tRF-Glu-CTC	basal ISR	-0.47	0.0055
TCCCTGGTGGTCTAGTGGTTAGGATTCGG	5'tRF-Glu-CTC	Glucose sensitivity	-0.37	0.0309
TCCCTGGTGGTCTAGTGGTTAGGATTCGG	5'tRF-Glu-CTC	Mean glucose	0.07	0.7068
TCCCTGGTGGTCTAGTGGTTAGGATTCGG	5'tRF-Glu-CTC	Basal glucose	-0.04	0.8317

Table S2 Correlation analysis of 5'tRF$^{Glu(CTC)}$ levels and clinical parameters. The association of Log_2 scaled tRF normalized counts with clinical/metabolic parameters was corrected for the covariates (Age, Sex, BMI). Statistical significance of the association was assessed using two-sided t-tests on the regression coefficients, and regressions with p-values < 0.05 were considered statistically significant.

Data set	tRNA annotation	Sequence	Lenght	Fold change	p value
db/db vs wt - iMAC	tRNA-Glu-CTC	TCCCTGGTGGTCTAGTGGTTAGGATTCGGC**	30	4.23	0.00041
db/db vs wt - βcells	tRNA-Glu-CTC	**CCTGGTGGTCTAGTGGTTAGGATTCGGCGC	30	10.40	0.00007
IGT vs NGT	tRNA-Glu-CTC	TCCCTGGTGGTCTAGTGGTTAGGATTCGG**	29	1.92	0.02355
T2D vs NGT	tRNA-Glu-CTC	TCCCTGGTGGTCTAGTGGTTAGGATTCGG**	29	3.10	0.00096
db/db vs wt - iMAC	tRNA-Gly-GCC	GCATGGGTGGTTCAGTGGTAGAATTCTCGC*	30	3.55	0.00157
db/db vs wt - βcells	tRNA-Gly-GCC	GCATGGGTGGTTCAGTGGTAGAATTC*****	26	3.36	0.00001
T2D vs NGT	tRNA-Gly-GCC	GCATGGGTGGTTCAGTGGTAGAATTCTC***	27	2.23	0.00195

*represents missing nucleotide compared to the most represented sequence in MINTbase v2.0 database, nucleotides in red are part of the anticodon loop
Most represented tRNA half from tRNA-Glu-CTC in MINTbase:tRF-32-87R8WP9N1EWJM (sequence: TCCCTGGTGGTCTAGTGGTTAGGATTCGGCGC)
Most represented tRNA half from tRNA-Gly-GCC in MINTbase: tRF-31-P4R8YP9LON4VD(sequence: GCATGGGTGGTTCAGTGGTAGAATTCTCGCC)

Table S3 Nucleotide sequence of fragments corresponding to the 5'tRF$^{Glu(CTC)}$ in the different datasets. p values were derived from a negative binomial generalized linear model and adjusted for multiple testing using the Benjamini–Hochberg FDR method (two-sided tests).

Pasted	Symbol	Ensembl Gene ID	Chr	Description
NPL	Npl	ENSMUSG00000042684	1	N-acetylneuraminate pyruvate lyase [Source:MGI Symbol;Acc:MGI:1921341]
KYNU	Kynu	ENSMUSG00000026866	2	kynureninase [Source:MGI Symbol;Acc:MGI:1918039]
BOLA1	Bola1	ENSMUSG00000015943	3	bolA-like 1 (E. coli) [Source:MGI Symbol;Acc:MGI:1916418]
APTX	Aptx	ENSMUSG00000028411	4	aprataxin [Source:MGI Symbol;Acc:MGI:1913658]
CER1	Cer1	ENSMUSG00000038192	4	cerberus 1, DAN family BMP antagonist [Source:MGI Symbol;Acc:MGI:1201414]
TRNAU1AP	Trnau1ap	ENSMUSG00000028898	4	tRNA selenocysteine 1 associated protein 1 [Source:MGI Symbol;Acc:MGI:1919037]
MRPL20	Mrpl20	ENSMUSG00000029066	4	mitochondrial ribosomal protein L20 [Source:MGI Symbol;Acc:MGI:2137221]
KCNH2	Kcnh2	ENSMUSG00000038319	5	potassium voltage-gated channel, subfamily H (eag-related), member 2 [Source:MGI Symbol;Acc:MGI:1341722]
HSPB1	Hspb1	ENSMUSG00000004951	5	heat shock protein 1 [Source:MGI Symbol;Acc:MGI:96240]
MTIF3	Mtif3	ENSMUSG00000016510	5	mitochondrial translational initiation factor 3 [Source:MGI Symbol;Acc:MGI:1923616]
PDX1	Pdx1	ENSMUSG00000029644	5	pancreatic and duodenal homeobox 1 [Source:MGI Symbol;Acc:MGI:102851]
HERC6	Herc6	ENSMUSG00000029798	6	hect domain and RLD 6 [Source:MGI Symbol;Acc:MGI:1914388]
MRPL53	Mrpl53	ENSMUSG00000030037	6	mitochondrial ribosomal protein L53 [Source:MGI Symbol;Acc:MGI:1915749]
MRPS35	Mrps35	ENSMUSG00000040112	6	mitochondrial ribosomal protein S35 [Source:MGI Symbol;Acc:MGI:2385255]
PPFIA3	Ppfia3	ENSMUSG00000003863	7	protein tyrosine phosphatase, receptor type, f polypeptide (PTPRF), interacting protein (liprin), alpha 3 [Source:MGI Symbol;Acc:MGI:1924037]
PEX11A	Pex11a	ENSMUSG00000030545	7	peroxisomal biogenesis factor 11 alpha [Source:MGI Symbol;Acc:MGI:1338788]
PAGR1A	Pagr1a	ENSMUSG00000030680	7	PAXIP1 associated glutamate rich protein 1A [Source:MGI Symbol;Acc:MGI:1914528]
PAGR1A	Gm42742	ENSMUSG00000107068	7	predicted gene 42742 [Source:MGI Symbol;Acc:MGI:5662879]
FBXL8	Fbxl8	ENSMUSG00000033313	8	F-box and leucine-rich repeat protein 8 [Source:MGI Symbol;Acc:MGI:1354697]
MARVELD3	Marveld3	ENSMUSG00000001672	8	MARVEL (membrane-associating) domain containing 3 [Source:MGI Symbol;Acc:MGI:1920858]
BANP	Banp	ENSMUSG00000025316	8	BTG3 associated nuclear protein [Source:MGI Symbol;Acc:MGI:1889023]
RPUSD4	Rpusd4	ENSMUSG00000032044	9	RNA pseudouridylate synthase domain containing 4 [Source:MGI Symbol;Acc:MGI:1919239]
CYB561D2	Cyb561d2	ENSMUSG00000037190	9	cytochrome b-561 domain containing 2 [Source:MGI Symbol;Acc:MGI:1929280]
UBE2G2	Ube2g2	ENSMUSG00000009293	10	ubiquitin-conjugating enzyme E2G 2 [Source:MGI Symbol;Acc:MGI:1343188]
TPGS1	Tpgs1	ENSMUSG00000020308	10	tubulin polyglutamylase complex subunit 1 [Source:MGI Symbol;Acc:MGI:106618]
PALM	Palm	ENSMUSG00000035863	10	paralemmin [Source:MGI Symbol;Acc:MGI:1261814]
REEP6	Reep6	ENSMUSG00000035504	10	receptor accessory protein 6 [Source:MGI Symbol;Acc:MGI:1917585]
TIMM13	Timm13	ENSMUSG00000020219	10	translocase of inner mitochondrial membrane 13 [Source:MGI Symbol;Acc:MGI:1353432]
SIRT6	Sirt6	ENSMUSG00000034748	10	sirtuin 6 [Source:MGI Symbol;Acc:MGI:1354161]
MRM3	Mrm3	ENSMUSG00000038046	11	mitochondrial rRNA methyltransferase 3 [Source:MGI Symbol;Acc:MGI:1914640]
ALDOC	Aldoc	ENSMUSG00000017390	11	aldolase C, fructose-bisphosphate [Source:MGI Symbol;Acc:MGI:101863]
CCT6B	Cct6b	ENSMUSG00000020698	11	chaperonin containing Tcp1, subunit 6b (zeta) [Source:MGI Symbol;Acc:MGI:1329013]
AATK	Aatk	ENSMUSG00000025375	11	apoptosis-associated tyrosine kinase [Source:MGI Symbol;Acc:MGI:1197518]
AGR2	Agr2	ENSMUSG00000020581	12	anterior gradient 2 [Source:MGI Symbol;Acc:MGI:1344405]
ABCD4	Abcd4	ENSMUSG00000021240	12	ATP-binding cassette, sub-family D (ALD), member 4 [Source:MGI Symbol;Acc:MGI:1349217]
COMTD1	Comtd1	ENSMUSG00000021773	14	catechol-O-methyltransferase domain containing 1 [Source:MGI Symbol;Acc:MGI:1916406]
SNCG	Sncg	ENSMUSG00000023064	14	synuclein, gamma [Source:MGI Symbol;Acc:MGI:1298397]
MYLK	Mylk	ENSMUSG00000022836	16	myosin, light polypeptide kinase [Source:MGI Symbol;Acc:MGI:894806]
RFX2	Rfx2	ENSMUSG00000024206	17	regulatory factor X, 2 (influences HLA class II expression) [Source:MGI Symbol;Acc:MGI:106583]
XDH	Xdh	ENSMUSG00000024066	17	xanthine dehydrogenase [Source:MGI Symbol;Acc:MGI:98973]
GEMIN8	Gemin8	ENSMUSG00000040621	X	gem nuclear organelle associated protein 8 [Source:MGI Symbol;Acc:MGI:2384300]

Table S4 Not overlapping genes from Rank-Rank hypergeometric analysis of proteomic and transcriptomic in islet cells upon 5'tRF$^{Glu(CTC)}$ inhibition and palmitate treatment.

Preparation	Arrival date	Age	Sex assigned at birth	Weight (Kg)	Heght (cm)	Cause of death	Viability	Purity
HP2301	17.01.23	66	M	90	175	Stroke	80%	>70%
HP2302	26.01.23	51	M	79	173	Traumatic injury	80-90%	>70%
HP2303	07.02.23	71	M	101	172	Traumatic injury	90%	90%
HP2308	22.06.23	62	F	71.7	160	Aneurysm rupture	90%	>70%
HP2405	30.05.24	78	F	77.2	164	Stroke	95%	92%
HP2408	23.10.24	59	F	103	163	Stroke	90%	
HP2409	13.11.24	46	M	94,5	184	Stroke	90%	65%
HP2502	25.03.25	51	F	138.5	169	Resuscitated cardiac arrest	80-90%%	80%%
HP2512	17.11.25	52	F	58.6	150	Resuscitated cardiac arrest	90%	90%

Table S5 Characteristics of cadaveric human islet preparations used for results in Figure 2.c, Figure 3.g-j, m-n, Figure S4.e and g.

Antibodies, reagents and kits			
**Name**	**Company**	**Reference**	**Application**
FITC anti-mouse CD45 Antibody	BioLegend	103108	FACS
Brilliant Violet 421™ anti-mouse/human CD11b Antibody	BioLegend	101236	FACS
APC anti-mouse F4/80 Antibody	BioLegend	123116	FACS
PE anti-mouse CD11c Antibody	BioLegend	117308	FACS
INS Monoclonal antibody	Proteintech	66198-1-Ig	Immunofluorescence
Cleaved Caspase-3 (Asp175)	Cell Signaling	9661	Immunofluorescence
Goat anti-Rabbit Alexa Fluor™ 568	ThermoFisher Scientific	A-11011	Immunofluorescence
Goat anti-Mouse Alexa Fluor™ 488	ThermoFisher Scientific	A-11001	Immunofluorescence
MSI2 Polyclonal antibody	Proteintech	10770-1-AP	Western blot
HNRNPA3 Polyclonal antibody	Proteintech	25142-1-AP	Western blot
Goat Anti-Rabbit IgG (H + L)-HRP Conjugate	Biorad	1706515	Western blot
Goat Anti-Mouse IgG (H + L)-HRP Conjugate	Biorad	1706516	Western blot
Oleic acid	Sigma - Aldrich	O1383-1G	Cell treatment
Stearic acid	Sigma - Aldrich	S4751-1G	Cell treatment
Palmitic acid	Sigma - Aldrich	P5585	Cell treatment
Albumin, Bovine Serum, Fraction V, Fatty Acid Free	Sigma - Aldrich	126575-10GM	Cell treatment
Murine IL-4	Peprotech	214-14	Cell treatment
Murine IL-13	Peprotech	210-13	Cell treatment
Recombinant Murine IFN-γ	Peprotech	315-05	Cell treatment
Murine M-CSF	Peprotech	315-02	Cell treatment
Lipopolysaccharid aus Escherichia coli O111:B4	Sigma - Aldrich	L2630-10MG	Cell treatment
Insulin ELISA	Mercodia	10-1113-01	Insulin quantification
Mouse Insulin ELISA	Mercodia	10-1247-10	Insulin quantification
AHARIBO RNA System_v2	Immagina Biotechnology	#AHA-RM12	Tranlsatomic
Seahorse XFp Mito Stress Test kit	Agilent	103010-100	Mitochondrial function

Table S6 Antibodies, reagents and kits used in the study.

Extended material and methods

RNA pull-down in MIN6 cells

Beads were resuspended in SP3 buffer (2% SDS, 10mM DTT, 50 mM Tris, pH 7.5) and heated 10 min at 75°C to elute proteins. Eluates were digested following the SP3 method[1] using magnetic Sera-Mag Speedbeads (Cytiva 45152105050250, 50 mg/ml). Briefly, samples in SP3 buffer were first treated with 32mM (final) iodoacetamide for 45 min at RT in the dark to alkylate reduced cysteines. Beads were then added at a ratio 10:1 (w:w) to samples, and proteins were precipitated on beads with ethanol (final concentration: 60 %). After 3 washes with 80% ethanol, beads were digested in 50ul of 100 mM ammonium bicarbonate with 1.0 ug of trypsin (Promega #V5113). After 1h of incubation at 37°C, the same amount of trypsin was added to the samples for an additional 1h of incubation. Supernatant were then recovered and transferred to new tubes. Two sample volumes of isopropanol containing 1% TFA were added to the digests, and the samples were desalted on a strong cation exchange (SCX) plate (Oasis MCX; Waters Corp., Milford, MA) by centrifugation to remove traces of SDS. After washing with isopropanol/1%TFA and 2% acetonitrile/0.1% FA, peptides were eluted in 200ul of 40% MeCN, 59% water, 1% (v/v) ammonia, and dried by centrifugal evaporation.

LC-MS analysis

Tryptic peptide mixtures were injected on a Vanquish Neo nanoHPLC system interfaced via a nanospray Flex source to a high resolution Orbitrap Exploris 480 mass spectrometer (Thermo Fisher, Bremen, Germany). Peptides were loaded onto a trapping microcolumn PepMap100 C18 (5 mm x 1.0 mm ID, 5 μm, Thermo Fisher) before separation on a C18 custom packed column (75 μm ID × 45 cm, 1.8 μm particles, Reprosil Pur, Dr. Maisch), using a gradient from 2 to 80 % acetonitrile in 0.1 % formic acid for peptide separation at a flow rate of 250 nl/min (total time: 130 min). Full MS survey scans were performed at 120,000 resolution. A data-dependent acquisition method controlled by Xcalibur software (Thermo Fisher Scientific) was used that optimized the number of precursors selected ("top speed") of charge 2+ to 5+ while maintaining a fixed scan cycle of 2 s. Peptides were fragmented by higher energy collision dissociation (HCD) with a normalized energy of 30 % at 15'000 resolution. The window for precursor isolation was of 1.6 m/z units around the precursor and selected fragments were excluded for 60s from further analysis.

RNA pull-down in iMAC and BMDMs

Proteins on beads were digested following a modified version of the iST method[2] (named miST method). 25 ul of miST lysis buffer (1% Sodium deoxycholate, 100mM Tris pH 8.6, 10 mM DTT), were added to the beads. After mixing and dilution 1:1 (v:v) with H2O, samples were heated 5 min at 75°C. After digestion with 0.5 ug of Trypsin/LysC mix (Promega #V5073) for 1h at 25°C, sample supernatants were transferred in new tubes. Beads were washed with 50 ul of miST buffer diluted 1/1 in H2O, and supernatants pooled with the previous ones. Reduced disulfides were alkylated by adding 25 ul of 160 mM chloroacetamide (32 mM final) and incubating for 45min at 25°C in the dark. Samples were then digested overnight at 25°C with 1.0 ug Trypsin/LysC mix. To remove sodium deoxycholate, two sample volumes of isopropanol containing 1% TFA were added to the digests, and the samples were desalted on a strong cation exchange (SCX) plate (Oasis MCX; Waters Corp., Milford, MA) by centrifugation. After washing with isopropanol/1%TFA, peptides were eluted in 200ul of 80% MeCN, 19% water, 1% (v/v) ammonia, and dried by centrifugal evaporation.

LC-MS

Data-dependent LC-MS/MS analyses of samples were carried out on a Fusion Tribrid Orbitrap mass spectrometer (Thermo Fisher Scientific) interfaced through a nano-electrospray ion source to an Ultimate 3000 RSLCnano HPLC system (Dionex). Peptides were separated on a reversed-phase custom packed 45 cm C18 column (75 µm ID, 100Å, Reprosil Pur 1.9 um particles, Dr. Maisch, Germany) with a 4-90% acetonitrile gradient in 0.1% formic acid at a flow rate of 250 nl/min (total time 140 min). Full MS survey scans were performed at 120'000 resolution. A data-dependent acquisition method controlled by Xcalibur software (Thermo Fisher Scientific) was used that optimized the number of precursors selected ("top speed") of charge 2^+ to 5^+ while maintaining a fixed scan cycle of 0.6 s. Peptides were fragmented by higher energy collision dissociation (HCD) with a normalized energy of 32%. The precursor isolation window used was 1.6 Th, and the MS2 scans were done in the ion trap. The *m/z* of fragmented precursors was then dynamically excluded from selection during 60 s.

LC-MS data annotation and analysis

Data files were analysed with MaxQuant 2.4.7.0[3] incorporating the Andromeda search engine[4]. Cysteine carbamidomethylation was selected as fixed modification while methionine oxidation and protein N-terminal acetylation were specified as variable modifications. The sequence databases used for searching were the mouse (*Mus musculus*) reference proteome based on the

UniProt database (www.uniprot.org, RefProt_Mus_musculus_20230301, containing 55'309 sequences), and a "contaminant" database containing the most usual environmental contaminants and enzymes used for digestion (keratins, trypsin, etc)[5]. Mass tolerance was 4.5 ppm on precursors (after recalibration) and 20 ppm on MS/MS fragments. Both peptide and protein identifications were filtered at 1% FDR relative to hits against a decoy database built by reversing protein sequences.

All subsequent analyses were done with an in house developed software tool (available on https://github.com/UNIL-PAF/taram-backend/). Contaminant proteins were removed, and iBAQ values[6] for protein groups were log2-transformed. After assignment to groups, only proteins quantified in at least 4/5 samples of one group were kept. Missing values were imputed based on a normal distribution with a width of 0.3 standard deviations (SD), down-shifted by 1.8 SD relative to the median. Student's T-tests were carried out among conditions, with Benjamini-Hochberg correction for multiple testing (adjusted p-value threshold <0.05). Imputed values were later removed.

Proteomic analysis of mouse islets

Cell lysates in RIPA buffer were digested following the SP3 method[1] using magnetic Sera-Mag Speedbeads (Cytiva 45152105050250, 50 mg/ml). Briefly, samples were diluted with SP3 buffer (2% SDS, 10mM DTT, 50 mM Tris, pH 7.5) and heated 10 min at 75°C. Proteins were then alkylated with 32mM (final) iodoacetamide for 45 min at RT in the dark. Beads were added at a ratio 10:1 (w:w) to samples, and proteins were precipitated on beads with ethanol (final concentration: 60 %). After 3 washes with 80% ethanol, beads were digested in 50ul of 100 mM ammonium bicarbonate with 1.0 ug of trypsin (Promega #V5113). After 1h of incubation at 37°C, the same amount of trypsin was added to the samples for an additional 1h of incubation. Supernatant were then recovered and transferred to new tubes. Two sample volumes of isopropanol containing 1% TFA were added to the digests, and the samples were desalted on a strong cation exchange (SCX) plate (Oasis MCX; Waters Corp., Milford, MA) by centrifugation to remove traces of SDS. After washing with isopropanol/1%TFA and 2% acetonitrile/0.1% FA, peptides were eluted in 200ul of 40% MeCN, 59% water, 1% (v/v) ammonia, and dried by centrifugal evaporation.

LC-MS/MS analyses were carried out on a TIMS-TOF Pro (Bruker, Bremen, Germany) mass spectrometer interfaced through a nanospray ion source ("captive spray") to an EvoSep One liquid

chromatography system (EvoSep, Odense, Denmark). Peptides were separated on a reversed-phase Aurora Elite C18 column (15 cm, 75 µm ID, 1.7um, IonOpticks) at a flow rate of 200 nl/min with a 20 sample per day method (runtime time: 68 min, solvents were water and acetonitrile with 0.1% formic acid).

Data-independent acquisition was carried out using a method similar to a standard DIA-PASEF method reported previously[7], with ion accumulation for 100 ms for each the survey MS1 scan and the MS2 scans. Duty cycle was kept at 100%. Precursor ions were chosen within a reduced mobility range from 1/k0 =0.7 to 1.4 and between m/z=400 to 1200. Collision energy was ramped linearly based uniquely on the 1/k0 values from 20 (at 1/k0=0.6) to 59 eV (at 1/k0=1.6). Per cycle, the mass range 400-1200 m/z was covered by a total of 20 windows, each 40 Th wide, with a total cycle time of 1.3 s.

Data processing

Identification of peptides directly from DIA data was performed with Spectronaut 19.9 with the Pulsar engine using the "deep" setting and searching the reference mouse proteome (www.uniprot.org) database of February 6th, 2025 (54'747 sequences), and a contaminant database containing the most usual environmental contaminants and enzymes used for digestion (from Frankenfield, et al., 2022)[5]. For identification, peptides of 7-52 AA length were considered, cleaved with trypsin/P specificity and a maximum of 2 missed cleavages. Carbamidomethylation of cysteine (fixed), methionine oxidation and N-terminal protein acetylation (variable) were the modifications applied. FDR's for peptide and protein group identifications were all at 1%. Ion mobility for peptides was predicted using a deep neural network and used in scoring. The library created contained overall 228,479 precursors.

Peptide-centric analysis of DIA data was done with Spectronaut 19.9 using the library generated by Pulsar from DIA data. Single hits proteins (defined as matched by one stripped sequence only) were kept in the Spectronaut analysis. Peptide quantitation was based on XIC area, for which a minimum of 1 and a maximum of 3 (the 3 best) precursors were considered for each peptide, from which the mean value was selected. Quantities for protein groups were derived from inter-run peptide ratios based on MaxLFQ algorithm[8]. Global normalization of runs/samples was done based on the median of peptides. Overall 216,168 precursors were quantified in the dataset, mapped to 9'527protein groups. 113,406 precursors (7'584 protein groups) had full profiles, i.e. were quantified in all samples. The average number of data points per peak was 6.1.

Data analysis

All subsequent analyses were done with an in house developed software tool (available on https://github.com/UNIL-PAF/taram-backend/). Contaminant proteins were removed, and quantity values form Spectronaut for protein groups were log2-transformed. After assignment to groups, only proteins quantified in at least 3/3 samples of one group were kept. Missing values were imputed based on a normal distribution with a width of 0.3 standard deviations (SD), down-shifted by 1.8 SD relative to the median. Student's T-tests were carried out among conditions, with Benjamini-Hochberg correction for multiple testing (adjusted p-value threshold <0.05). Imputed values were later removed.

Supplementary References

1 Hughes, C. S. *et al.* Single-pot, solid-phase-enhanced sample preparation for proteomics experiments. *Nat Protoc* **14**, 68-85 (2019). https://doi.org/10.1038/s41596-018-0082-x

2 Kulak, N. A., Pichler, G., Paron, I., Nagaraj, N. & Mann, M. Minimal, encapsulated proteomic-sample processing applied to copy-number estimation in eukaryotic cells. *Nature Methods* **11**, 319-324 (2014). https://doi.org/10.1038/nmeth.2834

3 Cox, J. & Mann, M. MaxQuant enables high peptide identification rates, individualized p.p.b.-range mass accuracies and proteome-wide protein quantification. *Nat Biotechnol* **26**, 1367-1372 (2008). https://doi.org/10.1038/nbt.1511

4 Cox, J. *et al.* Andromeda: a peptide search engine integrated into the MaxQuant environment. *J Proteome Res* **10**, 1794-1805 (2011). https://doi.org/10.1021/pr101065j

5 Frankenfield, A. M., Ni, J., Ahmed, M. & Hao, L. Protein Contaminants Matter: Building Universal Protein Contaminant Libraries for DDA and DIA Proteomics. *J Proteome Res* **21**, 2104-2113 (2022). https://doi.org/10.1021/acs.jproteome.2c00145

6 Schwanhäusser, B. *et al.* Global quantification of mammalian gene expression control. *Nature* **473**, 337-342 (2011). https://doi.org/10.1038/nature10098

7 Meier, F. *et al.* diaPASEF: parallel accumulation-serial fragmentation combined with data-independent acquisition. *Nat Methods* **17**, 1229-1236 (2020). https://doi.org/10.1038/s41592-020-00998-0

8 Cox, J. *et al.* Accurate proteome-wide label-free quantification by delayed normalization and maximal peptide ratio extraction, termed MaxLFQ. *Mol Cell Proteomics* **13**, 2513-2526 (2014). https://doi.org/10.1074/mcp.M113.031591
