# Supplementary material for: 5’tRNA-derived fragments modulate β-cell homeostasis and islet macrophage activation in type 2 diabetes
Source: Nat Commun. 2026 May 4;17:5989. doi: 10.1038/s41467-026-72641-z (PMC13346763; doi:10.1038/s41467-026-72641-z)
Supplement: Supplementary file 2 — Description of Additional Supplementary Files [file 41467_2026_72641_MOESM2_ESM.pdf]

Description of Additional Supplementary Files

Title: Supplementary data 1
Description: Processed data of tRF profiling from living donor islet samples. Results derived from these data are shown in Figure 1.h-k. tRNA genomic annotation, raw and normalized counts are provided.

Title: Supplementary data 2
Description: tRF sequences targeted by custom inhibitors and sequences of oligonucleotides used as primers and adapter in the indicated experiments.
