# Supplementary material for: 5’tRNA-derived fragments modulate β-cell homeostasis and islet macrophage activation in type 2 diabetes
Source: Nat Commun. 2026 May 4;17:5989. doi: 10.1038/s41467-026-72641-z (PMC13346763; doi:10.1038/s41467-026-72641-z)
Supplement: Supplementary file 5 — Reporting Summary [file 41467_2026_72641_MOESM5_ESM.pdf]

nature portfolio

Corresponding author(s): Cristina Cosentino

Last updated by author(s): 28/01/2026

Reporting Summary

Nature Portfolio wishes to improve the reproducibility of the work that we publish. This form provides structure for consistency and transparency in reporting. For further information on Nature Portfolio policies, see our Editorial Policies and the Editorial Policy Checklist.

Statistics

For all statistical analyses, confirm that the following items are present in the figure legend, table legend, main text, or Methods section.

n/a	Confirmed	
☐	☒	The exact sample size (*n*) for each experimental group/condition, given as a discrete number and unit of measurement
☐	☒	A statement on whether measurements were taken from distinct samples or whether the same sample was measured repeatedly
☐	☒	The statistical test(s) used AND whether they are one- or two-sided<br>*Only common tests should be described solely by name; describe more complex techniques in the Methods section.*
☐	☒	A description of all covariates tested
☐	☒	A description of any assumptions or corrections, such as tests of normality and adjustment for multiple comparisons
☐	☒	A full description of the statistical parameters including central tendency (e.g. means) or other basic estimates (e.g. regression coefficient) AND variation (e.g. standard deviation) or associated estimates of uncertainty (e.g. confidence intervals)
☐	☒	For null hypothesis testing, the test statistic (e.g. *F*, *t*, *r*) with confidence intervals, effect sizes, degrees of freedom and *P* value noted<br>*Give P values as exact values whenever suitable.*
☒	☐	For Bayesian analysis, information on the choice of priors and Markov chain Monte Carlo settings
☒	☐	For hierarchical and complex designs, identification of the appropriate level for tests and full reporting of outcomes
☒	☐	Estimates of effect sizes (e.g. Cohen's *d*, Pearson's *r*), indicating how they were calculated

Our web collection on statistics for biologists contains articles on many of the points above.

Software and code

Policy information about availability of computer code

Data collection	Flow cytometry data were acquired on a BD FACSAria II cell sorter using BD FACSDiva software (BD Biosciences).
Data analysis	FACS data were analysed using FlowJo v.11 software (BD Biosciences). tRF profiling was conducted on edgeR (version 4.2.2) in R packages (http://bioconductor.org). MAss spectrometry raw data from pull-down experiments were processed with MaxQuant 2.4.7.0. Raw data from proteomic experiments were analyzed with Spectronaut 19.9 using the Pulsar engine. Gene and protein lists were analysed for pathway enrichment using ShinyGO v.0.82 tool70. KEGG and GO enrichments with FDR pvalue<0.1 were considered significant. Rank-rank hypergeometric overlap was performed using the R-package RedRibbon v.1.3.137. Transcriptomic data of M2 macrophages were run on MacSpectrum web tool v.1.0.140.. Statistical analysis was performed with Graphpad Prism 10.

For manuscripts utilizing custom algorithms or software that are central to the research but not yet described in published literature, software must be made available to editors and reviewers. We strongly encourage code deposition in a community repository (e.g. GitHub). See the Nature Portfolio guidelines for submitting code & software for further information.

Data

Policy information about availability of data

All manuscripts must include a data availability statement. This statement should provide the following information, where applicable:

- Accession codes, unique identifiers, or web links for publicly available datasets
- A description of any restrictions on data availability
- For clinical datasets or third party data, please ensure that the statement adheres to our policy

The small RNA sequencing data data generated in this study have been deposited in the GEO database under accession code GSE302055 and GSE302056. The processed data of small RNA sequencing from LCM human islets generated in this study are provided in the Supplementary Data 1 file. The mass spectrometry data derived from pull-down have been deposited in the ProteomeXchange database under accession code PXD065726, PXD065736 and PXD073702. The mRNA sequencing data generated in this study are provided in the GEO database under accession code: GSE302250 and GSE302527. Proteomic data generated in this study are provided in the ProteomeXchange database under accession code PXD065746. The processed data generated in this study from translatomic experiments are in Zenodo repository under DOI 10.5281/zenodo.1897811871. Human islet bulk RNA sequencing and proteomic data were downloaded from humanislets.com database; the raw RNA sequencing data are deposited in the European Genome-phenome Archive (EGA) under accession code EGAS00001007241, while raw proteomics data are deposited to ProteomeXchange via MASSive under accession code PXD045422. Source data are provided with this paper.

Research involving human participants, their data, or biological material

Policy information about studies with human participants or human data. See also policy information about sex, gender (identity/presentation), and sexual orientation and race, ethnicity and racism.

Reporting on sex and gender	Subject characteristics Normal Glucose Tolerant NGT (n = 12) Impaired Glucose Tolerant IGT (n = 13) and Type 2 diabetic T2D (n = 11) . Sex assigned at birth (F/M) is 6/6 in NGT; 6/7 in IGT; 8/3 in T2D. Individual values of clinical parameters and RNA sequencing data can be found in source data for table S1 and Supplementary data 1, respectively.
Reporting on race, ethnicity, or other socially relevant groupings	No socially constructed or socially relevant categories are described or considered in the manuscript.
Population characteristics	Age is 62.3 ± 2.99 in NGT; 65.7 ± 3.74 in IGT; 72.7 ± 2.08 in T2D<br>Body max index (kg/m2) is 25.5 ± 1.03 in NGT; 26.0 ± 1.03 in IGT; 26.0 ± 1.11 in T2D
Recruitment	Inclusion Criteria: Age between 20 and 70 years, Candidates for pancreaticoduodenectomy for periampullary neoplasms, HbA1c < 7.0%, Fasting triglycerides < 200 mg/dL, LDL cholesterol < 160 mg/dL<br><br>Exclusion Criteria: Diagnosis of type 1 diabetes mellitus; Moderate to severe liver disease, including: hepatic steatosis (alcohol-related or non-alcohol-related), liver cirrhosis, hepatitis; Severe renal failure (eGFR < 15 mL/min/1.73 m²); Pregnancy or breastfeeding; Presence of severe concomitant diseases; Inability to provide written informed consent; Treatment with oral antidiabetic drugs.
Ethics oversight	The study protocol (ClinicalTrials.gov NCT02175459) was approved by the Ethical Committee Fondazione Policlinico Universitario Agostino Gemelli IRCCS – Università Cattolica del Sacro Cuore (P/656/CE2010 and 22573/14)

Note that full information on the approval of the study protocol must also be provided in the manuscript.

Field-specific reporting

Please select the one below that is the best fit for your research. If you are not sure, read the appropriate sections before making your selection.

☒ Life sciences ☐ Behavioural & social sciences ☐ Ecological, evolutionary & environmental sciences

For a reference copy of the document with all sections, see nature.com/documents/nr-reporting-summary-flat.pdf

Life sciences study design

All studies must disclose on these points even when the disclosure is negative.

Sample size	No calculation was performed to predetermine sample size. It was based on the quality, functionality, and availability of the samples in order to obtain representative results. The sample sizes were sufficient for the requirements of the respective statistical test.
Data exclusions	No data were excluded.
Replication	Independent experiments including positive and/or negative controls were performed for each analysis. For primary cells, each eperimental replicate constitutes a preparation from a different organism. For cell lines, replications are meant as independent experiments carried on different cell passages and thaw batches. Replication was confirmed. The experiments were replicated or performed independently between 2 and 10 times. The number of independent experiments and a statement on replication is included in figure legends where appropriate.

Randomization	Animal studies included control and diabetic animals matched by age and genetic background. Human studies included impaired glucose tolerant, normal glucose tolerant and type 2 diabetes living organ donors, data were corrected for covariants sex, age and BMI. Human islets for in vitro experiments derived from non-diabetic cadaver donors of random age, sex, and BMI. Randomization was not required for studies using MIN6 or primary mouse islet cells as all experimental groups were obtained from cells of either the same passage or same animal for each biological replicate.
Blinding	Investigators were not blinded to group allocation. Blinding was not relevant since controls were used for the animal and human studies. In addition, most data are supported by data obtained from unbiased methods such as RNA sequencing.

Reporting for specific materials, systems and methods

We require information from authors about some types of materials, experimental systems and methods used in many studies. Here, indicate whether each material, system or method listed is relevant to your study. If you are not sure if a list item applies to your research, read the appropriate section before selecting a response.

Materials & experimental systems

n/a	Involved in the study	
☐	☒	Antibodies
☐	☒	Eukaryotic cell lines
☒	☐	Palaeontology and archaeology
☐	☒	Animals and other organisms
☐	☒	Clinical data
☒	☐	Dual use research of concern
☒	☐	Plants

Methods

n/a	Involved in the study	
☒	☐	ChIP-seq
☐	☒	Flow cytometry
☒	☐	MRI-based neuroimaging

Antibodies

Antibodies used	FITC anti-mouse CD45 Antibody BioLegend 103108 FACS<br>Brilliant Violet 421™ anti-mouse/human CD11b Antibody BioLegend 101236 FACS<br>APC anti-mouse F4/80 Antibody BioLegend 123116 FACS<br>PE anti-mouse CD11c Antibody BioLegend 117308 FACS<br>INS Monoclonal antibody Proteintech 66198-1-Ig Immunofluorescence<br>Cleaved Caspase-3 (Asp175) Cell Signaling 9661 Immunofluorescence<br>Goat anti-Rabbit Alexa Fluor™ 568 Cat # A-11011,<br>Goat anti-Mouse Alexa Fluor™ 488 Cat # A-11001<br>MSI2 Polyclonal antibody Proteintech 10770-1-AP Western blot<br>HNRNPA3 Polyclonal antibody Proteintech 25142-1-AP Western blot<br>Goat Anti-Rabbit IgG (H + L)-HRP Conjugate 1706515 Biorad<br>Goat Anti-Mouse IgG (H + L)-HRP Conjugate 1706516 Biorad
Validation	Primary antibodies were obtained from the aforementioned companies. Testing was performed to control for specificity and efficiency. Company websites and literature were consulted for selecting the antibodies according to their use for immunocytochemistry/immunofluorescence (ICC/IF) or western blot (WB) and here descibed. FITC anti-mouse CD45 Antibody BioLegend 103108: Verified Reactivity Mouse, Application References 1. Podd BS, et al. 2006. J. Immunol. 176:6532. (FC, CMCD) PubMed 2. Haynes NM, et al. 2007. J. Immunol. 179:5099. (FC). Brilliant Violet 421™ anti-mouse/human CD11b Antibody BioLegend 101236 FACS - Reactivity Mouse,Human,Cynomolgus,Rhesus Application References Springer T, et al. 1978. Eur. J. Immunol. 8:539. APC anti-mouse F4/80 Antibody BioLegend 123116 Verified Reactivity Mouse, Application References: Kobayashi M, et al.2008. J. Leukoc. Biol. 83:1354. Poeckel D, et al. 2009. J. Biol Chem. 284:21077. PE anti-mouse CD11c Antibody BioLegend 117308 Verified Reactivity Mouse, application references: Granucci F, et al. 1997. J. Immunol. 159:1794. Stokes RW, et al. 1998. J. Immunol. 160:5514. INS Monoclonal antibody Proteintech 66198-1-Ig Immunofluorescence, Tested applications: Positive IHC detected in human pancreas tissue, mouse pancreas tissue, rat pancreas tissue; Positive IF-P detected in human pancreas tissue, mouse pancreas tissue. Application reference: Metabolic effects of CCL5 deficiency in lean and obese mice- Hui Zhou. Cleaved Caspase-3 (Asp175) Cell Signaling 9661 Immunofluorescence, REACTIVITY H M R Mk, Apllication Reference: Journal: Clin Transl Radiat Oncol In vitro determination of patient-specific variation challenges the universal RBE gold standard for ...Author: Anna Kirstein, et al. Year: 2026. MSI2 Polyclonal antibody Proteintech 10770-1-AP, Tested Applications Positive WB detected in PC-12 cells, C6 cells, COLO 320 cells, MCF-7 cells Positive IP detected in MCF-7 cells. Application reference for WB,IF Science Neurodevelopmental protein Musashi-1 interacts with the Zika genome and promotes viral replication. Authors - Pavithra L Chavali. HNRNPA3 Polyclonal antibody Proteintech 25142-1-AP, Tested Applications Positive WB detected in mouse liver tissue, Jurkat cells, Application reference for WB: Nat Commun. A network of RNA-binding proteins controls translation efficiency to activate anaerobic metabolism. Authors - J J David Ho.

Eukaryotic cell lines

Policy information about cell lines and Sex and Gender in Research

Cell line source(s)	MIN6B1 cells, a subclone of the murine insulin-secreting cell line derived from insulinoma (Ref 63), were kindly provided by Dr. Philippe Halban, DepartmentMédecine Génétique & Développement Faculté de Médecine, Switzerland.
Authentication	The secretory capacity of MIN6B1 cells to release insulin in response to glucose or other secretagogues was verified after each thaw and frequently during passaging. The cell line was not further authenticated.
Mycoplasma contamination	MIN6B1 cells tested negative for mycoplasma contamination.
Commonly misidentified lines (See ICLAC register)	No commonly misidentified cell lines were used in the study.

Animals and other research organisms

Policy information about studies involving animals; ARRIVE guidelines recommended for reporting animal research, and Sex and Gender in Research

Laboratory animals	male C57BL/KsJ db/db mice aged 8 weeks and their respective wild type age-matched controls, male C57BL/KsJ db/db and C57BL/6J ob/ob mice, and their respective control db +/- and ob -/- mice 13-16 weeks, wild type 17 week-old male C57BL/6J fed high fat or normal diet for diet induced obesity model. Male and female wild type 12-14 week-old C57BL/6k for primary pancreatic islet and bone marrow isolation . Mice were housed on a 12-h light/dark cycle with mean ambient temperature of 20-21 °C and 55% humidity with ad-libitum chow diet (SAFE -150-SP).
Wild animals	The study did not involve wild animals.
Reporting on sex	Male genetic modified mice and their age-matched controls were used for this study. Both male and female wild type mice were used for post-mortem organ collection and primary cell culture: 50% of the preparation derived from male and 50% from female. Separate analysis based on sex were not performed for this study.
Field-collected samples	The study did not involve samples collected from the field.
Ethics oversight	Procedures were performed in agreement with the NIH guidelines and approved by the Swiss Research Councils and Veterinary Offices under the animal authorization number VD2495x4 and veterinary offices and the national health and medical research council of Australia.

Note that full information on the approval of the study protocol must also be provided in the manuscript.

Clinical data

Policy information about clinical studies

All manuscripts should comply with the ICMJE guidelines for publication of clinical research and a completed CONSORT checklist must be included with all submissions.

Clinical trial registration	The observational ClinicalTrials.gov NCT02175459 is a cross-sectional study approved by the Ethical Committee Fondazione Policlinico Universitario Agostino Gemelli IRCCS – Università Cattolica del Sacro Cuore (P/656/CE2010 and 22573/14), and all participants provided written informed consent
Study protocol	Design Details<br>Observational Model : Cohort<br>Time Perspective: Cross-Sectional<br>Biospecimen Retention: Samples Without DNA<br>Biospecimen Description: PANCREAS SAMPLES<br>Prior to surgery in vivo studies were performed (Hyperglycemic clamp, Euglycemic Hyperinsulinemic clamp and Mixed Meal Tests) to accurately assess glucose homeostasis parameters to classify each individual into metabolic phenotypes.<br><br>Specifically, the patients were subjected to incretin secretion (mixed meal), metabolic status (OGTT), insulin secretion characteristics (first and second phase responses), β-cell insulin content evaluation (arginine bolus). Subsequently, pancreas samples were evaluated for morphometry, and proteomics and gene expression analyses of islet cell samples obtain by laser capture will allow a detailed investigation of mechanisms that contribute to islet plasticity.<br>Full study protocol is accessible on ClinicalTrials.gov under accession number NCT02175459
Data collection	living donors undergoing pylorus-preserving pancreatoduodenectomy as part of their clinical care for neoplasms, recruited at the Digestive Surgery Unit and studied at the Centre for Endocrine and Metabolic Diseases Unit (Agostino Gemelli University Hospital, Rome, Italy) were subjected to in vivo studies (Hyperglycemic clamp, Euglycemic Hyperinsulinemic clamp and Mixed Meal Tests) to accurately assess glucose homeostasis parameters to classify each individual into metabolic phenotypes. Pancreas samples collected from these patients were used for islet laser capture microdissection and small RNA sequencing studies performed at the University of Siena and University of Lausanne.

	Study Start 2010-08 Primary Completion 2024-12 Study Completion 2024-12<br>Study Type : Observational
Outcomes	The primary outcome of the trial was the definition of metabolic status determined with oral glucose tolerance test and patients classification according their metabolic status. Secondary outcomes included identification of changes in intraislet tRNA-derived fragments in different metabolic status. and correlations between specific tRF levels and metabolic traits. Outcome measures were defined prior to statistical analysis. tRF abundance was quantified by small RNA sequencing and normalized counts were used for statistical modeling with clinical variables obtained from donor records.

Plants

Seed stocks	*Report on the source of all seed stocks or other plant material used. If applicable, state the seed stock centre and catalogue number. If plant specimens were collected from the field, describe the collection location, date and sampling procedures.*
Novel plant genotypes	*Describe the methods by which all novel plant genotypes were produced. This includes those generated by transgenic approaches, gene editing, chemical/radiation-based mutagenesis and hybridization. For transgenic lines, describe the transformation method, the number of independent lines analyzed and the generation upon which experiments were performed. For gene-edited lines, describe the editor used, the endogenous sequence targeted for editing, the targeting guide RNA sequence (if applicable) and how the editor was applied.*
Authentication	*Describe any authentication procedures for each seed stock used or novel genotype generated. Describe any experiments used to assess the effect of a mutation and, where applicable, how potential secondary effects (e.g. second site T-DNA insertions, mosiacism, off-target gene editing) were examined.*

Flow Cytometry

Plots

Confirm that:

☒ The axis labels state the marker and fluorochrome used (e.g. CD4-FITC).

☒ The axis scales are clearly visible. Include numbers along axes only for bottom left plot of group (a 'group' is an analysis of identical markers).

☒ All plots are contour plots with outliers or pseudocolor plots.

☒ A numerical value for number of cells or percentage (with statistics) is provided.

Methodology

Sample preparation	For fluorescence-Activated Cell Sorting (FACS), islets from two db/db mice and three wild type mice were pooled; 600-800 islets per preparation were dissociated. Dissociated islet cells were washed once with FACS buffer (0.1% BSA, 2mM EDTA, 11mM glucose in PBS) and incubated for 5 minutes with TruStain FcX™ (anti-mouse CD16/32) Antibody (BioLegend), at 4°C. Then cells were incubated for 30 min in the dark at 4°C with the following antibodies: 1:200 of FITC anti-CD45, 1:100 brilliant violet CD11b, 1:100 APC F4/80 and 1:100 PE CD11c (BioLegend). Cells were then washed twice with FACS buffer and sort by FCF-Aria-II (SORP).
Instrument	FCF-Aria-II (SORP)
Software	FlowJo
Cell population abundance	The Cd11c+ macrophages were <1% of the total events, Beta cell-fraction collected resulted in 15-20% of the total event number. Purity was assessed by immunofluorescence and gene marker expression by qPCR.
Gating strategy	Cells were first gated based on forward scatter (FSC) and side scatter (SSC) parameters to exclude debris and select the main cellular population. Doublets were excluded using FSC-A versus FSC-H gating.<br>Immune cells were identified based on CD45 expression, with positive and negative boundaries defined using unstained controls. $CD45^+$ cells were retained for immune cell analysis, while $CD45^-$ cells were used for endocrine cell sorting.<br>Within the $CD45^-$ fraction, β cells were identified and sorted based on their characteristic high intrinsic autofluorescence. Autofluorescence-positive and -negative gates were defined using the bimodal fluorescence distribution observed in the relevant channels and validated using control populations, with gates set to minimize overlap between low- and high-autofluorescent cells.<br>Within the $CD45^+$ immune compartment, myeloid cells were identified by expression of CD11b. Macrophages and dendritic-like cells were subsequently defined based on expression of F4/80 and CD11c. Positive and negative boundaries for CD11b, F4/80, and CD11c were established using unstained controls. Cells co-expressing F4/80, CD11b, and CD11c were gated as $F4/80^+$ $CD11b^+$ $CD11c^+$ cells.

☒ Tick this box to confirm that a figure exemplifying the gating strategy is provided in the Supplementary Information.
