# Supplementary material for: 5’tRNA-derived fragments modulate β-cell homeostasis and islet macrophage activation in type 2 diabetes
Source: Nat Commun. 2026 May 4;17:5989. doi: 10.1038/s41467-026-72641-z (PMC13346763; doi:10.1038/s41467-026-72641-z)
Supplement: Supplementary file 6 — Transparent Peer Review file [file 41467_2026_72641_MOESM6_ESM.pdf]

nature portfolio

Peer Review File

5’tRNA-derived fragments modulate β-cell homeostasis and islet macrophage activation in type 2 diabetes

Corresponding Author: Dr Cristina Cosentino

This file contains all reviewer reports in order by version, followed by all author rebuttals in order by version.

Version 0:

Reviewer comments:

Reviewer #1

(Remarks to the Author)
The manuscript of Cosentino et al examines the effects of 5’tRNA fragments on beta cell homeostasis and macrophages in the context of type 2 diabetes. Briefly, the manuscript reports that stressed beta cells over-produce transfer RNA-derived fragments from the 5’ end of the Glu and Gly tRNAs. This is seen in a mouse model of type 2 diabetes, as well as in laser-capture micro-dissected islets from human samples. In a co-culture model, they show these fragments induce macrophage activcation via interacting with RNA-binding proteins.

General comments

The manuscript has a high degree of novelty and is technically sound.

The paper is well written and clear.

The figures are generally clear.

The use of human islets, including from the living donor cohort, is important for translation. It would be nice to know whether these fragments can be detected in any of the larger online databases of islets omics.

Fatty acid treatment promotes cell stress, but in human islets can sometimes also acutely increase insulin secretion. What are the acute effects of fatty acid treatments on insulin secretion in mouse and human islets under the conditions where fragments are produced? What about effects on protein synthesis rates in these time scales? Analysis of protein synthesis would strengthen the paper or the authors could list it as a limitation of their study.

What are the cell autonomous mechanisms by which the fragments affect beta cell survival?

The identification of Musashi 2 as a potential binding partner and mediator of the effects of 5’tRFGLU(CTC) is interesting. Is this a cause or effect of beta cell ER stress in this model? Or both?

Specific comments

In Figure 3. The authors should show the insulin secretion without normalizing to insulin content, since the later (partially independent) parameter changes with palmitate treatment.

Some of the paragraphs are too short, containing only one or two sentences.

Reviewer #2

(Remarks to the Author)

Cosentino et al. reported the pathogenic role of 5'tRNA-derived fragments in islet dysfunctions during the development of T2DM. This study suggests that the increase in 5'RFGlu(CTC) abundance and its involvement in the crosstalk between beta cells and islet macrophages contribute to T2DM-related islet abnormalities. However, questions/concerns described as follow could challenge the conclusions of this study:

1) Need more introduction on tRFs. especially the information regarding tRNAGlu(CTC), tRNAGly(GCC), tRNAAsp(CTC).
2) In Fig 1b and d, how were beta cells prepared for these assays? From beta cell-specific reporter mice?
3) For Fig 1f data, please clarify the method of qPCR data normalization by Let-7e and miR-16, respectively. This information should be included in the method section.
4) The expression pattern of 5'tRFGlue(CTC) should be evaluated in beta cells and iMAC of high-fat diet-induced obese models.
5) Beta cells used in Fig 2e and 2f were sorted from beta cell-specific reporter mice?
6) What would be the mechanisms underlying the elevation of ANG expression in db/db or in vitro fatty acid treatment?
7) Fig 3e-g, any significant difference in glucose-stimulated insulin secretion between Ctr-PA and aGlu-PA groups?
8) The data in Fig 3f and 3g show that blocking 5'tRF had minimal effects on the impact of prolonged PA treatment. Does it mean decreasing 5'tRF may not attenuate beta cell abnormalities in obese mice?
9) Fig 4a, would PA affect 5'tRF abundance in BMDMs? Without co-cultured with Min6 cells.
10) For the M0 control in Fig 4b, were these cells also on the dish for 3days without beta cell co-culture?
11) For the data in Fig 4d, it is hard to separate the effects of beta cells and PA on iMAC-like BMDM 5'RFGlu(CTC) expression. For example, the increase in 5'RFGlu(CTC) in iMAC-like BMDMs from PA Ctr group was induced by PA or Min6 cells? or both? Similarly, the data in Fig 4f.
12) Need to clarify the experiment design in Fig 4e: data from BMDM+Min6 coculture or just BMDMs alone culture?
13) In addition to the data in Fig 4e, would aGlu treatment affect BMDM proinflammatory cytokine expression after co-culturing with Min6 cells without PA treatment?
14) What would be the mechanisms underlying the phenotypes in Figure 4? Especially the role of 5'tRF in the interaction between beta cells and BMDMs in the presence of PA.
15) Most of data related to 5'tRFGlu(CTC) functions were derived from in vitro assays. These phenotypes should be confirmed with appropriate in vivo cell-specific 5'tRF KO models.
16) Would be 5'tRFGlu(CTC) interactors similar in iMAC? Especially iMAC isolated from HFD-fed or db/db mice.
17) Effects of 5'tRFGlu(CTC) on islet functions should also be validated with human islets.

Reviewer #3

(Remarks to the Author)
Overall comments
This manuscript identifies and characterises a novel mechanism by which a small RNA fragment derived from tRNA (tRF) can mediate pathological responses to lipotoxic inducers of diabetes in an animal model. The study is a good demonstration of the unexplored potential of these small RNAs to mediate complex disease, further expands their possible mechanisms of action, and provides additional importance to the field and continued study. The study does not demonstrate that this pathway (derived from a mouse model) is directly comparable to human disease, but at the least demonstrates that the tRFs are present and dynamically active and correlated with disease. I believe this is not an issue as the animal models of T2D are robust and known to be representative of human disease. Overall, a very well-written, researched and presented study which is more than worthy of publication with a few minor alterations and clarifications. Well done.

Specific comments
• In general, the use of square heatmaps/dotplots is a bit confusing as the size of the squares does not carry immediately clear information. Would suggest adding a size legend for the IBAC levels to perhaps improve clarity.
• On volcano plot, it would be useful to annotate some or all of the top genes/proteins/elements to demonstrate their relationship to all molecules
• It would be useful to see similar transcriptional profiles occurring in the human samples as seen in the tRF ASO knockdowns – particular in the different classes of disease (i.e. NGT, IGT, T2D). This would draw a stronger link between the model and human pathophysiology. This is by no means an essential link to draw, but would strengthen the case for these processes dynamically occurring in human disease.

Line 98
Vast majority of 5' tRFs are down-regulated in B cells. Is there an explanation for this?

Line 103
Were the cleavage sites and lengths of the 5' tRFs consistent? Are they similar to other studies demonstrating the functonality of these tRFs

Fig1j-k
Is the Log2 “Cts” the correct axis? I would interpret higher “Cts” as qPCR data where lower levels would indicate the relationship is positive. If this is shorthand for RNAseq counts, I would change to “counts” just to avoid any possible confusion with qPCR.

Line 128
Whats the thinking on the potential for increased tRNA production preceding the increased fragmentation into a tRF? I.e.

could the increase in tRF be a general by-product of the cellular response to lipotoxic stress. A look at full-length tRNA responses would be valuable here.

Line 134
Interested if the tRF levels return to baseline after removal of the stress? This might yield insight into the potential for therapeutics to ameliorate the effect.

Line 166
This statement needs expansion. Why?

Line 188. Typo:
prior TO co-culture

Fig5b, Line 217
Some of the proteins, eg.(Hnrnpa3/b1m Srs3/7 etc) exhibited quite high IBAQ levels in the control IP. I interepret this as they are likely generalised binders of RNA fragments and are unlikely to be mediated specific cellular functions. Also, given the data in 5b for these proteins, would it not be unreasonable to expect some detection in the WB (5c) - particularly for HnRNP-A3. A higher exposure of the WB should identify this.

Fig S6. C1/2
These references are reversed? Up is C1, down is C2. May be worth checking all.
Line 286-289
The supplementary figure does not show tRF levels (Fig7a) - this is a bit confusing. I understand this reference the characterisation of the macrophage phenotypes.

Version 1:

Reviewer comments:

Reviewer #1

(Remarks to the Author)
The authors have made an already strong paper even better with this revision.

Reviewer #2

(Remarks to the Author)
My concerns/questions are addressed appropriately, no additional comment.

Reviewer #3

(Remarks to the Author)
Thank you to the authors for comprehensively addressing my questions. I am thoroughly impressed with the attention to detail and more than happy for this publication to proceed.

Open Access This Peer Review File is licensed under a Creative Commons Attribution 4.0 International License, which permits use, sharing, adaptation, distribution and reproduction in any medium or format, as long as you give appropriate credit to the original author(s) and the source, provide a link to the Creative Commons license, and indicate if changes were made.
In cases where reviewers are anonymous, credit should be given to 'Anonymous Referee' and the source.
The images or other third party material in this Peer Review File are included in the article's Creative Commons license, unless indicated otherwise in a credit line to the material. If material is not included in the article's Creative Commons license and your intended use is not permitted by statutory regulation or exceeds the permitted use, you will need to obtain permission directly from the copyright holder.
To view a copy of this license, visit https://creativecommons.org/licenses/by/4.0/
